# Expression and clinical significance of interleukin-6 pathway in cholangiocarcinoma

**DOI:** 10.3389/fimmu.2024.1374967

**Published:** 2024-05-31

**Authors:** Dongqing Gu, Xin Zhao, Jing Song, Jianmei Xiao, Leida Zhang, Guohong Deng, Dajiang Li

**Affiliations:** ^1^ Department of Infectious Diseases, First Affiliated Hospital, Army Medical University, Chongqing, China; ^2^ Chongqing Key Laboratory of Viral Infectious Diseases, Chongqing, China; ^3^ Department of Hepatobiliary Surgery, First Affiliated Hospital, Army Medical University, Chongqing, China; ^4^ Ministry of Education Key Laboratory of Child Development and Disorders, Children’s Hospital of Chongqing Medical University (CHCMU), Chongqing, China

**Keywords:** cholangiocarcinoma, multiplex immunofluorescence, carcinogenesis, prognosis, IL-6/STAT3 pathway, tissue microarray

## Abstract

**Background:**

Cholangiocarcinoma (CCA) is a typical inflammation-induced malignancy, and elevated serum interleukin-6 (IL-6) levels have been reported to be linked to the onset and progression of CCA. We aim to investigate the potential prognostic value of the IL-6 pathway for CCA.

**Methods:**

We detected the expressions of IL-6, IL-6R, glycoprotein (gp130), C-reactive protein (CRP), Janus kinase 2 (JAK2), and signal transducer and activator of transcription 3 (STAT3) in CCA tissue microarray using multiplex immunofluorescence. Furthermore, the clinical associations and prognostic values were assessed. Finally, single-cell transcriptome analysis was performed to evaluate the expression level of IL-6 pathway genes in CCA.

**Results:**

The results revealed that the expression of IL-6 was lower, while the expression of STAT3 was higher in tumor tissues compared to normal tissues. Especially in tumor microenvironment, the expression of IL-6 pathway genes was generally downregulated. Importantly, gp130 was strongly correlated with JAK2 in tumor tissues, while it was moderately correlated with JAK2 in normal tissue. Although none of the gene expressions were directly associated with overall survival and disease-free survival, our study found that IL-6, IL-6R, CRP, gp130, and JAK2 were inversely correlated with vascular invasion, which is a risk factor for poor prognosis in patients with CCA.

**Conclusion:**

The findings from this study suggest that the IL-6 signaling pathway may have a potential prognostic value for CCA. Further investigation is needed to understand the underlying molecular mechanisms of the IL-6 pathway in CCA.

## 1 Introduction

Cholangiocarcinoma (CCA) is the second most common type of liver cancer, representing 10-15% of all primary liver malignancies (1). Both the incidence and mortality rates related to this condition have been increasing globally over the past few decades (2). Surgical resection remains the potentially curative treatment for CCA, whereas most patients are diagnosed at an advanced stage and miss the opportunity for surgery. As a highly lethal adenocarcinoma of the hepatobiliary system, the 5-year overall survival (OS) rate is only 5% to 17% (3). Therefore, it is crucial to promptly identify new biomarkers and develop therapeutic strategies for this cancer.

CCA is a common malignancy caused by inflammation, and persistent inflammation plays a significant role in the onset and progression of CCA. Chronic inflammation of the biliary tract due to choledocholithiasis, cholelithiasis, or primary sclerosing cholangitis is a significant risk factor for CCA (4). Other risk factors for cholangiocarcinoma, such as bile duct stones and infections (salmonella, hepatitis B virus, liver fluke, etc.), can lead to chronic inflammatory reactions (5).

The interleukin-6 (IL-6) pathway is an important inflammatory pathway that plays a critical role in mediating downstream inflammatory cascade reactions. The classical pathway is initiated by the binding of IL-6 to the membrane-bound IL-6 receptor (mIL-6R) and glycoprotein 130 (gp130) signal-transducing subunit, while the trans pathway is initiated by the binding of soluble IL-6R (sIL-6R) signal via membrane-bound gp130 (6). At present, intracellular signaling of IL-6 in response to receptor activation is mainly through IL-6-dependent activation of the Janus kinase (JAK)- signal transducer and activator of transcription (STAT) pathway, the mitogen activated protein kinase (MAPK) cascade, and the phosphatidylinositide-3-kinase (PI3K) cascade (7). IL-6 is a multifunctional cytokine that is abundant in the tumor microenvironment. The abnormally activated IL-6/JAK2/STAT3 signaling pathway can contribute to the onset and progression of malignant tumors by affecting the proliferation, migration, invasion, angiogenesis, and apoptosis of tumor cells (8). Currently, this pathway has been linked to the onset and progression of liver cancer, lung cancer, gastric cancer, breast cancer, and other malignant tumors (9–13).

The impact of IL-6 serum levels on CCA has been well established. Goydos et al. reported that serum IL-6 levels in patients with CCA were elevated and correlated with tumor burden before and after resection, indicating their potential as clinical biomarkers (14). Subsequently, Cheon and Porta et al. also demonstrated elevated serum IL-6 levels in patients with liver cancer and CCA, suggesting that it may serve as a potential diagnostic marker for these conditions (15, 16). In particular, IL-6 was found to be associated with the tumor volume of CCA. Inhibiting the IL-6 signaling pathway might serve as a potential therapeutic target (17, 18).

However, the protein expression of the IL-6 pathway in tumor tissue has been scarcely addressed. Therefore, this study aims to identify the expressions of IL-6, IL-6R, glycoprotein (gp130), C-reactive protein (CRP), Janus kinase 2 (JAK2), and signal transducer and activator of transcription 3 (STAT3) in CCA tissue microarray (TMA) using multiplex immunofluorescence (mIF). Furthermore, the clinical associations and prognostic values were assessed. This study aims to identify potential prognostic biomarkers for CCA and provide novel targets for the treatment of this fatal cancer.

## 2 Materials and methods

### 2.1 Ethics approval

The present study has been approved by the ethics committee of the Shanghai Outdo Company (HIBDA180Su02).

### 2.2 TMA and mIF

TMA was purchased from Shanghai Outdo Company (Shanghai, China), which included 91 CCA tumor tissues and 31 normal tissues. Normal tissue is the adjacent normal bile duct tissue of CCA patients. Multiplexed immunofluorescence staining of TMA was performed using the Opal 7-color fluorescent IHC kit (PerkinElmer, Waltham, USA) with two panels of antibodies. Panel 1 contains antibodies against IL6 (1:200 dilution, Proteintech, 69001-1-Ig), GP130 (1:100 dilution, Proteintech, 67679-1-Ig), JAK2 (1:3000 dilution, abclonal, A11497), and Cytokeratin 19 (CK19) (GeneTech, GM088807), and panel 2 contains antibodies against IL-6R (1:500 dilution, Proteintech, 66855-1-Ig), CRP (1:500 dilution, Proteintech, 66250-1-Ig), STAT3 (1:500 dilution, Proteintech, 60199-1-Ig) and CK19 (GeneTech, GM088807). CK 19 was used to label epithelial cancer cells, while 2-(4-Amidinophenyl)-6-indolecarbamidine dihydrochloride (DAPI) was used to label the nucleus. EP region was defined as CK 19 positive, while SA region was defined as CK 19 negative. Briefly, we first optimize the concentration and staining sequence of the antibodies. Then, the slides were baked at 63°C for 1 hour and dewaxed with xylene for 10 minutes followed by rehydration in 100%, 90%, 80%, and 70% ethanol for 5 minutes each time. Subsequently, TMA slides were processed with a microwave for antigen retrieval, after incubation with H_2_O_2_ for 10 minutes, the slides were blocked with a blocking buffer for 10 min at room temperature. After antigen retrieval, the slides were incubated with antigen-specific primary antibodies and secondary antibodies followed by Opal for coloration. Thereafter, the antibodies were removed by microwave treatment before another round of staining was performed. Finally, we added DAPI to stain the nucleus.

### 2.3 Fluorescence signal quantification

Visualization and quantitation of the fluorescence signal were assessed with the Tissue-FAXS system (TissueGnostics Asia Pacific Limited, Australia) and Strata-Quest analysi**s** software (Version No. 7.0.1.165, TissueGnostics Asia Pacific Limited, Australia). Firstly, we used a spectral library for spectral resolution to obtain a single-channel fluorescence signal. Then, the DAPI channel was used to identify an effective nucleus. Using the effective nucleus as the core, we set a threshold based on the staining situation of each protein, divided the protein expression positive cell population, and counted the positive cells. We also counted the number and intensity of double-positive cells. Finally, we used the mean intensity to multiply the percentage of positive cells to represent the protein expression levels.

### 2.4 RNA expression in TCGA

The gene expression of IL-6, IL6R, gp130, CRP, JAK2, and STAT3 were analyzed in the Cancer Genome Atlas (TCGA, http://cancergenome.nih.gov/). TCGA Bile Duct Cancer (CHOL) cohort which included 36 CCA tumor samples and 9 normal samples was obtained. Then, we performed a single scatterplot, boxplot, and survival analysis between these genes and clinical characteristics.

### 2.5 Single-cell transcriptome analysis

Furthermore, we evaluate the expression level of IL6 family genes in CCA at the single-cell solution, single-cell data of CCA tissues (n=9) from Gene Expression Omnibus (GEO, accession id: GSE189903). Cells with < 500 genes detected and genes expressed in < 3 cells were removed for quality control. Cell type annotations (malignant cells or other stomal/immune cell types) were obtained from the original literature (19). Seurat (v5.0.1) package in R was used for analysis of single-cell RNA-seq data. The expression difference of IL-6, IL6R, gp130, CRP, JAK2, and STAT3 in malignant cells or non-malignant cells between CCA tumor tissues and adjacent normal tissues were analyzed. The vascular invasion, defined as gene expression signature of vascular invasion (20),which was obtained to calculate single-sample gene set enrichment analysis (ssGSEA) score based on GSVA (v1.46.0) R package for each malignant cell.

### 2.6 Statistical analysis

Continuous variables were described as means ± standard deviations or medians [with interquartile ranges (IQRs)] and evaluated using the Student’s *t*-test (normally distributed data) or Mann-Whitney *U* test (nonnormally distributed data), while categorical variables were presented as number (percentage) and evaluated using the χ2 test or Mann-Whitney *U* test for ranked data. The correlation analyses were assessed by Spearman regression. The mean of gene expression was used as a cut-off (high versus low). The survival analysis was performed using the Kaplan–Meier method and the log-rank test was used for group comparisons. The univariable and multivariable Cox proportional hazards regressions were used to estimate the hazard ratio (HR) or adjusted HR (aHR) and 95% confidence interval (CI). Statistical analyses were conducted using IBM SPSS Statistics Version 23.0 (IBM Corp., Armonk, NK, USA), and a 2-tailed *P* value < 0.05 was considered statistically different.

## 3 Results

### 3.1 Clinical baseline description of the TMA

We evaluated the protein expression level of IL-6, IL6-R, gp130, CRP, JAK2, and STAT3 by mIF using TMA. As shown in **Supplementary Table 1**, a total of 91 CCA patients were involved in the study, including 48 males (52.7%) and 43 females (47.3%), with a mean age of 56.20 ± 9.83 years. There were 11 (12.1%) stage I, 34 (37.4%) stage II, 33 (36.3%) stage III, and 13 (14.3%) stage IV according to tumor node metastasis (TNM) stages. Tumor differentiation was low for 16 (17.6%), medium for 68 (74.7%), and high for 6 (6.6%). In addition, one patient was identified as having mucinous cystadenocarcinoma. There were 65 (71.4%) patients within this cohort whose tumors had recurred after the surgery. The survival time ranged from 1 to 117 months, with a median OS time of 16 (6 - 46) months and a median disease-free survival (DFS) time of 11.5 (4.0 - 33.8) months.

### 3.2 The expression of IL-6, IL6-R, gp130, CRP, JAK2, and STAT3

**Figure 1:**
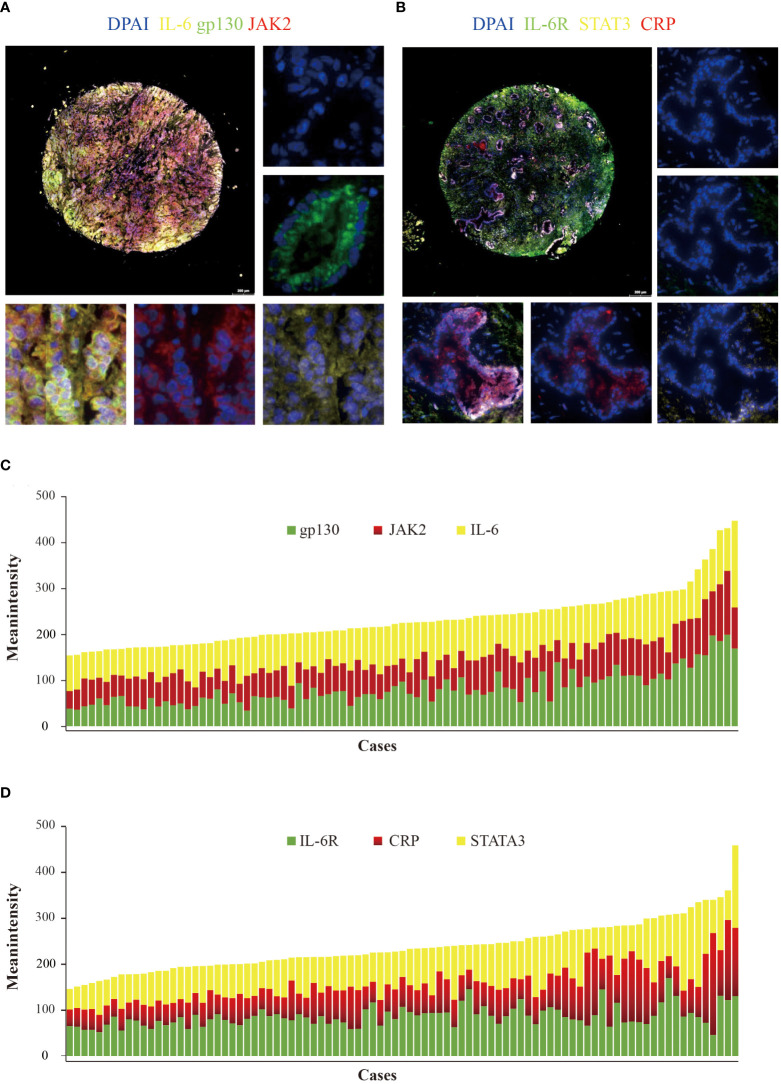
Detection of IL-6, IL-6R, gp130, JAK2, STAT3, and CRP in cholangiocarcinoma using multiplex quantitative fluorescence. **(A)** Representative fluorescence pictures showing IL-6 (yellow), gp130 (green), and JAK2 (red). **(B)** Representative fluorescence pictures showing IL-6R (green), STAT3 (yellow), and CRP (red). **(C)** Distribution of IL-6 (yellow), gp130 (green), and JAK2 (red) mean intensity scores. **(D)** Distribution of IL-6R (green), STAT3 (yellow), and CRP (red) mean intensity scores.

Gp130 and CRP mainly expressed in bile duct epithelial cells (**Figures 1A, B**). IL-6, IL6-R, gp130, CRP, JAK2, and STAT3 were detected in all the cases. Meanwhile, IL-6 (mean intensity = 86.4, range from 52.5 to 188.7) and IL-6R (mean intensity = 89.0, range from 48.3 to 172.1) showed prominently high intensity, while gp130 (mean intensity = 78.0, range from 33.9 to 199.0), JAK2 (mean intensity = 63.4, range from 38.4 to 138.9), STAT3 (mean intensity = 79.9, range from 44.7 to 184.2), and CRP (mean intensity = 76.4, range from 36.5 to 222.2) showed relatively low intensity (**Figures 1C, D**).

**Figure 2:**
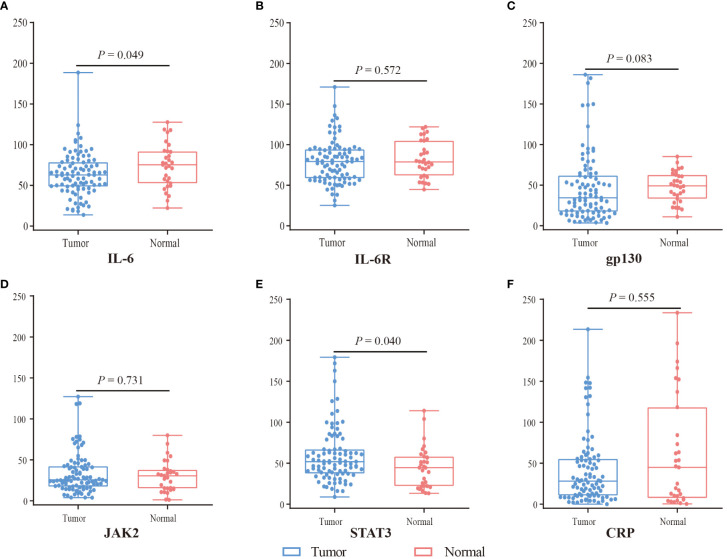
The expression difference of IL6 **(A)**, IL6R **(B)**, gp130 **(C)**, JAK2 **(D)**, STAT3 **(E)**, and CRP **(F)** between tumor (blue) and normal tissue (red) by mIF. Visualization and quantitation of the fluorescence signal were assessed with the Tissue-FAXS system and Strata-Quest analysis software. Mean intensity to multiply the percentage of positive cells to represent the protein expression levels. Mann-Whitney *U* test was used to compare the difference, and a 2-tailed *P* value < 0.05 was considered statistically different.

The expression of IL-6 was lower in tumor tissues (N = 91) compared with normal tissues (N = 31) (*P* = 0.049), whereas the expression of STAT3 was higher (*P* = 0.040) (**Figure 2**). However, there was no statistical difference in the expression of gp-130, IL-6R, CRP, and JAK2 (*P* > 0.05). In SA region, the expression of CRP (*P* = 0.048), gp-130 (*P* < 0.001), JAK2 (*P* = 0.004), and IL-6 (*P* = 0.001) was lower in tumor tissues compared with normal tissues, while the expression of IL-6R (*P* = 0.191) and STAT3 (*P* = 0.960) was comparable (**Supplementary Figure 1**). However, there was no statistical difference in the expression of IL-6, IL6-R, gp130, CRP, JAK2, and STAT3 (all *P* > 0.05) in EP region. Furthermore, we evaluated the gene expression of IL-6, IL-6R, gp130, CRP, JAK2, and STAT3 in TCGA with 36 CCA tissues and 9 normal tissues by GEPIA (http://gepia.cancer-pku.cn/). The expression of IL-6R was lower, whereas gp130 was higher in CCA tissues than in normal tissues (*P* < 0.05) (**Supplementary Figure 2**).

**Figure 3:**
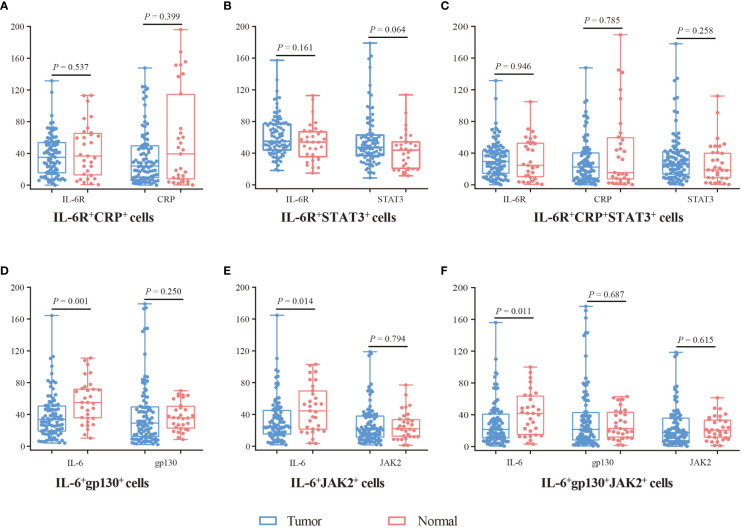
The expression difference of IL-6, IL-6R, gp130, JAK2, STAT3, and CRP in double-positive and triple-positive cells. **(A)** The expression of IL-6R and CRP in IL-6R^+^CRP^+^ cells. **(B)** The expression of IL-6R and STAT3 in IL-6R^+^STAT3^+^ cells. **(C)** The expression of IL-6R, CRP, and STAT3 in IL-6R^+^CRP^+^STAT3^+^ cells. **(D)** The expression of IL-6 and gp130 in IL-6^+^gp130^+^ cells. **(E)** The expression of IL-6 and JAK2 in IL-6^+^JAK2^+^ cells. **(F)** The expression of IL-6, gp130, and JAK2 in IL-6^+^gp130^+^JAK2^+^ cells. Visualization and quantitation of the fluorescence signal were assessed with the Tissue-FAXS system and Strata-Quest analysi**s** software. Mean intensity to multiply the percentage of positive cells to represent the protein expression levels. Mann-Whitney *U* test was used to compare the difference, and a 2-tailed *P* value < 0.05 was considered statistically different.

In addition, we analyzed the expression of these proteins in double-positive and triple-positive cells (**Figure 3**). In IL-6^+^gp130^+^, IL-6^+^JAK2^+^, and IL-6^+^gp130^+^JAK2^+^ cells, the expression of IL-6 was lower in tumor tissues compared with normal tissues (*P* < 0.05), while the expression of JAK2 and gp130 was comparable (*P* > 0.05). In IL-6R^+^CRP^+^, IL-6R^+^STAT3^+^, and IL-6R^+^CRP^+^STAT3^+^cells, the expression of IL-6R, CRP and STAT3 was comparable (*P* > 0.05). In SA region, the expression of IL-6, gp130, and JAK2 was lower in tumor tissues in IL-6^+^gp130^+^, IL-6^+^JAK2^+^, and IL-6^+^gp130^+^JAK2^+^ cells (*P* < 0.05); and the expression of IL-6R, CRP was also lower in tumor tissues in IL-6R^+^CRP^+^, IL-6R^+^STAT3^+^, and IL-6R^+^CRP^+^STAT3^+^cells (*P* < 0.05) (**Supplementary Figure 3**). However, no statistical difference was observed in the expression of IL-6 family in EP region (all *P* > 0.05).

### 3.3 The correlation between IL-6, IL-6R, gp130, CRP, JAK2, and STAT3

**Figure 4:**
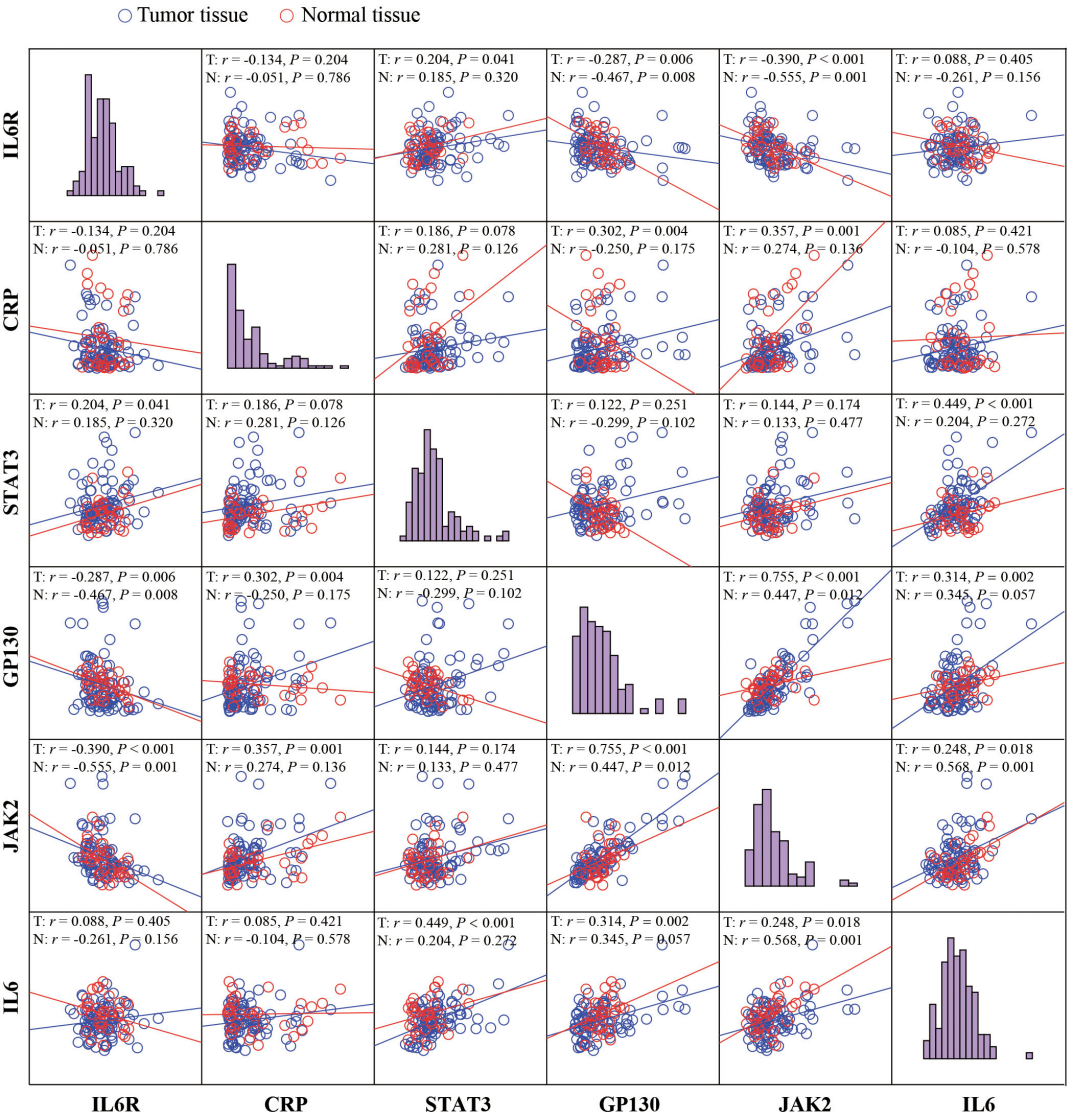
The correlation analyses between IL-6, IL-6R, gp130, CRP, JAK2, and STAT3. Visualization and quantitation of the fluorescence signal were assessed with the Tissue-FAXS system and Strata-Quest analysi**s** software. Mean intensity to multiply the percentage of positive cells to represent the protein expression levels. The correlation analyses were performed using Spearman regression, and 2-tailed *P* value < 0.05 was considered statistically different.

The correlation between IL-6, IL-6R, gp130, CRP, JAK2, and STAT3 was shown in **Figure 4**. In tumor tissues, there was a moderate correlation between IL-6 and STAT3 (*r* = 0.449, *P* < 0.001), whereas a weak correction between IL-6 and gp130 (*r*= 0.314, *P* = 0.002), as well as JAK2 (*r* = 0.230, *P* = 0.028). Although IL-6 was not correlated with the STAT3 and gp130 in normal tissues (*P* > 0.05), a moderate correlation was observed between IL-6 and JAK2 (*r* = 0.568, *P* = 0.001). IL-6R was weakly corrected with gp130 (*r* = -0.287, *P* = 0.006) and JAK2 (*r* = -0.390, *P* < 0.001) in tumor tissues, and moderately correlated with gp130 (*r* = -0.467, *P* = 0.008) and JAK2 (*r* = -0.555, *P* = 0.001) in normal tissues. Importantly, gp130, a common signaling receptor subunit of the IL-6 family, was strongly correlated with JAK2 in tumor tissues (*r* = 0.755, *P* < 0.001), while a moderate correlation was observed in normal tissues (*r* = 0.447, *P* = 0.012).

### 3.4 The clinical features and prognostic values of IL-6, IL-6R, gp130, CRP, JAK2, and STAT3

**Figure 5:**
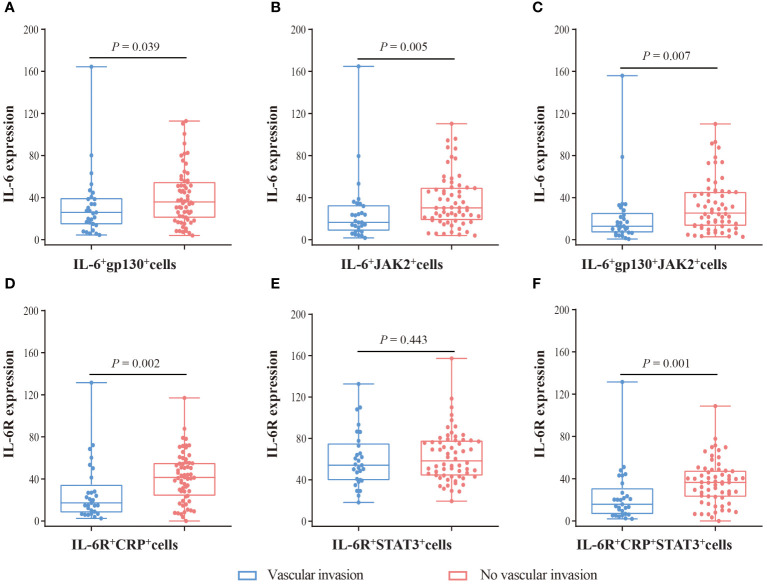
The expression difference of IL-6 and IL-6R in patients with vascular invasion and without vascular invasion. **(A)** The expression of IL-6 in IL-6^+^gp130^+^ cells. **(B)** The expression of IL-6 in IL-6^+^JAK2^+^ cells. **(C)** The expression of IL-6 in IL-6^+^gp130^+^JAK2^+^ cells. **(D)** The expression of IL-6R in IL-6R^+^CRP^+^ cells. **(E)** The expression of IL-6R in IL-6R^+^STAT3^+^ cells. **(F)** The expression of IL-6R in IL-6R^+^CRP ^+^STAT3^+^ cells. Visualization and quantitation of the fluorescence signal were assessed with the Tissue-FAXS system and Strata-Quest analysi**s** software. Mean intensity to multiply the percentage of positive cells to represent the protein expression levels. Mann-Whitney *U* test was used to compare the difference, and a 2-tailed *P* value < 0.05 was considered statistically different.

Furthermore, we evaluated the corrections between clinical characteristics and the expression of IL-6, IL-6R, gp130, CRP, JAK2, and STAT3 (**Table 1**). IL-6 (*P* = 0.030) and gp130 (*P* = 0.031) were associated with male sex. Importantly, CRP (*P* = 0.017), gp130 (*P* = 0.040), and JAK2 (*P* = 0.035) were associated with vascular invasion. Therefore, we further measured the expression difference of IL-6 and IL-6R in double-positive and triple-positive cells in patients with or without vascular invasion (**Figure 5**). Compared with patients without vascular invasion, the expression of IL-6 was usually lower in both IL-6^+^gp130^+^ (*P* = 0.039), IL-6^+^JAK2^+^ (*P* = 0.005), and IL-6^+^gp130^+^JAK2^+^ cells (*P* = 0.007) in patients with vascular invasion (**Figures 5A–C**). The expression of IL-6R was also lower in IL-6R^+^CRP^+^ (*P* = 0.002) and IL-6R^+^CRP^+^STAT3^+^ (*P* = 0.001) cells in patients with vascular invasion when compared to patients without vascular invasion (**Figures 5D–F**). However, none of these gene expression was associated with the OS and DFS (**Supplementary Figures 4**, **5**, all *P* > 0.05), and the same trend was observed using database of TCGA (**Supplementary Figure 6**).

**Table 1:** Comparison of baseline clinicopathological characteristics based on IL-6, IL-6R, CRP, gp130, JAK2, and STAT3 expression in CCA patients.

Characterstics	IL-6			IL-6R			CRP			gp130			JAK2			STAT3		
	high (N=44)	low (N=47)	*P*	high (N=46)	low (N=45)	*P*	high (N=35)	low (N=56)	*P*	high (N=36)	low (N=55)	*P*	high (N=33)	low (N=58)	*P*	high (N=35)	low (N=56)	*P*
Sex																		
Male	18 (40.9%)	30 (63.8%)	**0.029**	25 (54.3%)	23 (51.1%)	0.757	22 (62.9%)	26 (46.4%)	0.127	24 (66.7%)	24 (43.6%)	**0.031**	18 (54.5%)	30 (51.7%)	0.796	18 (51.4%)	30 (53.6%)	0.842
Female	26 (59.1%)	17 (36.2%)		21 (45.7%)	22 (48.9%)		13 (37.1%)	30 (53.6%)		12 (33.3%)	31 (56.4%)		15 (45.5%)	28 (48.3%)		17 (48.6%)	26 (46.4%)	
Tumor differentiation																		
Low	8 (18.2%)	8 (17.4%)	0.207	7 (15.6%)	9 (20.0%)	0.632	6 (17.1%)	10 (18.2%)	0.120	6 (16.7%)	10 (18.5%)	0.863	8 (24.2%)	8 (14.0%)	0.475	7 (20.0%)	9 (16.4%)	0.290
Medium	31 (70.5%)	37 (80.4%)		34 (75.6%)	34 (75.6%)		29 (82.9%)	39 (70.9%)		27 (75.0%)	41 (75.9%)		23 (69.7%)	45 (78.9%)		24 (68.6%)	44 (80.0%)	
High	5 (11.4%)	1 (2.2%)		4 (8.8%)	2 (4.4%)		0 (0.0%)	6 (10.9%)		3 (8.3%)	3 (5.6%)		2 (6.1%)	4 (7.0%)		4 (11.4%)	2 (3.6%)	
TNM																		
I	5 (11.4%)	6 (12.8%)	0.567	6 (13.0%)	5 (11.1%)	0.735	4 (11.4%)	7 (12.5%)	0.458	7 (19.4%)	4 (7.3%)	0.244	6 (18.2%)	5 (8.6%)	0.508	5 (14.3%)	6 (10.7%)	0.771
II	19 (43.2%)	15 (31.9%)		19 (41.3%)	15 (33.3%)		10 (28.6%)	24 (42.9%)		13 (36.1%)	21 (38.2%)		13 (39.4%)	21 (36.2%)		15 (42.9%)	19 (33.9%)	
III	13 (29.5%)	20 (42.6%)		16 (34.8%)	17 (37.8%)		16 (45.7%)	17 (30.4%)		13 (36.1%)	20 (36.4%)		10 (30.3%)	23 (39.7%)		11 (31.4%)	22 (39.3%)	
IV	7 (15.9%)	6 (12.8%)		5 (10.9%)	8 (17.8%)		5 (14.3%)	8 (14.3%)		3 (8.3%)	10 (18.2%)		4 (12.1%)	9 (15.5%)		4 (11.4%)	9 (16.1%)	
Portal lymphnode metastasis																		
Yes	13 (29.5%)	17 (36.2%)	0.502	13 (28.3%)	17 (37.8%)	0.334	14 (40.0%)	16 (28.6%)	0.259	11 (30.6%)	19 (34.5%)	0.692	13 (39.4%)	17 (29.3%)	0.325	11 (31.4%)	19 (33.9%)	0.805
No	31 (70.5%)	30 (63.8%)		33 (71.7%)	28 (62.2%)		21 (60.0%)	40 (71.4%)		25 (69.4%)	36 (65.5%)		20 (60.6%)	41 (70.7%)		24 (68.6%)	37 (66.1%)	
Distant lymphnode metastasis																		
Yes	5 (11.4%)	5 (10.6%)	1	2 (4.3%)	8 (17.8%)	0.087	2 (5.7%)	8 (14.3%)	0.354	5 (13.9%)	5 (9.1%)	0.709	5 (15.2%)	5 (8.6%)	0.542	2 (5.7%)	8 (14.3%)	0.354
No	39 (88.6%)	42 (89.4%)		44 (95.7%)	37 (82.2%)		33 (94.3%)	48 (85.7%)		31 (86.1%)	50 (90.9%)		28 (84.8%)	53 (91.4%)		33 (94.3%)	48 (85.7%)	
Vascular invasion																		
Yes	13 (29.5%)	16 (34.0%)	0.645	14 (30.4%)	15 (33.3%)	0.767	6 (17.1%)	23 (41.1%)	**0.017**	7 (19.4%)	22 (40.0%)	**0.040**	6 (18.2%)	23 (39.7%)	**0.035**	7 (20.0%)	22 (39.3%)	0.055
No	31 (70.5%)	31 (66.0%)		32 (69.6%)	30 (66.7%)		29 (82.9%)	33 (58.9%)		29 (80.6%)	33 (60.0%)		27 (81.8%)	35 (60.3%)		28 (80.0%)	34 (60.7%)	

Values in bold denote statistical significance.

### 3.5 Single-cell transcriptome

**Figure 6:**
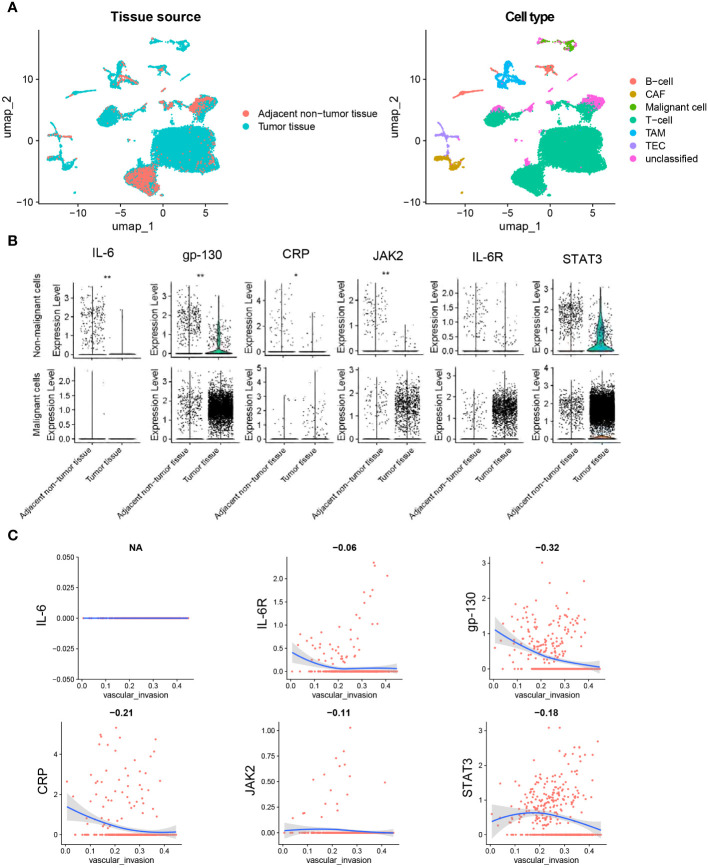
Single-cell analysis of IL-6, IL-6R, gp130, CRP, JAK2, and STAT3 in CCA. **(A)** The UMAP plots of tissue cells from 9 CCA samples. **(B)** Pseudo-bulk expression analysis of genes between tumor tissues and adjacent normal tissues. **(C)** Correlation analysis between gene expression and ssGSEA score of vascular invasion in cells of tumor tissues. The dataset was obtained from Gene Expression Omnibus (GEO, accession id: GSE189903). Wilcoxon test for B.*, P < 0.05. **, P < 0.01. Spearman regression was used for C, and a 2-tailed *P* value < 0.05 was considered statistically different. CAF, cancer-associated fibroblasts; TEC, tumor-associated endothelial cells; TAM, tumor-associated macrophage.

Single-cell solution, single-cell data of CCA tissues (n=9) were analyzed to further identify the role of IL-6 signal genes expression in CCA (**Figure 6A**). In non-malignant cells, the expression of IL-6 (*P* = 0.005), gp-130 (*P* < 0.001), CRP (*P* = 0.020), and JAK2 (*P* = 0.002) was lower in tumor tissues compared with normal tissues (**Figure 6B**), while expression of IL6R and STAT3 showed no significant difference in malignant cells (*P* > 0.05, **Figure 6B**). CRP and gp-130 was found to be negatively correlated with vascular invasion in malignant cells (*P* < 0.001, **Figure 6C**).

## 4 Discussion

As a pleiotropic cytokine, IL-6 is primarily produced in response to stress and infection in the presence of inflammatory stimuli. It was reported to play an important role in the tumorigenesis and progression of CCA. Aberrant expression of IL-6 could promote tumor cell growth and metastasis by activating the IL-6/STAT3 signaling pathway. Our study examined the expression and correlation of IL-6, IL-6R, gp130, JAK2, STAT3, and CRP in CCA tissue and normal tissue. We found that the expression of IL-6 was lower, while the expression of STAT3 was higher in tumor tissues using mIF. Especially in SA region, we observed a significant lower expression of IL-6, CRP, gp-130, and JAK2 in single-positive, double-positive or triple-positive cells. Single-cell transcriptome analysis also showed the expression of IL-6, gp-130, CRP and JAK2 was lower in tumor tissues compared with normal tissues. Importantly, gp130, a common signaling receptor subunit of the IL-6 family (also known as IL-6Rβ), was moderately associated with the expression of JAK2 in normal tissue, whereas it was strongly correlated with JAK2 in tumor tissues. Furthermore, we evaluated the clinical characteristics and prognostic significance of these gene expressions. Although none of the gene expressions were associated with OS and DFS, our study revealed an inverse association between IL-6, IL-6R, CRP, gp130, and JAK2 with vascular invasion, leading to a poor prognosis in patients with CCA (OS: aHR = 1.885, 95% CI = 1.118 - 3.179, *P* = 0.017; DFS: aHR = 2.172, 95% CI = 1.252 - 3.768, *P* = 0.006).

Serum IL-6 concentration is elevated in patients with CCA compared to healthy controls and is positively correlated with tumor burden (21). Elevated serum IL-6 levels could promote the proliferation of CCA cells in both human and murine models (17, 22). Surprisingly, we found that the positive rate of IL-6 was comparable between CCA tumor tissues and normal tissues, while the mean intensity was lower in the CCA tumor tissues (data not show). Another study that also observed an inverse relationship between IL-6 expression and cell proliferation while showing a positive correlation with differentiation in CCA (23). In SA region, we found both the positive rate and mean intensity of most IL-6 family was lower in tumor tissues when compared with normal tissues (data not show), indicating that the lower expression of IL-6 family mainly due to the cells of tumor microenvironment. Results from single-cell transcriptome analysis also confirmed this finding. Tumor microenvironment plays a key role in the progression and invasiveness of CCA (24). CCA was a “cold tumor” characterized by reduced effector immune cell infiltration (25). The downregulated IL-6 pathway may indicate an immunosuppressive microenvironment, leading to the immune escape of tumor cells. In CCA tumor tissues, the expression of IL-6 was positively correlated with gp130, JAK2, and STAT3. Meanwhile, gp130 was strongly correlated with JAK2. However, in normal tissues, IL-6 was not correlated with gp130. These results also support that IL-6/JAK2/STAT3 signaling pathway might play a critical role in the pathogeneses of CCA. In addition, IL-6Rα was found to be downregulated in another bile duct cancer and gallbladder cancer, and low IL-6Rα expression correlated with poor OS (18).

Vascular invasion is inversely correlated with prognosis in patients with CCA. The present study showed that the IL-6 pathway (including IL-6, IL-6R, gp130, and JAK2) was inversely correlated with vascular invasion. Compared to intrahepatic cholangiocarcinoma patients without vascular invasion, those with vascular invasion also exhibited reduced levels of plasma IL-6 (26). As a pleiotropic cytokine, IL-6 signaling plays a complex role in inflammation. There are two faces of IL-6 in tumor microenvironment: on one hand, IL-6 has been widely described as anti-inflammatory in some settings; on the other hand, it also plays critical roles in promoting inflammation and immunity (27). IL-6 pathway is also reported as a key player in the mobilization of anti-tumor T cell responses (24). For example, IL-6 promotes the production of IL-10 by T cells, which in turn restricts many inflammatory processes (28). Otherwise, IL-6 plays a crucial role in the proliferation, survival, and commitment of T cells, and it also modulates their effector cytokine production (28). However, the precise mechanisms involved remain unclear and require further investigation.

Furthermore, we found that CRP was negatively correlated with vascular invasion. Other studies have reported that the expression of CRP in tumor tissues is associated with the mass-forming gross type, absence of perineural invasion, and a better prognosis (29). Thus, CRP could be a promising diagnostic and prognostic immunohistochemical marker for CCA. However, contrasting results were also found in the relationship between serum CRP levels and prognosis. Elevated preoperative serum CRP levels were correlated with poor clinical outcomes (30).

However, there are several limitations in our study. Firstly, we were unable to analyze the correlation of protein expression between serum levels and tumor tissues because preoperative serum levels are not routinely checked in our patients. Secondly, the samples in the present study were obtained from a single center, and it is necessary to have samples from multiple centers for validation. Third, ten patients missed follow-up when analyzing the OS, which could introduce bias. Finally, the underlying molecular mechanisms of the IL-6 pathway in CCA warrant further study.

In general, our study investigated the expression, clinical features, and correlation of IL-6, IL-6R, CRP, gp130, and JAK2 in CCA tissue. The IL-6 signaling pathway was found to be inversely associated with the carcinogenesis and development of CCA. The downregulated IL-6 pathway in tumor tissues may indicate an immunosuppressive microenvironment. Findings from this study suggest a potential prognostic value of the IL-6 pathway for CCA, and the underlying mechanism requires further investigation.

## Data availability statement

The original contributions presented in the study are included in the article/**Supplementary Material**. Further inquiries can be directed to the corresponding authors.

## Ethics statement

The studies involving humans were approved by ethics committee of the Shanghai Outdo Company. The studies were conducted in accordance with the local legislation and institutional requirements. Written informed consent for participation in this study was provided by the participants’ legal guardians/next of kin.

## Author contributions

DG: Writing – original draft, Validation, Methodology, Funding acquisition, Formal analysis, Data curation. XZ: Writing – original draft, Validation, Resources, Data curation. JS: Formal analysis, Writing – review & editing. JX: Writing – review & editing, Validation, Data curation. LZ: Writing – review & editing, Data curation. GD: Writing – review & editing, Validation, Project administration. DL: Writing – review & editing, Validation, Resources, Project administration.
