# Supplementary material for: Expression and clinical significance of interleukin-6 pathway in cholangiocarcinoma
Source: Front Immunol. 2024 May 31;15:1374967. doi: 10.3389/fimmu.2024.1374967 (PMC11176422; doi:10.3389/fimmu.2024.1374967)
Supplement: Supplementary file 1 [file DataSheet_1.docx]

Supplementary Material

# Supplementary Figures and Tables

## Supplementary Figures

**
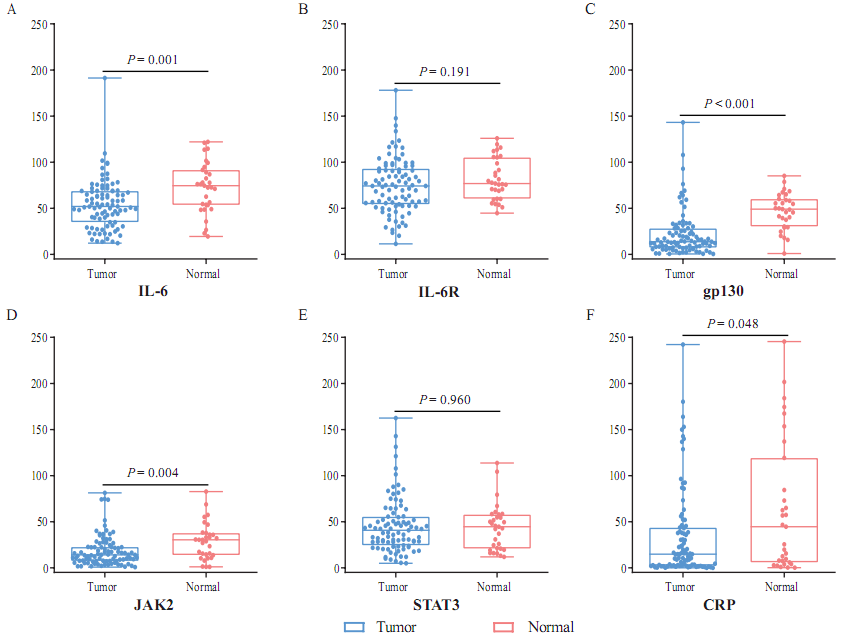
**

**Supplementary Fig. 1.** **The expression difference of IL6(A), IL6R(B), gp130(C), JAK2(D), STAT3(E), and CRP(F) between tumor (blue) and normal tissue (red) in SA region.** Visualization and quantitation of the fluorescence signal were assessed with the Tissue-FAXS system and Strata-Quest analysis software. Mean intensity to multiply the percentage of positive cells to represent the protein expression levels. Mann-Whitney U test was used to compare the difference, and a 2-tailed P value < 0.05 was considered statistically different.


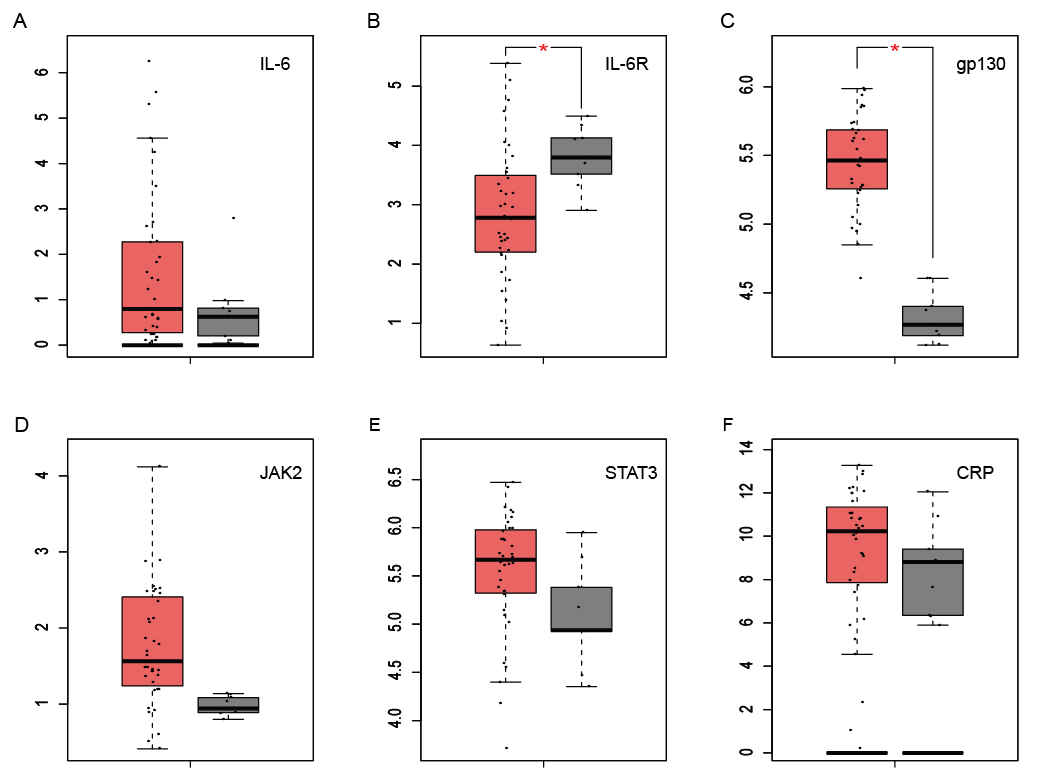


**Supplementary Fig. 2. The expression difference of IL6(A), IL6R(B), gp130(C), JAK2(D), STAT3(E), and CRP(F) between tumor (red) and normal tissue (grey) by GEPIA.** The data source came from TCGA, which included 36 tumor tissue samples and 9 normal tissue samples. (* represent *P* < 0.05).


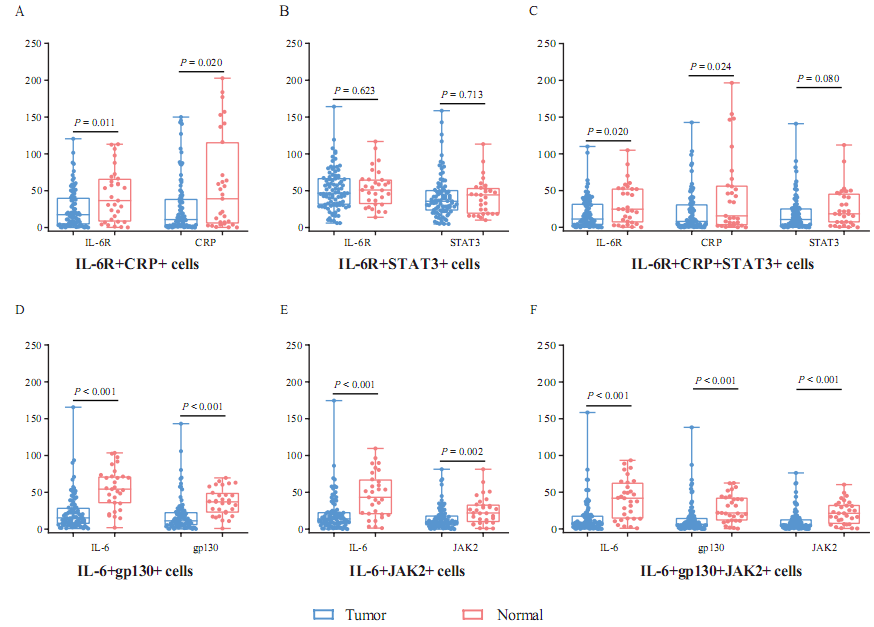


**Supplementary Fig. 3.** **The expression difference of IL-6, IL-6R, gp130, JAK2, STAT3, and CRP in double-positive and triple-positive cells of SA region**. (A) The expression of IL-6R and CRP in IL-6R+CRP+ cells. (B) The expression of IL-6R and STAT3 in IL-6R+STAT3+ cells. (C) The expression of IL-6R, CRP, and STAT3 in IL-6R+CRP+STAT3+ cells. (D) The expression of IL-6 and gp130 in IL-6+gp130+ cells. (E) The expression of IL-6 and JAK2 in IL-6+JAK2+ cells. (F) The expression of IL-6, gp130, and JAK2 in IL-6+gp130+JAK2+ cells. Visualization and quantitation of the fluorescence signal were assessed with the Tissue-FAXS system and Strata-Quest analysis software. Mean intensity to multiply the percentage of positive cells to represent the protein expression levels. Mann-Whitney U test was used to compare the difference, and a 2-tailed P value < 0.05 was considered statistically different.

**
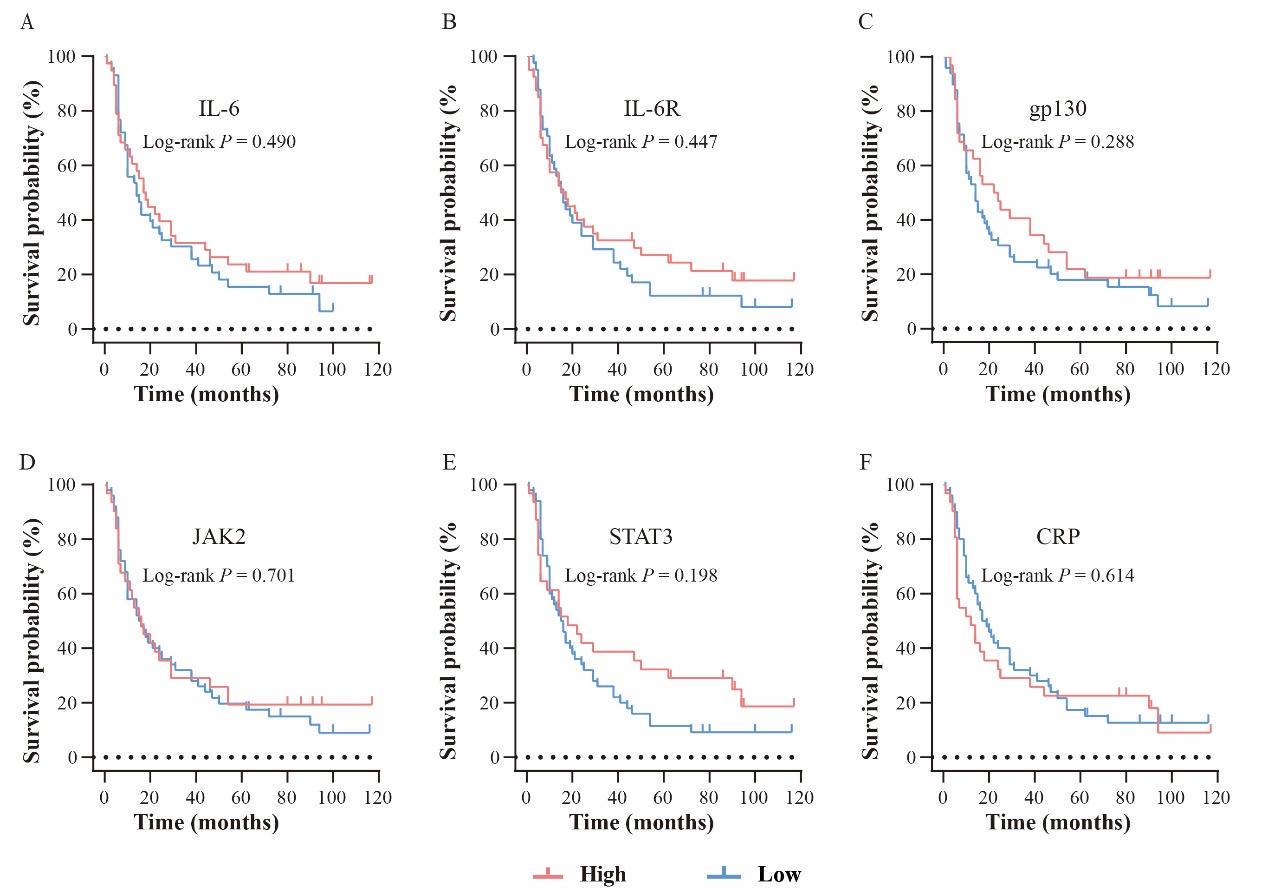
**

**Supplementary Fig. 4. Kaplan–Meier analysis of the expression of IL6(A), IL6R(B), gp130(C), JAK2(D), STAT3(E), and CRP(F) in cholangiocarcinoma and OS.** Log-rank test was used for group comparisons, and a 2-tailed P value < 0.05 was considered statistically different.

**
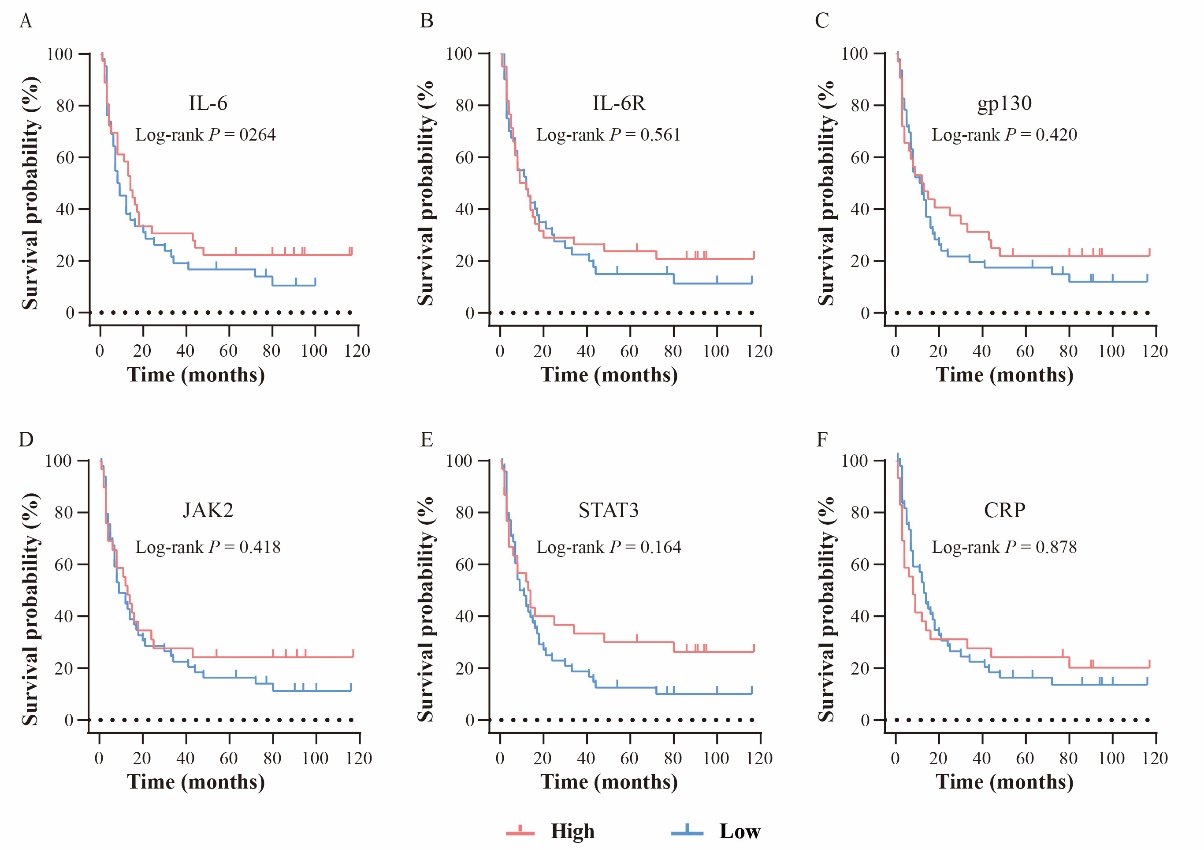
**

**Supplementary Fig. 5. Kaplan–Meier analysis of the expression of IL6(A), IL6R(B), gp130(C), JAK2(D), STAT3(E), and CRP(F) in cholangiocarcinoma and DFS.** Log-rank test was used for group comparisons, and a 2-tailed P value < 0.05 was considered statistically different.


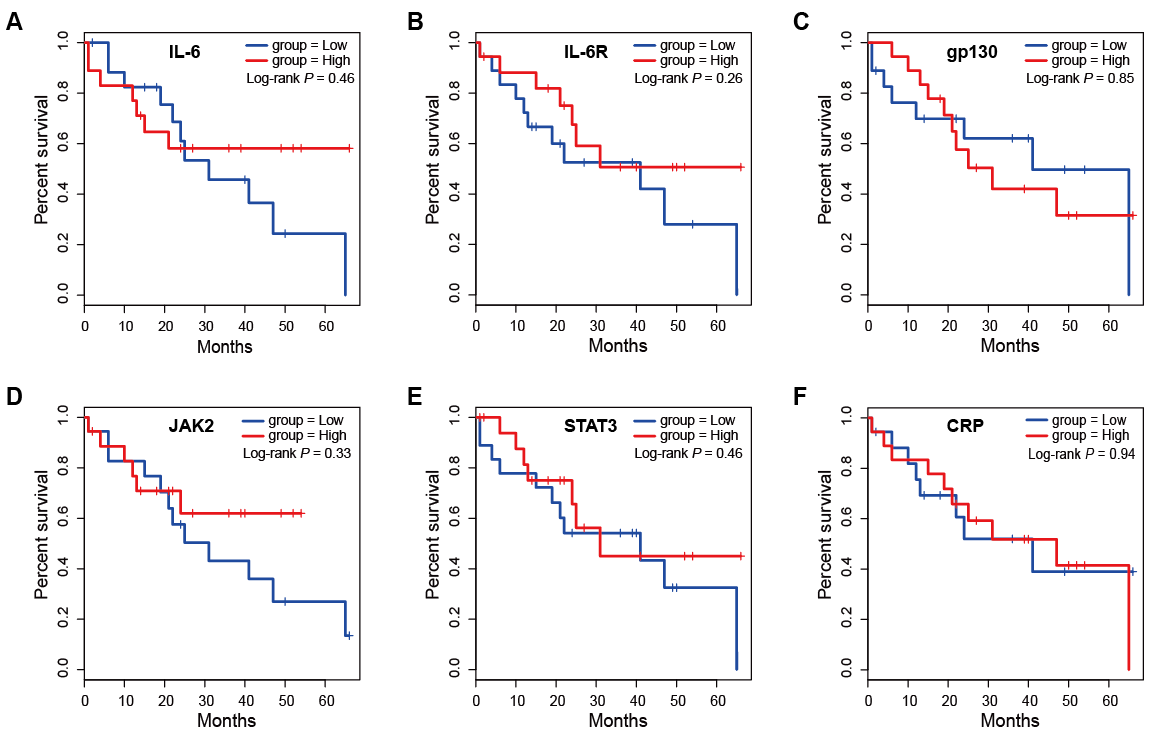


**Supplementary Fig. 6. Kaplan–Meier analysis of the expression of IL6(A), IL6R(B), gp130(C), JAK2(D), STAT3(E), and CRP(F) in cholangiocarcinoma and OS in TCGA databases.**

## Supplementary Tables

**Supplementary Table 1 The description of the tissue microarray.**

| **Characteristics** | **Values** |
| --- | --- |
| **Sample size** | 91 |
| **Male sex** | 48 (52.7%) |
| **Age** | 56.20±9.83 |
| **White blood cell** | 6.69 (5.40 - 8.25) |
| **Neutrophils** | 67.5 (59.7 - 74.0) |
| **Lymphocyte** | 21.3 (14.7 - 26.4) |
| **Hemoglobin** | 123 (107 - 132) |
| **Platelet** | 219 (181 - 280) |
| **CA12_5** | 19.30 (9.33 - 31.65) |
| **CA19_9** | 169.64 (22.68 - 362.98) |
| **CEA** | 3.05 (1.82 - 4.90) |
| **CA24_2** | 22.2 (5.7 - 102.0) |
| **AFP** | 4.12 (2.84 - 8.06) |
| **HBsAg positive** | 8 (8.8%) |
| **TNM stage** |  |
| I | 11 (12.1%) |
| II | 34 (37.4%) |
| III | 33 (36.3%) |
| IV | 13 (14.3%) |
| **Tumor differentiation** |  |
| Low | 16 (17.6%) |
| Medium | 68 (74.7%) |
| High | 6 (6.6%) |
| Mucinous cystadenocarcinoma | 1 (1.1%) |
| **Portal lymphnode metastasis** |  |
| Yes | 30 (33.0%) |
| No | 61 (67.0%) |
| **Distant lymphnode metastasis** |  |
| Yes | 10 (11.0%) |
| No | 81 (89.0%) |
| **Vascular invasion** |  |
| Yes | 29 (31.9%) |
| No | 62 (68.1%) |
| **Perineural invasion** |  |
| Yes | 11 (12.1%) |
| No | 73 (80.2%) |
| Unknown | 7 (7.7%) |
| **Recurrence** |  |
| Yes | 65 (71.4%) |
| No | 15 (16.5%) |
| Missing | 11 (12.1%) |
| Disease-free survival (months) | 11.5 (4.0 - 33.75) |
| **Overall survival** |  |
| Dead | 69 (75.8%) |
| Alive | 12 (13.2%) |
| Missing | 10 (11.0%) |
| Overall survival (months) | 16 (6 - 46) |
