# Supplementary material for: Expression and clinical significance of interleukin-6 pathway in cholangiocarcinoma
Source: Front Immunol. 2024 May 31;15:1374967. doi: 10.3389/fimmu.2024.1374967 (PMC11176422; doi:10.3389/fimmu.2024.1374967)
Supplement: Supplementary file 2 [file DataSheet_2.pdf]

```
 GET
 FILE='D:
\D\project\IL6 and BTC\manuscript\frontiers in immunology\Expression and prognosis
.sav'.
DATASET NAME 1 WINDOW=FRONT.
GET
 FILE='D:
\D\project\IL6 and BTC\manuscript\frontiers in immunology\expression difference.
sav'.
DATASET NAME 2 WINDOW=FRONT.
DATASET ACTIVATE 1.
FREQUENCIES VARIABLES=age
 /FORMAT=NOTABLE
 /STATISTICS=STDDEV MEAN
 /ORDER=ANALYSIS.
```

Frequencies

Notes

Output Created		25-JAN-2024 08:29:10
Comments		
Input	Data	D:\D\project\IL6 and BTC\manuscript\frontiers in immunology\Expression and prognosis.sav
	Active Dataset	1
	Filter	<none>
	Weight	<none>
	Split File	<none>
	N of Rows in Working Data File	91
Missing Value Handling	Definition of Missing	User-defined missing values are treated as missing.
	Cases Used	Statistics are based on all cases with valid data.
Syntax		FREQUENCIES VARIABLES=age /FORMAT=NOTABLE /STATISTICS=STDDEV MEAN /ORDER=ANALYSIS.
Resources	Processor Time	00:00:00.00
	Elapsed Time	00:00:00.01

[1] D:\D\project\IL6 and BTC\manuscript\frontiers in immunology\Expression and prognosis.sav

Statistics

age

N	Valid	91
	Missing	0
Mean		56.20
Std. Deviation		9.828

```
FREQUENCIES VARIABLES=wbc Neutrophils lymphocyte hemoglobin plt CA12_5 CA19_9
CEA CA24_2 AFP
  Survival_month DFS_month
 /FORMAT=NOTABLE
 /NTILES=4
 /ORDER=ANALYSIS.
```

Frequencies

Notes

Output Created		25-JAN-2024 08:30:53
Comments		
Input	Data	D:\D\project\IL6 and BTC\manuscript\frontiers in immunology\Expression and prognosis.sav
	Active Dataset	1
	Filter	<none>
	Weight	<none>
	Split File	<none>
	N of Rows in Working Data File	91
Missing Value Handling	Definition of Missing	User-defined missing values are treated as missing.
	Cases Used	Statistics are based on all cases with valid data.
Syntax		FREQUENCIES VARIABLES=wbc Neutrophils lymphocyte hemoglobin plt CA12_5 CA19_9 CEA CA24_2 AFP Survival_month DFS_month /FORMAT=NOTABLE /NTILES=4 /ORDER=ANALYSIS.
Resources	Processor Time	00:00:00.00
	Elapsed Time	00:00:00.02

Statistics

		wbc	Neutrophils	lymphocyte	hemoglobin	plt	CA12_5	CA19_9	CEA	CA24_2	AFP	Survival_month	DFS_month
N	Valid	91	91	91	91	91	89	84	91	91	91	81	80
	Missing	0	0	0	0	0	2	7	0	0	0	10	11
Percentiles	25	5.40	59.70	14.70	107.00	181.00	9.33	22.68	1.82	5.70	2.84	6.00	4.00
	50	6.69	67.50	21.30	123.00	219.00	19.30	169.64	3.05	22.20	4.12	16.00	11.50
	75	8.25	74.00	26.40	132.00	280.00	31.65	362.98	4.90	102.00	8.06	46.00	33.75

```
DATASET ACTIVATE 2.
NPAR TESTS
 /M-W= IL6R CRP STAT3 CK19 GP130 JAK2 IL6 BY group(0 1)
 /MISSING ANALYSIS.
```

NPar Tests

Notes

Output Created		25-JAN-2024 08:32:50
Comments		
Input	Data	D:\D\project\IL6 and BTC\manuscript\frontiers in immunology\expression difference.sav
	Active Dataset	2
	Filter	<none>
	Weight	<none>
	Split File	<none>
	N of Rows in Working Data File	122
Missing Value Handling	Definition of Missing	User-defined missing values are treated as missing.
	Cases Used	Statistics for each test are based on all cases with valid data for the variable(s) used in that test.
Syntax		NPAR TESTS<br>/M-W= IL6R CRP STAT3 CK19 GP130 JAK2 IL6 BY group(0 1)<br>/MISSING ANALYSIS.
Resources	Processor Time	00:00:00.00
	Elapsed Time	00:00:00.02
	Number of Cases Allowed[a]	241979

a. Based on availability of workspace memory.

```
[2] D:\D\project\IL6 and BTC\manuscript\frontiers in immunology\expression difference.sav
```

Mann-Whitney Test

Ranks

	group	N	Mean Rank	Sum of Ranks
IL6R	0	91	60.45	5500.50
	1	31	64.60	2002.50
	Total	122		
CRP	0	91	60.40	5496.00
	1	31	64.74	2007.00
	Total	122		
STAT3	0	91	65.33	5945.00
	1	31	50.26	1558.00
	Total	122		
CK19	0	91	74.18	6750.00
	1	31	24.29	753.00
	Total	122		
GP130	0	91	58.26	5302.00
	1	31	71.00	2201.00
	Total	122		
JAK2	0	91	62.14	5655.00
	1	31	59.61	1848.00
	Total	122		
IL6	0	91	57.82	5262.00
	1	31	72.29	2241.00
	Total	122		

Test Statistics[a]

	IL6R	CRP	STAT3	CK19	GP130	JAK2	IL6
Mann-Whitney U	1314.500	1310.000	1062.000	257.000	1116.000	1352.000	1076.000
Wilcoxon W	5500.500	5496.000	1558.000	753.000	5302.000	1848.000	5262.000
Z	-.565	-.591	-2.049	-6.784	-1.732	-.344	-1.967
Asymp. Sig. (2-tailed)	.572	.555	.040	.000	.083	.731	.049

a. Grouping Variable: group

```
NPAR TESTS
 /M-W= IL6R_CRP_IL6R IL6R_CRP_CRP IL6R_STAT3_IL6R IL6R_STAT3_STAT3 IL6R_CRP_S
TAT3_IL6R
  IL6R_CRP_STAT3_CRP IL6R_CRP_STAT3_STAT3 gp130_IL6_gp130 gp130_IL6_IL6 JAK2
_IL6_JAK2 JAK2_IL6_IL6
  gp130_JAK2_IL6_gp130 gp130_JAK2_IL6_JAK2 gp130_JAK2_IL6_IL6 BY group(0 1)
 /MISSING ANALYSIS.
```

NPar Tests

Notes

Output Created		25-JAN-2024 08:33:48
Comments		
Input	Data	D:\D\project\IL6 and BTC\manuscript\frontiers in immunology\expression difference.sav
	Active Dataset	2
	Filter	<none>
	Weight	<none>
	Split File	<none>
	N of Rows in Working Data File	122
Missing Value Handling	Definition of Missing	User-defined missing values are treated as missing.
	Cases Used	Statistics for each test are based on all cases with valid data for the variable(s) used in that test.
Syntax		NPAR TESTS<br>/M-W= IL6R_CRP_IL6R IL6R_CRP_CRP IL6R_STAT3_IL6R IL6R_STAT3_STAT3 IL6R_CRP_STAT3_IL6R<br>IL6R_CRP_STAT3_CRP IL6R_CRP_STAT3_STAT3 gp130_IL6_gp130 gp130_IL6_IL6 JAK2_IL6_JAK2 JAK2_IL6_IL6<br>gp130_JAK2_IL6_gp130 gp130_JAK2_IL6_JAK2 gp130_JAK2_IL6_IL6 BY group(0 1)<br>/MISSING ANALYSIS.
Resources	Processor Time	00:00:00.02
	Elapsed Time	00:00:00.01
	Number of Cases Allowed[a]	157286

a. Based on availability of workspace memory.

Mann-Whitney Test

Ranks

	group	N	Mean Rank	Sum of Ranks
IL6R_CRP_IL6R	0	91	60.35	5491.50
	1	31	64.89	2011.50
	Total	122		
IL6R_CRP_CRP	0	91	59.92	5453.00
	1	31	66.13	2050.00
	Total	122		
IL6R_STAT3_IL6R	0	91	64.12	5835.00
	1	31	53.81	1668.00
	Total	122		
IL6R_STAT3_STAT3	0	91	64.96	5911.00
	1	31	51.35	1592.00
	Total	122		
IL6R_CRP_STAT3_IL6R	0	91	61.63	5608.00
	1	31	61.13	1895.00
	Total	122		
IL6R_CRP_STAT3_CRP	0	91	60.99	5550.00
	1	31	63.00	1953.00
	Total	122		
IL6R_CRP_STAT3_STAT3	0	91	63.62	5789.00
	1	31	55.29	1714.00
	Total	122		
gp130_IL6_gp130	0	91	59.35	5401.00
	1	31	67.81	2102.00
	Total	122		
gp130_IL6_IL6	0	91	55.25	5028.00
	1	31	79.84	2475.00
	Total	122		
JAK2_IL6_JAK2	0	91	61.01	5552.00
	1	31	62.94	1951.00
	Total	122		
JAK2_IL6_IL6	0	91	56.91	5179.00
	1	31	74.97	2324.00
	Total	122		
gp130_JAK2_IL6_gp130	0	91	60.75	5528.00
	1	31	63.71	1975.00
	Total	122		
gp130_JAK2_IL6_JAK2	0	91	60.56	5511.00
	1	31	64.26	1992.00
	Total	122		
gp130_JAK2_IL6_IL6	0	91	56.73	5162.00
	1	31	75.52	2341.00
	Total	122		

Test Statistics[a]

	IL6R_CRP_IL6R	IL6R_CRP_CRP	IL6R_STAT3_IL6R	IL6R_STAT3_STAT3	IL6R_CRP_STAT3_IL6R	IL6R_CRP_STAT3_CRP	IL6R_CRP_STAT3_STAT3	gp130_IL6_gp130	gp130_IL6_IL6	JAK2_IL6_JAK2	JAK2_IL6_IL6	gp130_JAK2_IL6_gp130	gp130_JAK2_IL6_JAK2	gp130_JAK2_IL6_IL6
Mann-Whitney U	1305.500	1267.000	1172.000	1096.000	1399.000	1364.000	1218.000	1215.000	842.000	1366.000	993.000	1342.000	1325.000	976.000
Wilcoxon W	5491.500	5453.000	1668.000	1592.000	1895.000	5550.000	1714.000	5401.000	5028.000	5552.000	5179.000	5528.000	5511.000	5162.000
Z	-.617	-.844	-1.403	-1.850	-.068	-.273	-1.132	-1.150	-3.343	-.262	-2.455	-.403	-.503	-2.555
Asymp. Sig. (2-tailed)	.537	.399	.161	.064	.946	.785	.258	.250	.001	.794	.014	.687	.615	.011

a. Grouping Variable: group

```
SORT CASES BY group.
SPLIT FILE LAYERED BY group.
CORRELATIONS
  /VARIABLES=IL6R CRP STAT3 CK19 GP130 JAK2 IL6
  /PRINT=TWOTAIL NOSIG
  /MISSING=PAIRWISE.
```

Correlations

Notes

Output Created		25-JAN-2024 08:35:25
Comments		
Input	Data	D:\D\project\IL6 and BTC\manuscript\frontiers in immunology\expression difference.sav
	Active Dataset	2
	Filter	<none>
	Weight	<none>
	Split File	group
	N of Rows in Working Data File	122
Missing Value Handling	Definition of Missing	User-defined missing values are treated as missing.
	Cases Used	Statistics for each pair of variables are based on all the cases with valid data for that pair.
Syntax		CORRELATIONS<br>/VARIABLES=IL6R CRP STAT3 CK19 GP130 JAK2 IL6<br>/PRINT=TWOTAIL NOSIG<br>/MISSING=PAIRWISE.
Resources	Processor Time	00:00:00.00
	Elapsed Time	00:00:00.01

Correlations

group			IL6R	CRP	STAT3	CK19	GP130	JAK2	IL6
0	IL6R	Pearson Correlation	1	-.165	.209*	-.067	-.208*	-.302**	.122
		Sig. (2-tailed)		.118	.047	.531	.048	.004	.247
		N	91	91	91	91	91	91	91
	CRP	Pearson Correlation	-.165	1	.169	.199	.289**	.367**	.165
		Sig. (2-tailed)	.118		.110	.058	.005	.000	.119
		N	91	91	91	91	91	91	91
	STAT3	Pearson Correlation	.209*	.169	1	.261*	.291**	.249*	.519**
		Sig. (2-tailed)	.047	.110		.012	.005	.017	.000
		N	91	91	91	91	91	91	91
	CK19	Pearson Correlation	-.067	.199	.261*	1	.482**	.427**	.114
		Sig. (2-tailed)	.531	.058	.012		.000	.000	.282
		N	91	91	91	91	91	91	91
	GP130	Pearson Correlation	-.208*	.289**	.291**	.482**	1	.839**	.444**
		Sig. (2-tailed)	.048	.005	.005	.000		.000	.000
		N	91	91	91	91	91	91	91
	JAK2	Pearson Correlation	-.302**	.367**	.249*	.427**	.839**	1	.364**
		Sig. (2-tailed)	.004	.000	.017	.000	.000		.000
		N	91	91	91	91	91	91	91
	IL6	Pearson Correlation	.122	.165	.519**	.114	.444**	.364**	1
		Sig. (2-tailed)	.247	.119	.000	.282	.000	.000	
		N	91	91	91	91	91	91	91
1	IL6R	Pearson Correlation	1	-.064	.265	.002	-.455*	-.508**	-.240
		Sig. (2-tailed)		.732	.150	.990	.010	.004	.193
		N	31	31	31	31	31	31	31
	CRP	Pearson Correlation	-.064	1	.360*	.272	-.205	.504**	.024
		Sig. (2-tailed)	.732		.047	.139	.270	.004	.900
		N	31	31	31	31	31	31	31
	STAT3	Pearson Correlation	.265	.360*	1	.356*	-.439*	.276	.265
		Sig. (2-tailed)	.150	.047		.049	.014	.133	.149
		N	31	31	31	31	31	31	31
	CK19	Pearson Correlation	.002	.272	.356*	1	-.469**	-.093	-.057
		Sig. (2-tailed)	.990	.139	.049		.008	.619	.759
		N	31	31	31	31	31	31	31
	GP130	Pearson Correlation	-.455*	-.205	-.439*	-.469**	1	.311	.309
		Sig. (2-tailed)	.010	.270	.014	.008		.089	.091
		N	31	31	31	31	31	31	31
	JAK2	Pearson Correlation	-.508**	.504**	.276	-.093	.311	1	.564**
		Sig. (2-tailed)	.004	.004	.133	.619	.089		.001
		N	31	31	31	31	31	31	31
	IL6	Pearson Correlation	-.240	.024	.265	-.057	.309	.564**	1
		Sig. (2-tailed)	.193	.900	.149	.759	.091	.001	
		N	31	31	31	31	31	31	31

*. Correlation is significant at the 0.05 level (2-tailed).

**. Correlation is significant at the 0.01 level (2-tailed).

```
NONPAR CORR
/VARIABLES= IL6R CRP STAT3 CK19 GP130 JAK2 IL6
```

```
/PRINT=SPEARMAN TWOTAIL NOSIG
/MISSING=PAIRWISE.
```

Nonparametric Correlations

Notes

Output Created		25-JAN-2024 08:35:25
Comments		
Input	Data	D:\D\project\IL6 and BTC\manuscript\frontiers in immunology\expression difference.sav
	Active Dataset	2
	Filter	<none>
	Weight	<none>
	Split File	group
	N of Rows in Working Data File	122
Missing Value Handling	Definition of Missing	User-defined missing values are treated as missing.
	Cases Used	Statistics for each pair of variables are based on all the cases with valid data for that pair.
Syntax		NONPAR CORR /VARIABLES=IL6R CRP STAT3 CK19 GP130 JAK2 IL6 /PRINT=SPEARMAN TWOTAIL NOSIG /MISSING=PAIRWISE.
Resources	Processor Time	00:00:00.00
	Elapsed Time	00:00:00.01
	Number of Cases Allowed	314572 cases[a]

a. Based on availability of workspace memory

Correlations

group				IL6R	CRP	STAT3	CK19	GP130	JAK2	IL6
Spearman's rho	0	IL6R	Correlation Coefficient	1.000	-.134	.214*	-.122	-.287**	-.390**	.088
			Sig. (2-tailed)	.	.204	.041	.248	.006	.000	.405
			N	91	91	91	91	91	91	91
		CRP	Correlation Coefficient	-.134	1.000	.186	.294**	.302**	.357**	.085
			Sig. (2-tailed)	.204	.	.078	.005	.004	.001	.421
			N	91	91	91	91	91	91	91
		STAT3	Correlation Coefficient	.214*	.186	1.000	.234*	.122	.144	.449**
			Sig. (2-tailed)	.041	.078	.	.026	.251	.174	.000
			N	91	91	91	91	91	91	91
		CK19	Correlation Coefficient	-.122	.294**	.234*	1.000	.467**	.457**	.027
			Sig. (2-tailed)	.248	.005	.026	.	.000	.000	.799
			N	91	91	91	91	91	91	91
		GP130	Correlation Coefficient	-.287**	.302**	.122	.467**	1.000	.755**	.314**
			Sig. (2-tailed)	.006	.004	.251	.000	.	.000	.002
			N	91	91	91	91	91	91	91
		JAK2	Correlation Coefficient	-.390**	.357**	.144	.457**	.755**	1.000	.248*
			Sig. (2-tailed)	.000	.001	.174	.000	.000	.	.018
			N	91	91	91	91	91	91	91
		IL6	Correlation Coefficient	.088	.085	.449**	.027	.314**	.248*	1.000
			Sig. (2-tailed)	.405	.421	.000	.799	.002	.018	.
			N	91	91	91	91	91	91	91
	1	IL6R	Correlation Coefficient	1.000	-.051	.185	-.060	-.467**	-.555**	-.261
			Sig. (2-tailed)	.	.786	.320	.750	.008	.001	.156
			N	31	31	31	31	31	31	31
		CRP	Correlation Coefficient	-.051	1.000	.281	.033	-.250	.274	-.104
			Sig. (2-tailed)	.786	.	.126	.862	.175	.136	.578
			N	31	31	31	31	31	31	31
		STAT3	Correlation Coefficient	.185	.281	1.000	.458**	-.299	.133	.204
			Sig. (2-tailed)	.320	.126	.	.010	.102	.477	.272
			N	31	31	31	31	31	31	31
		CK19	Correlation Coefficient	-.060	.033	.458**	1.000	-.389*	-.133	.142
			Sig. (2-tailed)	.750	.862	.010	.	.031	.474	.445
			N	31	31	31	31	31	31	31
		GP130	Correlation Coefficient	-.467**	-.250	-.299	-.389*	1.000	.447*	.345
			Sig. (2-tailed)	.008	.175	.102	.031	.	.012	.057
			N	31	31	31	31	31	31	31
		JAK2	Correlation Coefficient	-.555**	.274	.133	-.133	.447*	1.000	.568**
			Sig. (2-tailed)	.001	.136	.477	.474	.012	.	.001
			N	31	31	31	31	31	31	31
		IL6	Correlation Coefficient	-.261	-.104	.204	.142	.345	.568**	1.000
			Sig. (2-tailed)	.156	.578	.272	.445	.057	.001	.
			N	31	31	31	31	31	31	31

*. Correlation is significant at the 0.05 level (2-tailed).

**. Correlation is significant at the 0.01 level (2-tailed).

```
SPLIT FILE OFF.
DATASET ACTIVATE 1.
CROSSTABS
  /TABLES= sex BY IL6R
  /FORMAT= AVALUE TABLES
  /STATISTICS = CHISQ
  /CELLS= COUNT ROW
  /COUNT ROUND CELL.
```

Crosstabs

Notes

Output Created		25-JAN-2024 08:39:30
Comments		
Input	Data	D:\D\project\IL6 and BTC\manuscript\frontiers in immunology\Expression and prognosis.sav
	Active Dataset	1
	Filter	<none>
	Weight	<none>
	Split File	<none>
	N of Rows in Working Data File	91
Missing Value Handling	Definition of Missing	User-defined missing values are treated as missing.
	Cases Used	Statistics for each table are based on all the cases with valid data in the specified range(s) for all variables in each table.
Syntax		CROSSTABS<br>/TABLES=sex BY IL6R<br>/FORMAT=AVALUE TABLES<br>/STATISTICS=CHISQ<br>/CELLS=COUNT ROW<br>/COUNT ROUND CELL.
Resources	Processor Time	00:00:00.02
	Elapsed Time	00:00:00.41
	Dimensions Requested	2
	Cells Available	524245

[1] D:\D\project\IL6 and BTC\manuscript\frontiers in immunology\Expression and prognosis.sav

```
CROSSTABS
/TABLES= sex BY IL6R CRP STAT3 CK19 GP130 JAK2 IL6
/FORMAT=AVALUE TABLES
/STATISTICS = CHISQ
/CELLS= COUNT COLUMN
/COUNT ROUND CELL.
```

Crosstabs

Notes

Output Created		25-JAN-2024 08:41:32
Comments		
Input	Data	D:\D\project\IL6 and BTC\manuscript\frontiers in immunology\Expression and prognosis.sav
	Active Dataset	1
	Filter	<none>
	Weight	<none>
	Split File	<none>
	N of Rows in Working Data File	91
Missing Value Handling	Definition of Missing	User-defined missing values are treated as missing.
	Cases Used	Statistics for each table are based on all the cases with valid data in the specified range(s) for all variables in each table.
Syntax		CROSSTABS<br>/TABLES=sex BY IL6R CRP STAT3 CK19 GP130 JAK2 IL6<br>/FORMAT=AVALUE TABLES<br>/STATISTICS=CHISQ<br>/CELLS=COUNT COLUMN<br>/COUNT ROUND CELL.
Resources	Processor Time	00:00:00.02
	Elapsed Time	00:00:00.02
	Dimensions Requested	2
	Cells Available	524245

sex * IL6R

Crosstab

			IL6R		Total
			0	1	
sex	0	Count	23	25	48
		% within IL6R	51.1%	54.3%	52.7%
	1	Count	22	21	43
		% within IL6R	48.9%	45.7%	47.3%
Total		Count	45	46	91
		% within IL6R	100.0%	100.0%	100.0%

Chi-Square Tests

	Value	df	Asymptotic Significance (2-sided)	Exact Sig. (2-sided)	Exact Sig. (1-sided)
Pearson Chi-Square	.096[a]	1	.757		
Continuity Correction[b]	.010	1	.921		
Likelihood Ratio	.096	1	.757		
Fisher's Exact Test				.835	.461
Linear-by-Linear Association	.095	1	.758		
N of Valid Cases	91				

a. 0 cells (0.0%) have expected count less than 5. The minimum expected count is 21.26.

b. Computed only for a 2x2 table

sex * CRP

Crosstab

			CRP		Total
			0	1	
sex	0	Count	26	22	48
		% within CRP	46.4%	62.9%	52.7%
	1	Count	30	13	43
		% within CRP	53.6%	37.1%	47.3%
Total		Count	56	35	91
		% within CRP	100.0%	100.0%	100.0%

Chi-Square Tests

	Value	df	Asymptotic Significance (2-sided)	Exact Sig. (2-sided)	Exact Sig. (1-sided)
Pearson Chi-Square	2.332[a]	1	.127		
Continuity Correction[b]	1.720	1	.190		
Likelihood Ratio	2.352	1	.125		
Fisher's Exact Test				.138	.095
Linear-by-Linear Association	2.307	1	.129		
N of Valid Cases	91				

a. 0 cells (0.0%) have expected count less than 5. The minimum expected count is 16.54.

b. Computed only for a 2x2 table

sex * STAT3

Crosstab

			STAT3		Total
			0	1	
sex	0	Count	30	18	48
		% within STAT3	53.6%	51.4%	52.7%
	1	Count	26	17	43
		% within STAT3	46.4%	48.6%	47.3%
Total		Count	56	35	91
		% within STAT3	100.0%	100.0%	100.0%

Chi-Square Tests

	Value	df	Asymptotic Significance (2-sided)	Exact Sig. (2-sided)	Exact Sig. (1-sided)
Pearson Chi-Square	.040[a]	1	.842		
Continuity Correction[b]	.000	1	1.000		
Likelihood Ratio	.040	1	.842		
Fisher's Exact Test				1.000	.506
Linear-by-Linear Association	.039	1	.843		
N of Valid Cases	91				

a. 0 cells (0.0%) have expected count less than 5. The minimum expected count is 16.54.

b. Computed only for a 2x2 table

sex * CK19

Crosstab

			CK19		Total
			0	1	
sex	0	Count	24	24	48
		% within CK19	49.0%	57.1%	52.7%
	1	Count	25	18	43
		% within CK19	51.0%	42.9%	47.3%
Total		Count	49	42	91
		% within CK19	100.0%	100.0%	100.0%

Chi-Square Tests

	Value	df	Asymptotic Significance (2-sided)	Exact Sig. (2-sided)	Exact Sig. (1-sided)
Pearson Chi-Square	.605[a]	1	.437		
Continuity Correction[b]	.321	1	.571		
Likelihood Ratio	.606	1	.436		
Fisher's Exact Test				.529	.286
Linear-by-Linear Association	.598	1	.439		
N of Valid Cases	91				

a. 0 cells (0.0%) have expected count less than 5. The minimum expected count is 19.85.

b. Computed only for a 2x2 table

sex * GP130

Crosstab

			GP130		Total
			0	1	
sex	0	Count	24	24	48
		% within GP130	43.6%	66.7%	52.7%
	1	Count	31	12	43
		% within GP130	56.4%	33.3%	47.3%
Total		Count	55	36	91
		% within GP130	100.0%	100.0%	100.0%

Chi-Square Tests

	Value	df	Asymptotic Significance (2-sided)	Exact Sig. (2-sided)	Exact Sig. (1-sided)
Pearson Chi-Square	4.630[a]	1	.031		
Continuity Correction[b]	3.752	1	.053		
Likelihood Ratio	4.696	1	.030		
Fisher's Exact Test				.035	.026
Linear-by-Linear Association	4.579	1	.032		
N of Valid Cases	91				

a. 0 cells (0.0%) have expected count less than 5. The minimum expected count is 17.01.

b. Computed only for a 2x2 table

sex * JAK2

Crosstab

			JAK2		Total
			0	1	
sex	0	Count	30	18	48
		% within JAK2	51.7%	54.5%	52.7%
	1	Count	28	15	43
		% within JAK2	48.3%	45.5%	47.3%
Total		Count	58	33	91
		% within JAK2	100.0%	100.0%	100.0%

Chi-Square Tests

	Value	df	Asymptotic Significance (2-sided)	Exact Sig. (2-sided)	Exact Sig. (1-sided)
Pearson Chi-Square	.067[a]	1	.796		
Continuity Correction[b]	.002	1	.967		
Likelihood Ratio	.067	1	.795		
Fisher's Exact Test				.830	.484
Linear-by-Linear Association	.066	1	.797		
N of Valid Cases	91				

a. 0 cells (0.0%) have expected count less than 5. The minimum expected count is 15.59.

b. Computed only for a 2x2 table

sex * IL6

Crosstab

			IL6		Total
			0	1	
sex	0	Count	30	18	48
		% within IL6	63.8%	40.9%	52.7%
	1	Count	17	26	43
		% within IL6	36.2%	59.1%	47.3%
Total		Count	47	44	91
		% within IL6	100.0%	100.0%	100.0%

Chi-Square Tests

	Value	df	Asymptotic Significance (2-sided)	Exact Sig. (2-sided)	Exact Sig. (1-sided)
Pearson Chi-Square	4.790[a]	1	.029		
Continuity Correction[b]	3.915	1	.048		
Likelihood Ratio	4.831	1	.028		
Fisher's Exact Test				.036	.024
Linear-by-Linear Association	4.737	1	.030		
N of Valid Cases	91				

a. 0 cells (0.0%) have expected count less than 5. The minimum expected count is 20.79.

b. Computed only for a 2x2 table

```
NPAR TESTS
 /M-W= IL6R_expression CRP_expression STAT3_expression CK19_expression GP130_
expression
  JAK2_expression IL6_expression IL6R_CRP_expression IL6R_STAT3_expression IL6R_
CRP_STAT3_expression
  GP130_IL6_expression JAK2_IL6_expression GP130_JAK2_IL6_expression BY Vascular
_invasion(0 1)
 /MISSING ANALYSIS.
```

NPar Tests

Notes

Output Created		25-JAN-2024 08:45:56
Comments		
Input	Data	D:\D\project\IL6 and BTC\manuscript\frontiers in immunology\Expression and prognosis.sav
	Active Dataset	1
	Filter	<none>
	Weight	<none>
	Split File	<none>
	N of Rows in Working Data File	91
Missing Value Handling	Definition of Missing	User-defined missing values are treated as missing.
	Cases Used	Statistics for each test are based on all cases with valid data for the variable(s) used in that test.
Syntax		NPAR TESTS<br>/M-W= IL6R_expression CRP_expression STAT3_expression CK19_expression GP130_expression<br>JAK2_expression IL6_expression IL6R_CRP_expression IL6R_STAT3_expression IL6R_CRP_STAT3_expression<br>GP130_IL6_expression JAK2_IL6_expression GP130_JAK2_IL6_expression BY Vascular_invasion(0 1)<br>/MISSING ANALYSIS.
Resources	Processor Time	00:00:00.02
	Elapsed Time	00:00:00.00
	Number of Cases Allowed[a]	165564

a. Based on availability of workspace memory.

Mann-Whitney Test

Ranks

	Vascular_invasion	N	Mean Rank	Sum of Ranks
IL6R_expression	0	62	44.73	2773.00
	1	29	48.72	1413.00
	Total	91		
CRP_expression	0	62	51.53	3195.00
	1	29	34.17	991.00
	Total	91		
STAT3_expression	0	62	49.73	3083.00
	1	29	38.03	1103.00
	Total	91		
CK19_expression	0	62	46.24	2867.00
	1	29	45.48	1319.00
	Total	91		
GP130_expression	0	62	48.97	3036.00
	1	29	39.66	1150.00
	Total	91		
JAK2_expression	0	62	48.94	3034.00
	1	29	39.72	1152.00
	Total	91		
IL6_expression	0	62	49.08	3043.00
	1	29	39.41	1143.00
	Total	91		
IL6R_CRP_expression	0	62	51.94	3220.50
	1	29	33.29	965.50
	Total	91		
IL6R_STAT3_expression	0	62	47.45	2942.00
	1	29	42.90	1244.00
	Total	91		
IL6R_CRP_STAT3_expression	0	62	52.32	3244.00
	1	29	32.48	942.00
	Total	91		
GP130_IL6_expression	0	62	49.90	3094.00
	1	29	37.66	1092.00
	Total	91		
JAK2_IL6_expression	0	62	51.34	3183.00
	1	29	34.59	1003.00
	Total	91		
GP130_JAK2_IL6_expression	0	62	51.15	3171.00
	1	29	35.00	1015.00
	Total	91		

Test Statistics[a]

	IL6R_expression	CRP_expression	STAT3_expression	CK19_expression	GP130_expression	JAK2_expression	IL6_expression	IL6R_CRP_expression	IL6R_STAT3_expression	IL6R_CRP_STAT3_expression	GP130_IL6_expression	JAK2_IL6_expression	GP130_JAK2_IL6_expression
Mann-Whitney U	820.000	556.000	668.000	884.000	715.000	717.000	708.000	530.500	809.000	507.000	657.000	568.000	580.000
Wilcoxon W	2773.000	991.000	1103.000	1319.000	1150.000	1152.000	1143.000	965.500	1244.000	942.000	1092.000	1003.000	1015.000
Z	-.673	-2.921	-1.967	-.128	-1.567	-1.550	-1.627	-3.139	-.767	-3.339	-2.061	-2.819	-2.717
Asymp. Sig. (2-tailed)	.501	.003	.049	.898	.117	.121	.104	.002	.443	.001	.039	.005	.007

a. Grouping Variable: Vascular_invasion

```
COXREG Survival_month
/STATUS=Death(1)
/METHOD=ENTER IL6R_expression
/PRINT=CI(95)SUMMARY
/CRITERIA=PIN(.05)POUT(.10)ITERATE(20).
```

Cox Regression

Notes

Output Created		25-JAN-2024 08:48:01
Comments		
Input	Data	D:\D\project\IL6 and BTC\manuscript\frontiers in immunology\Expression and prognosis.sav
	Active Dataset	1
	Filter	<none>
	Weight	<none>
	Split File	<none>
	N of Rows in Working Data File	91
Missing Value Handling	Definition of Missing	User-defined missing values are treated as missing.
Syntax		COXREG Survival_month /STATUS=Death(1) /METHOD=ENTER IL6R_expression /PRINT=CI(95) SUMMARY /CRITERIA=PIN(.05) POUT(.10) ITERATE(20).
Resources	Processor Time	00:00:00.00
	Elapsed Time	00:00:00.02

Case Processing Summary

		N	Percent
Cases available in analysis	Event[a]	69	75.8%
	Censored	12	13.2%
	Total	81	89.0%
Cases dropped	Cases with missing values	10	11.0%
	Cases with negative time	0	0.0%
	Censored cases before the earliest event in a stratum	0	0.0%
	Total	10	11.0%
Total		91	100.0%

a. Dependent Variable: Survival_month

Block 0: Beginning Block

Omnibus Tests of Model Coefficients

-2 Log Likelihood
515.861

Block 1: Method = Enter

Omnibus Tests of Model Coefficients[a]

-2 Log Likelihood	Overall (score)			Change From Previous Step			Change From Previous Block		
	Chi-square	df	Sig.	Chi-square	df	Sig.	Chi-square	df	Sig.
514.194	1.566	1	.211	1.667	1	.197	1.667	1	.197

a. Beginning Block Number 1. Method = Enter

Variables in the Equation

	B	SE	Wald	df	Sig.	Exp(B)	95.0% CI for Exp(B)	
							Lower	Upper
IL6R_expression	-.006	.005	1.567	1	.211	.994	.984	1.004

Covariate Means

	Mean
IL6R_expression	78.765

```
COXREG Survival_month
/STATUS=Death(1)
```

```
/METHOD=ENTER IL6R
/PRINT=CI(95)SUMMARY
/CRITERIA=PIN(.05)POUT(.10)ITERATE(20).
```

Cox Regression

Notes

Output Created		25-JAN-2024 08:48:37
Comments		
Input	Data	D:\D\project\IL6 and BTC\manuscript\frontiers in immunology\Expression and prognosis.sav
	Active Dataset	1
	Filter	<none>
	Weight	<none>
	Split File	<none>
	N of Rows in Working Data File	91
Missing Value Handling	Definition of Missing	User-defined missing values are treated as missing.
Syntax		COXREG Survival_month /STATUS=Death(1) /METHOD=ENTER IL6R /PRINT=CI(95) SUMMARY /CRITERIA=PIN(.05) POUT(.10) ITERATE(20).
Resources	Processor Time	00:00:00.00
	Elapsed Time	00:00:00.01

Case Processing Summary

		N	Percent
Cases available in analysis	Event[a]	69	75.8%
	Censored	12	13.2%
	Total	81	89.0%
Cases dropped	Cases with missing values	10	11.0%
	Cases with negative time	0	0.0%
	Censored cases before the earliest event in a stratum	0	0.0%
	Total	10	11.0%
Total		91	100.0%

a. Dependent Variable: Survival_month

Block 0: Beginning Block

Omnibus Tests of Model Coefficients

-2 Log Likelihood
515.861

Block 1: Method = Enter

Omnibus Tests of Model Coefficients[a]

-2 Log Likelihood	Overall (score)			Change From Previous Step			Change From Previous Block		
	Chi-square	df	Sig.	Chi-square	df	Sig.	Chi-square	df	Sig.
515.309	.552	1	.457	.552	1	.457	.552	1	.457

a. Beginning Block Number 1. Method = Enter

Variables in the Equation

	B	SE	Wald	df	Sig.	Exp(B)	95.0% CI for Exp(B)	
							Lower	Upper
IL6R	-.180	.243	.551	1	.458	.835	.519	1.344

Covariate Means

	Mean
IL6R	.494

```
COXREG Survival_month
/STATUS=Death(1)
/CONTRAST (CRP)=Indicator(1)
/METHOD=ENTER CRP
/PRINT=CI(95)SUMMARY
/CRITERIA=PIN(.05)POUT(.10)ITERATE(20).
```

Cox Regression

Notes

Output Created		25-JAN-2024 08:49:31
Comments		
Input	Data	D:\D\project\IL6 and BTC\manuscript\frontiers in immunology\Expression and prognosis.sav
	Active Dataset	1
	Filter	<none>
	Weight	<none>
	Split File	<none>
	N of Rows in Working Data File	91
Missing Value Handling	Definition of Missing	User-defined missing values are treated as missing.
Syntax		COXREG Survival_month /STATUS=Death(1) /CONTRAST (CRP)=Indicator(1) /METHOD=ENTER CRP /PRINT=CI(95) SUMMARY /CRITERIA=PIN(.05) POUT(.10) ITERATE(20).
Resources	Processor Time	00:00:00.02
	Elapsed Time	00:00:00.01

Case Processing Summary

		N	Percent
Cases available in analysis	Event[a]	69	75.8%
	Censored	12	13.2%
	Total	81	89.0%
Cases dropped	Cases with missing values	10	11.0%
	Cases with negative time	0	0.0%
	Censored cases before the earliest event in a stratum	0	0.0%
	Total	10	11.0%
Total		91	100.0%

a. Dependent Variable: Survival_month

Categorical Variable Codings[a]

		Frequency	(1)
CRP[b]	0	50	0
	1	31	1

a. Category variable: CRP

b. Indicator Parameter Coding

Block 0: Beginning Block

Omnibus Tests of Model Coefficients

-2 Log Likelihood
515.861

Block 1: Method = Enter

Omnibus Tests of Model Coefficients[a]

-2 Log Likelihood	Overall (score)			Change From Previous Step			Change From Previous Block		
	Chi-square	df	Sig.	Chi-square	df	Sig.	Chi-square	df	Sig.
515.621	.243	1	.622	.240	1	.624	.240	1	.624

a. Beginning Block Number 1. Method = Enter

Variables in the Equation

	B	SE	Wald	df	Sig.	Exp(B)	95.0% CI for Exp(B)	
							Lower	Upper
CRP	.123	.250	.243	1	.622	1.131	.693	1.845

Covariate Means

	Mean
CRP	.383

```
COXREG Survival_month
/STATUS=Death(1)
/METHOD=ENTER STAT3
/PRINT=CI(95)SUMMARY
/CRITERIA=PIN(.05)POUT(.10)ITERATE(20).
```

Cox Regression

Notes

Output Created		25-JAN-2024 08:49:52
Comments		
Input	Data	D:\D\project\IL6 and BTC\manuscript\frontiers in immunology\Expression and prognosis.sav
	Active Dataset	1
	Filter	<none>
	Weight	<none>
	Split File	<none>
	N of Rows in Working Data File	91
Missing Value Handling	Definition of Missing	User-defined missing values are treated as missing.
Syntax		COXREG Survival_month<br>/STATUS=Death(1)<br>/METHOD=ENTER STAT3<br>/PRINT=CI(95) SUMMARY<br>/CRITERIA=PIN(.05) POUT(.10) ITERATE(20).
Resources	Processor Time	00:00:00.00
	Elapsed Time	00:00:00.01

Case Processing Summary

		N	Percent
Cases available in analysis	Event[a]	69	75.8%
	Censored	12	13.2%
	Total	81	89.0%
Cases dropped	Cases with missing values	10	11.0%
	Cases with negative time	0	0.0%
	Censored cases before the earliest event in a stratum	0	0.0%
	Total	10	11.0%
Total		91	100.0%

a. Dependent Variable: Survival_month

Block 0: Beginning Block

Omnibus Tests of Model Coefficients

-2 Log Likelihood
515.861

Block 1: Method = Enter

Omnibus Tests of Model Coefficients[a]

-2 Log Likelihood	Overall (score)			Change From Previous Step			Change From Previous Block		
	Chi-square	df	Sig.	Chi-square	df	Sig.	Chi-square	df	Sig.
514.242	1.586	1	.208	1.619	1	.203	1.619	1	.203

a. Beginning Block Number 1. Method = Enter

Variables in the Equation

	B	SE	Wald	df	Sig.	Exp(B)	95.0% CI for Exp(B)	
							Lower	Upper
STAT3	-.324	.258	1.574	1	.210	.723	.436	1.200

Covariate Means

	Mean
STAT3	.383

```
COXREG Survival_month
/STATUS=Death(1)
/CONTRAST (GP130)=Indicator(1)
/METHOD=ENTER GP130
/PRINT=CI(95)SUMMARY
/CRITERIA=PIN(.05)POUT(.10)ITERATE(20).
```

Cox Regression

Notes

Output Created		25-JAN-2024 08:50:21
Comments		
Input	Data	D:\D\project\IL6 and BTC\manuscript\frontiers in immunology\Expression and prognosis.sav
	Active Dataset	1
	Filter	<none>
	Weight	<none>
	Split File	<none>
	N of Rows in Working Data File	91
Missing Value Handling	Definition of Missing	User-defined missing values are treated as missing.
Syntax		COXREG Survival_month /STATUS=Death(1) /CONTRAST (GP130)=Indicator(1) /METHOD=ENTER GP130 /PRINT=CI(95) SUMMARY /CRITERIA=PIN(.05) POUT(.10) ITERATE(20).
Resources	Processor Time	00:00:00.00
	Elapsed Time	00:00:00.01

Case Processing Summary

		N	Percent
Cases available in analysis	Event[a]	69	75.8%
	Censored	12	13.2%
	Total	81	89.0%
Cases dropped	Cases with missing values	10	11.0%
	Cases with negative time	0	0.0%
	Censored cases before the earliest event in a stratum	0	0.0%
	Total	10	11.0%
Total		91	100.0%

a. Dependent Variable: Survival_month

Categorical Variable Codings[a]

		Frequency	(1)
GP130[b]	0	49	0
	1	32	1

a. Category variable: GP130

b. Indicator Parameter Coding

Block 0: Beginning Block

Omnibus Tests of Model Coefficients

-2 Log Likelihood
515.861

Block 1: Method = Enter

Omnibus Tests of Model Coefficients[a]

-2 Log Likelihood	Overall (score)			Change From Previous Step			Change From Previous Block		
	Chi-square	df	Sig.	Chi-square	df	Sig.	Chi-square	df	Sig.
514.766	1.081	1	.298	1.095	1	.295	1.095	1	.295

a. Beginning Block Number 1. Method = Enter

Variables in the Equation

	B	SE	Wald	df	Sig.	Exp(B)	95.0% CI for Exp(B)	
							Lower	Upper
GP130	-.259	.250	1.075	1	.300	.772	.473	1.259

Covariate Means

	Mean
GP130	.395

```
COXREG Survival_month
/STATUS=Death(1)
/METHOD=ENTER JAK2
/PRINT=CI(95)SUMMARY
/CRITERIA=PIN(.05)POUT(.10)ITERATE(20).
```

Cox Regression

Notes

Output Created		25-JAN-2024 08:50:39
Comments		
Input	Data	D:\D\project\IL6 and BTC\manuscript\frontiers in immunology\Expression and prognosis.sav
	Active Dataset	1
	Filter	<none>
	Weight	<none>
	Split File	<none>
	N of Rows in Working Data File	91
Missing Value Handling	Definition of Missing	User-defined missing values are treated as missing.
Syntax		COXREG Survival_month /STATUS=Death(1) /METHOD=ENTER JAK2 /PRINT=CI(95) SUMMARY /CRITERIA=PIN(.05) POUT(.10) ITERATE(20).
Resources	Processor Time	00:00:00.00
	Elapsed Time	00:00:00.00

Case Processing Summary

		N	Percent
Cases available in analysis	Event[a]	69	75.8%
	Censored	12	13.2%
	Total	81	89.0%
Cases dropped	Cases with missing values	10	11.0%
	Cases with negative time	0	0.0%
	Censored cases before the earliest event in a stratum	0	0.0%
	Total	10	11.0%
Total		91	100.0%

a. Dependent Variable: Survival_month

Block 0: Beginning Block

Omnibus Tests of Model Coefficients

-2 Log Likelihood
515.861

Block 1: Method = Enter

Omnibus Tests of Model Coefficients[a]

-2 Log Likelihood	Overall (score)			Change From Previous Step			Change From Previous Block		
	Chi-square	df	Sig.	Chi-square	df	Sig.	Chi-square	df	Sig.
515.719	.141	1	.707	.142	1	.706	.142	1	.706

a. Beginning Block Number 1. Method = Enter

Variables in the Equation

	B	SE	Wald	df	Sig.	Exp(B)	95.0% CI for Exp(B)	
							Lower	Upper
JAK2	-.094	.251	.141	1	.707	.910	.557	1.488

Covariate Means

	Mean
JAK2	.383

```
COXREG Survival_month
/STATUS=Death(1)
/METHOD=ENTER IL6
/PRINT=CI(95)SUMMARY
/CRITERIA=PIN(.05)POUT(.10)ITERATE(20).
```

Cox Regression

Notes

Output Created		25-JAN-2024 08:50:50
Comments		
Input	Data	D:\D\project\IL6 and BTC\manuscript\frontiers in immunology\Expression and prognosis.sav
	Active Dataset	1
	Filter	<none>
	Weight	<none>
	Split File	<none>
	N of Rows in Working Data File	91
Missing Value Handling	Definition of Missing	User-defined missing values are treated as missing.
Syntax		COXREG Survival_month<br>/STATUS=Death(1)<br>/METHOD=ENTER IL6<br>/PRINT=CI(95) SUMMARY<br>/CRITERIA=PIN(.05) POUT(.10) ITERATE(20).
Resources	Processor Time	00:00:00.00
	Elapsed Time	00:00:00.01

Case Processing Summary

		N	Percent
Cases available in analysis	Event[a]	69	75.8%
	Censored	12	13.2%
	Total	81	89.0%
Cases dropped	Cases with missing values	10	11.0%
	Cases with negative time	0	0.0%
	Censored cases before the earliest event in a stratum	0	0.0%
	Total	10	11.0%
Total		91	100.0%

a. Dependent Variable: Survival_month

Block 0: Beginning Block

Omnibus Tests of Model Coefficients

-2 Log Likelihood
515.861

Block 1: Method = Enter

Omnibus Tests of Model Coefficients[a]

-2 Log Likelihood	Overall (score)			Change From Previous Step			Change From Previous Block		
	Chi-square	df	Sig.	Chi-square	df	Sig.	Chi-square	df	Sig.
515.403	.457	1	.499	.458	1	.499	.458	1	.499

a. Beginning Block Number 1. Method = Enter

Variables in the Equation

	B	SE	Wald	df	Sig.	Exp(B)	95.0% CI for Exp(B)	
							Lower	Upper
IL6	-.164	.243	.456	1	.500	.849	.527	1.367

Covariate Means

	Mean
IL6	.469

```
COXREG DFS_month
/STATUS=Recurrence(1)
/METHOD=ENTER IL6
/PRINT=CI(95)SUMMARY
/CRITERIA=PIN(.05)POUT(.10)ITERATE(20).
```

Cox Regression

Notes

Output Created		25-JAN-2024 08:51:18
Comments		
Input	Data	D:\D\project\IL6 and BTC\manuscript\frontiers in immunology\Expression and prognosis.sav
	Active Dataset	1
	Filter	<none>
	Weight	<none>
	Split File	<none>
	N of Rows in Working Data File	91
Missing Value Handling	Definition of Missing	User-defined missing values are treated as missing.
Syntax		COXREG DFS_month<br>/STATUS=Recurrence(1)<br>/METHOD=ENTER IL6<br>/PRINT=CI(95) SUMMARY<br>/CRITERIA=PIN(.05) POUT(.10) ITERATE(20).
Resources	Processor Time	00:00:00.00
	Elapsed Time	00:00:00.01

Case Processing Summary

		N	Percent
Cases available in analysis	Event[a]	65	71.4%
	Censored	15	16.5%
	Total	80	87.9%
Cases dropped	Cases with missing values	11	12.1%
	Cases with negative time	0	0.0%
	Censored cases before the earliest event in a stratum	0	0.0%
	Total	11	12.1%
Total		91	100.0%

a. Dependent Variable: DFS_month

Block 0: Beginning Block

Omnibus Tests of Model Coefficients

-2 Log Likelihood
487.446

Block 1: Method = Enter

Omnibus Tests of Model Coefficients[a]

-2 Log Likelihood	Overall (score)			Change From Previous Step			Change From Previous Block		
	Chi-square	df	Sig.	Chi-square	df	Sig.	Chi-square	df	Sig.
486.258	1.184	1	.277	1.188	1	.276	1.188	1	.276

a. Beginning Block Number 1. Method = Enter

Variables in the Equation

	B	SE	Wald	df	Sig.	Exp(B)	95.0% CI for Exp(B)	
							Lower	Upper
IL6	-.273	.252	1.177	1	.278	.761	.465	1.246

Covariate Means

	Mean
IL6	.463

```
COXREG DFS_month
/STATUS=Recurrence(1)
/METHOD=ENTER JAK2
/PRINT=CI(95)SUMMARY
/CRITERIA=PIN(.05)POUT(.10)ITERATE(20).
```

Cox Regression

Notes

Output Created		25-JAN-2024 08:51:25
Comments		
Input	Data	D:\D\project\IL6 and BTC\manuscript\frontiers in immunology\Expression and prognosis.sav
	Active Dataset	1
	Filter	<none>
	Weight	<none>
	Split File	<none>
	N of Rows in Working Data File	91
Missing Value Handling	Definition of Missing	User-defined missing values are treated as missing.
Syntax		COXREG DFS_month /STATUS=Recurrence(1) /METHOD=ENTER JAK2 /PRINT=CI(95) SUMMARY /CRITERIA=PIN(.05) POUT(.10) ITERATE(20).
Resources	Processor Time	00:00:00.00
	Elapsed Time	00:00:00.01

Case Processing Summary

		N	Percent
Cases available in analysis	Event[a]	65	71.4%
	Censored	15	16.5%
	Total	80	87.9%
Cases dropped	Cases with missing values	11	12.1%
	Cases with negative time	0	0.0%
	Censored cases before the earliest event in a stratum	0	0.0%
	Total	11	12.1%
Total		91	100.0%

a. Dependent Variable: DFS_month

Block 0: Beginning Block

Omnibus Tests of Model Coefficients

-2 Log Likelihood
487.446

Block 1: Method = Enter

Omnibus Tests of Model Coefficients[a]

-2 Log Likelihood	Overall (score)			Change From Previous Step			Change From Previous Block		
	Chi-square	df	Sig.	Chi-square	df	Sig.	Chi-square	df	Sig.
486.812	.624	1	.430	.634	1	.426	.634	1	.426

a. Beginning Block Number 1. Method = Enter

Variables in the Equation

	B	SE	Wald	df	Sig.	Exp(B)	95.0% CI for Exp(B)	
							Lower	Upper
JAK2	-.207	.263	.621	1	.431	.813	.486	1.360

Covariate Means

	Mean
JAK2	.375

```
COXREG DFS_month
/STATUS=Recurrence(1)
/CONTRAST (GP130)=Indicator(1)
/METHOD=ENTER GP130
/PRINT=CI(95)SUMMARY
/CRITERIA=PIN(.05)POUT(.10)ITERATE(20).
```

Cox Regression

Notes

Output Created		25-JAN-2024 08:51:32
Comments		
Input	Data	D:\D\project\IL6 and BTC\manuscript\frontiers in immunology\Expression and prognosis.sav
	Active Dataset	1
	Filter	<none>
	Weight	<none>
	Split File	<none>
	N of Rows in Working Data File	91
Missing Value Handling	Definition of Missing	User-defined missing values are treated as missing.
Syntax		COXREG DFS_month /STATUS=Recurrence(1) /CONTRAST (GP130)=Indicator(1) /METHOD=ENTER GP130 /PRINT=CI(95) SUMMARY /CRITERIA=PIN(.05) POUT(.10) ITERATE(20).
Resources	Processor Time	00:00:00.00
	Elapsed Time	00:00:00.00

Case Processing Summary

		N	Percent
Cases available in analysis	Event[a]	65	71.4%
	Censored	15	16.5%
	Total	80	87.9%
Cases dropped	Cases with missing values	11	12.1%
	Cases with negative time	0	0.0%
	Censored cases before the earliest event in a stratum	0	0.0%
	Total	11	12.1%
Total		91	100.0%

a. Dependent Variable: DFS_month

Categorical Variable Codings[a]

		Frequency	(1)
GP130[b]	0	48	0
	1	32	1

a. Category variable: GP130

b. Indicator Parameter Coding

Block 0: Beginning Block

Omnibus Tests of Model Coefficients

-2 Log Likelihood
487.446

Block 1: Method = Enter

Omnibus Tests of Model Coefficients[a]

-2 Log Likelihood	Overall (score)			Change From Previous Step			Change From Previous Block		
	Chi-square	df	Sig.	Chi-square	df	Sig.	Chi-square	df	Sig.
486.823	.617	1	.432	.623	1	.430	.623	1	.430

a. Beginning Block Number 1. Method = Enter

Variables in the Equation

	B	SE	Wald	df	Sig.	Exp(B)	95.0% CI for Exp(B)	
							Lower	Upper
GP130	-.201	.256	.615	1	.433	.818	.495	1.352

Covariate Means

	Mean
GP130	.400

```
COXREG DFS_month
/STATUS=Recurrence(1)
/METHOD=ENTER STAT3
/PRINT=CI(95)SUMMARY
/CRITERIA=PIN(.05)POUT(.10)ITERATE(20).
```

Cox Regression

Notes

Output Created		25-JAN-2024 08:51:39
Comments		
Input	Data	D:\D\project\IL6 and BTC\manuscript\frontiers in immunology\Expression and prognosis.sav
	Active Dataset	1
	Filter	<none>
	Weight	<none>
	Split File	<none>
	N of Rows in Working Data File	91
Missing Value Handling	Definition of Missing	User-defined missing values are treated as missing.
Syntax		COXREG DFS_month /STATUS=Recurrence(1) /METHOD=ENTER STAT3 /PRINT=CI(95) SUMMARY /CRITERIA=PIN(.05) POUT(.10) ITERATE(20).
Resources	Processor Time	00:00:00.00
	Elapsed Time	00:00:00.01

Case Processing Summary

		N	Percent
Cases available in analysis	Event[a]	65	71.4%
	Censored	15	16.5%
	Total	80	87.9%
Cases dropped	Cases with missing values	11	12.1%
	Cases with negative time	0	0.0%
	Censored cases before the earliest event in a stratum	0	0.0%
	Total	11	12.1%
Total		91	100.0%

a. Dependent Variable: DFS_month

Block 0: Beginning Block

Omnibus Tests of Model Coefficients

-2 Log Likelihood
487.446

Block 1: Method = Enter

Omnibus Tests of Model Coefficients[a]

-2 Log Likelihood	Overall (score)			Change From Previous Step			Change From Previous Block		
	Chi-square	df	Sig.	Chi-square	df	Sig.	Chi-square	df	Sig.
485.560	1.843	1	.175	1.886	1	.170	1.886	1	.170

a. Beginning Block Number 1. Method = Enter

Variables in the Equation

	B	SE	Wald	df	Sig.	Exp(B)	95.0% CI for Exp(B)	
							Lower	Upper
STAT3	-.358	.265	1.824	1	.177	.699	.415	1.175

Covariate Means

	Mean
STAT3	.388

```
COXREG DFS_month
/STATUS=Recurrence(1)
/METHOD=ENTER CRP
/PRINT=CI(95)SUMMARY
/CRITERIA=PIN(.05)POUT(.10)ITERATE(20).
```

Cox Regression

Notes

Output Created		25-JAN-2024 08:51:45
Comments		
Input	Data	D:\D\project\IL6 and BTC\manuscript\frontiers in immunology\Expression and prognosis.sav
	Active Dataset	1
	Filter	<none>
	Weight	<none>
	Split File	<none>
	N of Rows in Working Data File	91
Missing Value Handling	Definition of Missing	User-defined missing values are treated as missing.
Syntax		COXREG DFS_month /STATUS=Recurrence(1) /METHOD=ENTER CRP /PRINT=CI(95) SUMMARY /CRITERIA=PIN(.05) POUT(.10) ITERATE(20).
Resources	Processor Time	00:00:00.02
	Elapsed Time	00:00:00.01

Case Processing Summary

		N	Percent
Cases available in analysis	Event[a]	65	71.4%
	Censored	15	16.5%
	Total	80	87.9%
Cases dropped	Cases with missing values	11	12.1%
	Cases with negative time	0	0.0%
	Censored cases before the earliest event in a stratum	0	0.0%
	Total	11	12.1%
Total		91	100.0%

a. Dependent Variable: DFS_month

Block 0: Beginning Block

Omnibus Tests of Model Coefficients

-2 Log Likelihood
487.446

Block 1: Method = Enter

Omnibus Tests of Model Coefficients[a]

-2 Log Likelihood	Overall (score)			Change From Previous Step			Change From Previous Block		
	Chi-square	df	Sig.	Chi-square	df	Sig.	Chi-square	df	Sig.
487.424	.022	1	.881	.022	1	.881	.022	1	.881

a. Beginning Block Number 1. Method = Enter

Variables in the Equation

	B	SE	Wald	df	Sig.	Exp(B)	95.0% CI for Exp(B)	
							Lower	Upper
CRP	.039	.261	.022	1	.881	1.040	.623	1.734

Covariate Means

	Mean
CRP	.375

```
COXREG DFS_month
/STATUS=Recurrence(1)
/METHOD=ENTER IL6R
/PRINT=CI(95)SUMMARY
/CRITERIA=PIN(.05)POUT(.10)ITERATE(20).
```

Cox Regression

Notes

Output Created		25-JAN-2024 08:51:52
Comments		
Input	Data	D:\D\project\IL6 and BTC\manuscript\frontiers in immunology\Expression and prognosis.sav
	Active Dataset	1
	Filter	<none>
	Weight	<none>
	Split File	<none>
	N of Rows in Working Data File	91
Missing Value Handling	Definition of Missing	User-defined missing values are treated as missing.
Syntax		COXREG DFS_month /STATUS=Recurrence(1) /METHOD=ENTER IL6R /PRINT=CI(95) SUMMARY /CRITERIA=PIN(.05) POUT(.10) ITERATE(20).
Resources	Processor Time	00:00:00.00
	Elapsed Time	00:00:00.01

Case Processing Summary

		N	Percent
Cases available in analysis	Event[a]	65	71.4%
	Censored	15	16.5%
	Total	80	87.9%
Cases dropped	Cases with missing values	11	12.1%
	Cases with negative time	0	0.0%
	Censored cases before the earliest event in a stratum	0	0.0%
	Total	11	12.1%
Total		91	100.0%

a. Dependent Variable: DFS_month

Block 0: Beginning Block

Omnibus Tests of Model Coefficients

-2 Log Likelihood
487.446

Block 1: Method = Enter

Omnibus Tests of Model Coefficients[a]

-2 Log Likelihood	Overall (score)			Change From Previous Step			Change From Previous Block		
	Chi-square	df	Sig.	Chi-square	df	Sig.	Chi-square	df	Sig.
487.125	.320	1	.571	.321	1	.571	.321	1	.571

a. Beginning Block Number 1. Method = Enter

Variables in the Equation

	B	SE	Wald	df	Sig.	Exp(B)	95.0% CI for Exp(B)	
							Lower	Upper
IL6R	-.141	.250	.320	1	.572	.868	.532	1.416

Covariate Means

	Mean
IL6R	.500

```
COXREG Survival_month
/STATUS=Death(1)
/METHOD=ENTER sex
/PRINT=CI(95)SUMMARY
/CRITERIA=PIN(.05)POUT(.10)ITERATE(20).
```

Cox Regression

Notes

Output Created		25-JAN-2024 08:54:39
Comments		
Input	Data	D:\D\project\IL6 and BTC\manuscript\frontiers in immunology\Expression and prognosis.sav
	Active Dataset	1
	Filter	<none>
	Weight	<none>
	Split File	<none>
	N of Rows in Working Data File	91
Missing Value Handling	Definition of Missing	User-defined missing values are treated as missing.
Syntax		COXREG Survival_month<br>/STATUS=Death(1)<br>/METHOD=ENTER sex<br>/PRINT=CI(95) SUMMARY<br>/CRITERIA=PIN(.05) POUT(.10)<br>ITERATE(20).
Resources	Processor Time	00:00:00.02
	Elapsed Time	00:00:00.01

Case Processing Summary

		N	Percent
Cases available in analysis	Event[a]	69	75.8%
	Censored	12	13.2%
	Total	81	89.0%
Cases dropped	Cases with missing values	10	11.0%
	Cases with negative time	0	0.0%
	Censored cases before the earliest event in a stratum	0	0.0%
	Total	10	11.0%
Total		91	100.0%

a. Dependent Variable: Survival_month

Block 0: Beginning Block

Omnibus Tests of Model Coefficients

-2 Log Likelihood
515.861

Block 1: Method = Enter

Omnibus Tests of Model Coefficients[a]

-2 Log Likelihood	Overall (score)			Change From Previous Step			Change From Previous Block		
	Chi-square	df	Sig.	Chi-square	df	Sig.	Chi-square	df	Sig.
515.392	.473	1	.492	.469	1	.493	.469	1	.493

a. Beginning Block Number 1. Method = Enter

Variables in the Equation

	B	SE	Wald	df	Sig.	Exp(B)	95.0% CI for Exp(B)	
							Lower	Upper
sex	.167	.243	.471	1	.492	1.181	.734	1.901

Covariate Means

	Mean
sex	.469

```
COXREG Survival_month
/STATUS=Death(1)
/METHOD=ENTER age
/PRINT=CI(95)SUMMARY
/CRITERIA=PIN(.05)POUT(.10)ITERATE(20).
```

Cox Regression

Notes

Output Created		25-JAN-2024 08:54:59
Comments		
Input	Data	D:\D\project\IL6 and BTC\manuscript\frontiers in immunology\Expression and prognosis.sav
	Active Dataset	1
	Filter	<none>
	Weight	<none>
	Split File	<none>
	N of Rows in Working Data File	91
Missing Value Handling	Definition of Missing	User-defined missing values are treated as missing.
Syntax		COXREG Survival_month /STATUS=Death(1) /METHOD=ENTER age /PRINT=CI(95) SUMMARY /CRITERIA=PIN(.05) POUT(.10) ITERATE(20).
Resources	Processor Time	00:00:00.00
	Elapsed Time	00:00:00.01

Case Processing Summary

		N	Percent
Cases available in analysis	Event[a]	69	75.8%
	Censored	12	13.2%
	Total	81	89.0%
Cases dropped	Cases with missing values	10	11.0%
	Cases with negative time	0	0.0%
	Censored cases before the earliest event in a stratum	0	0.0%
	Total	10	11.0%
Total		91	100.0%

a. Dependent Variable: Survival_month

Block 0: Beginning Block

Omnibus Tests of Model Coefficients

-2 Log Likelihood
515.861

Block 1: Method = Enter

Omnibus Tests of Model Coefficients[a]

-2 Log Likelihood	Overall (score)			Change From Previous Step			Change From Previous Block		
	Chi-square	df	Sig.	Chi-square	df	Sig.	Chi-square	df	Sig.
515.719	.141	1	.707	.142	1	.706	.142	1	.706

a. Beginning Block Number 1. Method = Enter

Variables in the Equation

	B	SE	Wald	df	Sig.	Exp(B)	95.0% CI for Exp(B)	
							Lower	Upper
age	.004	.012	.141	1	.707	1.004	.981	1.028

Covariate Means

	Mean
age	56.790

```
COXREG Survival_month
/STATUS=Death(1)
/METHOD=ENTER wbc
/PRINT=CI(95)SUMMARY
/CRITERIA=PIN(.05)POUT(.10)ITERATE(20).
```

Cox Regression

Notes

Output Created		25-JAN-2024 08:55:09
Comments		
Input	Data	D:\D\project\IL6 and BTC\manuscript\frontiers in immunology\Expression and prognosis.sav
	Active Dataset	1
	Filter	<none>
	Weight	<none>
	Split File	<none>
	N of Rows in Working Data File	91
Missing Value Handling	Definition of Missing	User-defined missing values are treated as missing.
Syntax		COXREG Survival_month /STATUS=Death(1) /METHOD=ENTER wbc /PRINT=CI(95) SUMMARY /CRITERIA=PIN(.05) POUT(.10) ITERATE(20).
Resources	Processor Time	00:00:00.00
	Elapsed Time	00:00:00.01

Case Processing Summary

		N	Percent
Cases available in analysis	Event[a]	69	75.8%
	Censored	12	13.2%
	Total	81	89.0%
Cases dropped	Cases with missing values	10	11.0%
	Cases with negative time	0	0.0%
	Censored cases before the earliest event in a stratum	0	0.0%
	Total	10	11.0%
Total		91	100.0%

a. Dependent Variable: Survival_month

Block 0: Beginning Block

Omnibus Tests of Model Coefficients

-2 Log Likelihood
515.861

Block 1: Method = Enter

Omnibus Tests of Model Coefficients[a]

-2 Log Likelihood	Overall (score)			Change From Previous Step			Change From Previous Block		
	Chi-square	df	Sig.	Chi-square	df	Sig.	Chi-square	df	Sig.
513.479	2.686	1	.101	2.382	1	.123	2.382	1	.123

a. Beginning Block Number 1. Method = Enter

Variables in the Equation

	B	SE	Wald	df	Sig.	Exp(B)	95.0% CI for Exp(B)	
							Lower	Upper
wbc	.067	.041	2.679	1	.102	1.069	.987	1.158

Covariate Means

	Mean
wbc	7.282

```
COXREG Survival_month
/STATUS=Death(1)
/METHOD=ENTER Neutrophils
/PRINT=CI(95)SUMMARY
/CRITERIA=PIN(.05)POUT(.10)ITERATE(20).
```

Cox Regression

Notes

Output Created		25-JAN-2024 08:55:18
Comments		
Input	Data	D:\D\project\IL6 and BTC\manuscript\frontiers in immunology\Expression and prognosis.sav
	Active Dataset	1
	Filter	<none>
	Weight	<none>
	Split File	<none>
	N of Rows in Working Data File	91
Missing Value Handling	Definition of Missing	User-defined missing values are treated as missing.
Syntax		COXREG Survival_month /STATUS=Death(1) /METHOD=ENTER Neutrophils /PRINT=CI(95) SUMMARY /CRITERIA=PIN(.05) POUT(.10) ITERATE(20).
Resources	Processor Time	00:00:00.02
	Elapsed Time	00:00:00.01

Case Processing Summary

		N	Percent
Cases available in analysis	Event[a]	69	75.8%
	Censored	12	13.2%
	Total	81	89.0%
Cases dropped	Cases with missing values	10	11.0%
	Cases with negative time	0	0.0%
	Censored cases before the earliest event in a stratum	0	0.0%
	Total	10	11.0%
Total		91	100.0%

a. Dependent Variable: Survival_month

Block 0: Beginning Block

Omnibus Tests of Model Coefficients

-2 Log Likelihood
515.861

Block 1: Method = Enter

Omnibus Tests of Model Coefficients[a]

-2 Log Likelihood	Overall (score)			Change From Previous Step			Change From Previous Block		
	Chi-square	df	Sig.	Chi-square	df	Sig.	Chi-square	df	Sig.
514.069	1.724	1	.189	1.792	1	.181	1.792	1	.181

a. Beginning Block Number 1. Method = Enter

Variables in the Equation

	B	SE	Wald	df	Sig.	Exp(B)	95.0% CI for Exp(B)	
							Lower	Upper
Neutrophils	.014	.011	1.744	1	.187	1.014	.993	1.036

Covariate Means

	Mean
Neutrophils	67.015

```
COXREG Survival_month
/STATUS=Death(1)
/METHOD=ENTER lymphocyte
/PRINT=CI(95)SUMMARY
/CRITERIA=PIN(.05)POUT(.10)ITERATE(20).
```

Cox Regression

Notes

Output Created		25-JAN-2024 08:55:25
Comments		
Input	Data	D:\D\project\IL6 and BTC\manuscript\frontiers in immunology\Expression and prognosis.sav
	Active Dataset	1
	Filter	<none>
	Weight	<none>
	Split File	<none>
	N of Rows in Working Data File	91
Missing Value Handling	Definition of Missing	User-defined missing values are treated as missing.
Syntax		COXREG Survival_month<br>/STATUS=Death(1)<br>/METHOD=ENTER lymphocyte<br>/PRINT=CI(95) SUMMARY<br>/CRITERIA=PIN(.05) POUT(.10) ITERATE(20).
Resources	Processor Time	00:00:00.02
	Elapsed Time	00:00:00.01

Case Processing Summary

		N	Percent
Cases available in analysis	Event[a]	69	75.8%
	Censored	12	13.2%
	Total	81	89.0%
Cases dropped	Cases with missing values	10	11.0%
	Cases with negative time	0	0.0%
	Censored cases before the earliest event in a stratum	0	0.0%
	Total	10	11.0%
Total		91	100.0%

a. Dependent Variable: Survival_month

Block 0: Beginning Block

Omnibus Tests of Model Coefficients

-2 Log Likelihood
515.861

Block 1: Method = Enter

Omnibus Tests of Model Coefficients[a]

-2 Log Likelihood	Overall (score)			Change From Previous Step			Change From Previous Block		
	Chi-square	df	Sig.	Chi-square	df	Sig.	Chi-square	df	Sig.
513.804	2.028	1	.154	2.057	1	.152	2.057	1	.152

a. Beginning Block Number 1. Method = Enter

Variables in the Equation

	B	SE	Wald	df	Sig.	Exp(B)	95.0% CI for Exp(B)	
							Lower	Upper
lymphocyte	-.020	.014	2.026	1	.155	.980	.953	1.008

Covariate Means

	Mean
lymphocyte	21.595

```
COXREG Survival_month
/STATUS=Death(1)
/METHOD=ENTER hemoglobin
/PRINT=CI(95)SUMMARY
/CRITERIA=PIN(.05)POUT(.10)ITERATE(20).
```

Cox Regression

Notes

Output Created		25-JAN-2024 08:55:32
Comments		
Input	Data	D:\D\project\IL6 and BTC\manuscript\frontiers in immunology\Expression and prognosis.sav
	Active Dataset	1
	Filter	<none>
	Weight	<none>
	Split File	<none>
	N of Rows in Working Data File	91
Missing Value Handling	Definition of Missing	User-defined missing values are treated as missing.
Syntax		COXREG Survival_month /STATUS=Death(1) /METHOD=ENTER hemoglobin /PRINT=CI(95) SUMMARY /CRITERIA=PIN(.05) POUT(.10) ITERATE(20).
Resources	Processor Time	00:00:00.00
	Elapsed Time	00:00:00.01

Case Processing Summary

		N	Percent
Cases available in analysis	Event[a]	69	75.8%
	Censored	12	13.2%
	Total	81	89.0%
Cases dropped	Cases with missing values	10	11.0%
	Cases with negative time	0	0.0%
	Censored cases before the earliest event in a stratum	0	0.0%
	Total	10	11.0%
Total		91	100.0%

a. Dependent Variable: Survival_month

Block 0: Beginning Block

Omnibus Tests of Model Coefficients

-2 Log Likelihood
515.861

Block 1: Method = Enter

Omnibus Tests of Model Coefficients[a]

-2 Log Likelihood	Overall (score)			Change From Previous Step			Change From Previous Block		
	Chi-square	df	Sig.	Chi-square	df	Sig.	Chi-square	df	Sig.
515.784	.078	1	.780	.077	1	.782	.077	1	.782

a. Beginning Block Number 1. Method = Enter

Variables in the Equation

	B	SE	Wald	df	Sig.	Exp(B)	95.0% CI for Exp(B)	
							Lower	Upper
hemoglobin	-.002	.007	.078	1	.780	.998	.985	1.011

Covariate Means

	Mean
hemoglobin	119.457

```
COXREG Survival_month
/STATUS=Death(1)
/METHOD=ENTER plt
/PRINT=CI(95)SUMMARY
/CRITERIA=PIN(.05)POUT(.10)ITERATE(20).
```

Cox Regression

Notes

Output Created		25-JAN-2024 08:55:41
Comments		
Input	Data	D:\D\project\IL6 and BTC\manuscript\frontiers in immunology\Expression and prognosis.sav
	Active Dataset	1
	Filter	<none>
	Weight	<none>
	Split File	<none>
	N of Rows in Working Data File	91
Missing Value Handling	Definition of Missing	User-defined missing values are treated as missing.
Syntax		COXREG Survival_month<br>/STATUS=Death(1)<br>/METHOD=ENTER plt<br>/PRINT=CI(95) SUMMARY<br>/CRITERIA=PIN(.05) POUT(.10) ITERATE(20).
Resources	Processor Time	00:00:00.02
	Elapsed Time	00:00:00.01

Case Processing Summary

		N	Percent
Cases available in analysis	Event[a]	69	75.8%
	Censored	12	13.2%
	Total	81	89.0%
Cases dropped	Cases with missing values	10	11.0%
	Cases with negative time	0	0.0%
	Censored cases before the earliest event in a stratum	0	0.0%
	Total	10	11.0%
Total		91	100.0%

a. Dependent Variable: Survival_month

Block 0: Beginning Block

Omnibus Tests of Model Coefficients

-2 Log Likelihood
515.861

Block 1: Method = Enter

Omnibus Tests of Model Coefficients[a]

-2 Log Likelihood	Overall (score)			Change From Previous Step			Change From Previous Block		
	Chi-square	df	Sig.	Chi-square	df	Sig.	Chi-square	df	Sig.
511.866	3.844	1	.050	3.995	1	.046	3.995	1	.046

a. Beginning Block Number 1. Method = Enter

Variables in the Equation

	B	SE	Wald	df	Sig.	Exp(B)	95.0% CI for Exp(B)	
							Lower	Upper
plt	-.003	.002	3.835	1	.050	.997	.993	1.000

Covariate Means

	Mean
plt	239.185

```
COXREG Survival_month
/STATUS=Death(1)
/METHOD=ENTER CA12_5
/PRINT=CI(95)SUMMARY
/CRITERIA=PIN(.05)POUT(.10)ITERATE(20).
```

Cox Regression

Notes

Output Created		25-JAN-2024 08:55:52
Comments		
Input	Data	D:\D\project\IL6 and BTC\manuscript\frontiers in immunology\Expression and prognosis.sav
	Active Dataset	1
	Filter	<none>
	Weight	<none>
	Split File	<none>
	N of Rows in Working Data File	91
Missing Value Handling	Definition of Missing	User-defined missing values are treated as missing.
Syntax		COXREG Survival_month /STATUS=Death(1) /METHOD=ENTER CA12_5 /PRINT=CI(95) SUMMARY /CRITERIA=PIN(.05) POUT(.10) ITERATE(20).
Resources	Processor Time	00:00:00.02
	Elapsed Time	00:00:00.01

Case Processing Summary

		N	Percent
Cases available in analysis	Event[a]	67	73.6%
	Censored	12	13.2%
	Total	79	86.8%
Cases dropped	Cases with missing values	12	13.2%
	Cases with negative time	0	0.0%
	Censored cases before the earliest event in a stratum	0	0.0%
	Total	12	13.2%
Total		91	100.0%

a. Dependent Variable: Survival_month

Block 0: Beginning Block

Omnibus Tests of Model Coefficients

-2 Log Likelihood
498.249

Block 1: Method = Enter

Omnibus Tests of Model Coefficients[a]

-2 Log Likelihood	Overall (score)			Change From Previous Step			Change From Previous Block		
	Chi-square	df	Sig.	Chi-square	df	Sig.	Chi-square	df	Sig.
494.365	5.823	1	.016	3.884	1	.049	3.884	1	.049

a. Beginning Block Number 1. Method = Enter

Variables in the Equation

	B	SE	Wald	df	Sig.	Exp(B)	95.0% CI for Exp(B)	
							Lower	Upper
CA12_5	.003	.001	5.288	1	.021	1.003	1.000	1.006

Covariate Means

	Mean
CA12_5	38.166

```
COXREG Survival_month
/STATUS=Death(1)
/METHOD=ENTER CA19_9
/PRINT=CI(95)SUMMARY
/CRITERIA=PIN(.05)POUT(.10)ITERATE(20).
```

Cox Regression

Notes

Output Created		25-JAN-2024 08:55:59
Comments		
Input	Data	D:\D\project\IL6 and BTC\manuscript\frontiers in immunology\Expression and prognosis.sav
	Active Dataset	1
	Filter	<none>
	Weight	<none>
	Split File	<none>
	N of Rows in Working Data File	91
Missing Value Handling	Definition of Missing	User-defined missing values are treated as missing.
Syntax		COXREG Survival_month /STATUS=Death(1) /METHOD=ENTER CA19_9 /PRINT=CI(95) SUMMARY /CRITERIA=PIN(.05) POUT(.10) ITERATE(20).
Resources	Processor Time	00:00:00.00
	Elapsed Time	00:00:00.02

Case Processing Summary

		N	Percent
Cases available in analysis	Event[a]	63	69.2%
	Censored	11	12.1%
	Total	74	81.3%
Cases dropped	Cases with missing values	17	18.7%
	Cases with negative time	0	0.0%
	Censored cases before the earliest event in a stratum	0	0.0%
	Total	17	18.7%
Total		91	100.0%

a. Dependent Variable: Survival_month

Block 0: Beginning Block

Omnibus Tests of Model Coefficients

-2 Log Likelihood
460.313

Block 1: Method = Enter

Omnibus Tests of Model Coefficients[a]

-2 Log Likelihood	Overall (score)			Change From Previous Step			Change From Previous Block		
	Chi-square	df	Sig.	Chi-square	df	Sig.	Chi-square	df	Sig.
457.856	2.644	1	.104	2.457	1	.117	2.457	1	.117

a. Beginning Block Number 1. Method = Enter

Variables in the Equation

	B	SE	Wald	df	Sig.	Exp(B)	95.0% CI for Exp(B)	
							Lower	Upper
CA19_9	.001	.000	2.616	1	.106	1.001	1.000	1.002

Covariate Means

	Mean
CA19_9	253.340

```
COXREG Survival_month
/STATUS = Death(1)
/METHOD= ENTER CEA
/PRINT= CI(95) SUMMARY
/CRITERIA= PIN(.05) POUT(.10) ITERATE(20).
```

Cox Regression

Notes

Output Created		25-JAN-2024 08:56:08
Comments		
Input	Data	D:\D\project\IL6 and BTC\manuscript\frontiers in immunology\Expression and prognosis.sav
	Active Dataset	1
	Filter	<none>
	Weight	<none>
	Split File	<none>
	N of Rows in Working Data File	91
Missing Value Handling	Definition of Missing	User-defined missing values are treated as missing.
Syntax		COXREG Survival_month /STATUS=Death(1) /METHOD=ENTER CEA /PRINT=CI(95) SUMMARY /CRITERIA=PIN(.05) POUT(.10) ITERATE(20).
Resources	Processor Time	00:00:00.00
	Elapsed Time	00:00:00.01

Case Processing Summary

		N	Percent
Cases available in analysis	Event[a]	69	75.8%
	Censored	12	13.2%
	Total	81	89.0%
Cases dropped	Cases with missing values	10	11.0%
	Cases with negative time	0	0.0%
	Censored cases before the earliest event in a stratum	0	0.0%
	Total	10	11.0%
Total		91	100.0%

a. Dependent Variable: Survival_month

Block 0: Beginning Block

Omnibus Tests of Model Coefficients

-2 Log Likelihood
515.861

Block 1: Method = Enter

Omnibus Tests of Model Coefficients[a]

-2 Log Likelihood	Overall (score)			Change From Previous Step			Change From Previous Block		
	Chi-square	df	Sig.	Chi-square	df	Sig.	Chi-square	df	Sig.
513.822	3.551	1	.060	2.039	1	.153	2.039	1	.153

a. Beginning Block Number 1. Method = Enter

Variables in the Equation

	B	SE	Wald	df	Sig.	Exp(B)	95.0% CI for Exp(B)	
							Lower	Upper
CEA	.004	.003	3.023	1	.082	1.004	.999	1.009

Covariate Means

	Mean
CEA	9.854

```
COXREG Survival_month
/STATUS=Death(1)
/METHOD=ENTER CA24_2
/PRINT=CI(95)SUMMARY
/CRITERIA=PIN(.05)POUT(.10)ITERATE(20).
```

Cox Regression

Notes

Output Created		25-JAN-2024 08:56:16
Comments		
Input	Data	D:\D\project\IL6 and BTC\manuscript\frontiers in immunology\Expression and prognosis.sav
	Active Dataset	1
	Filter	<none>
	Weight	<none>
	Split File	<none>
	N of Rows in Working Data File	91
Missing Value Handling	Definition of Missing	User-defined missing values are treated as missing.
Syntax		COXREG Survival_month /STATUS=Death(1) /METHOD=ENTER CA24_2 /PRINT=CI(95) SUMMARY /CRITERIA=PIN(.05) POUT(.10) ITERATE(20).
Resources	Processor Time	00:00:00.00
	Elapsed Time	00:00:00.00

Case Processing Summary

		N	Percent
Cases available in analysis	Event[a]	69	75.8%
	Censored	12	13.2%
	Total	81	89.0%
Cases dropped	Cases with missing values	10	11.0%
	Cases with negative time	0	0.0%
	Censored cases before the earliest event in a stratum	0	0.0%
	Total	10	11.0%
Total		91	100.0%

a. Dependent Variable: Survival_month

Block 0: Beginning Block

Omnibus Tests of Model Coefficients

-2 Log Likelihood
515.861

Block 1: Method = Enter

Omnibus Tests of Model Coefficients[a]

-2 Log Likelihood	Overall (score)			Change From Previous Step			Change From Previous Block		
	Chi-square	df	Sig.	Chi-square	df	Sig.	Chi-square	df	Sig.
512.963	3.366	1	.067	2.899	1	.089	2.899	1	.089

a. Beginning Block Number 1. Method = Enter

Variables in the Equation

	B	SE	Wald	df	Sig.	Exp(B)	95.0% CI for Exp(B)	
							Lower	Upper
CA24_2	.002	.001	3.287	1	.070	1.002	1.000	1.003

Covariate Means

	Mean
CA24_2	88.358

```
COXREG Survival_month
/STATUS=Death(1)
/METHOD=ENTER AFP
/PRINT=CI(95)SUMMARY
/CRITERIA=PIN(.05)POUT(.10)ITERATE(20).
```

Cox Regression

Notes

Output Created		25-JAN-2024 08:56:24
Comments		
Input	Data	D:\D\project\IL6 and BTC\manuscript\frontiers in immunology\Expression and prognosis.sav
	Active Dataset	1
	Filter	<none>
	Weight	<none>
	Split File	<none>
	N of Rows in Working Data File	91
Missing Value Handling	Definition of Missing	User-defined missing values are treated as missing.
Syntax		COXREG Survival_month /STATUS=Death(1) /METHOD=ENTER AFP /PRINT=CI(95) SUMMARY /CRITERIA=PIN(.05) POUT(.10) ITERATE(20).
Resources	Processor Time	00:00:00.00
	Elapsed Time	00:00:00.01

Case Processing Summary

		N	Percent
Cases available in analysis	Event[a]	69	75.8%
	Censored	12	13.2%
	Total	81	89.0%
Cases dropped	Cases with missing values	10	11.0%
	Cases with negative time	0	0.0%
	Censored cases before the earliest event in a stratum	0	0.0%
	Total	10	11.0%
Total		91	100.0%

a. Dependent Variable: Survival_month

Block 0: Beginning Block

Omnibus Tests of Model Coefficients

-2 Log Likelihood
515.861

Block 1: Method = Enter

Omnibus Tests of Model Coefficients[a]

-2 Log Likelihood	Overall (score)			Change From Previous Step			Change From Previous Block		
	Chi-square	df	Sig.	Chi-square	df	Sig.	Chi-square	df	Sig.
515.494	.383	1	.536	.368	1	.544	.368	1	.544

a. Beginning Block Number 1. Method = Enter

Variables in the Equation

	B	SE	Wald	df	Sig.	Exp(B)	95.0% CI for Exp(B)	
							Lower	Upper
AFP	.013	.021	.382	1	.536	1.013	.973	1.055

Covariate Means

	Mean
AFP	6.284

```
COXREG Survival_month
/STATUS=Death(1)
/METHOD=ENTER HBsAg
/PRINT=CI(95)SUMMARY
/CRITERIA=PIN(.05)POUT(.10)ITERATE(20).
```

Cox Regression

Notes

Output Created		25-JAN-2024 08:56:31
Comments		
Input	Data	D:\D\project\IL6 and BTC\manuscript\frontiers in immunology\Expression and prognosis.sav
	Active Dataset	1
	Filter	<none>
	Weight	<none>
	Split File	<none>
	N of Rows in Working Data File	91
Missing Value Handling	Definition of Missing	User-defined missing values are treated as missing.
Syntax		COXREG Survival_month /STATUS=Death(1) /METHOD=ENTER HBsAg /PRINT=CI(95) SUMMARY /CRITERIA=PIN(.05) POUT(.10) ITERATE(20).
Resources	Processor Time	00:00:00.02
	Elapsed Time	00:00:00.01

Case Processing Summary

		N	Percent
Cases available in analysis	Event[a]	69	75.8%
	Censored	12	13.2%
	Total	81	89.0%
Cases dropped	Cases with missing values	10	11.0%
	Cases with negative time	0	0.0%
	Censored cases before the earliest event in a stratum	0	0.0%
	Total	10	11.0%
Total		91	100.0%

a. Dependent Variable: Survival_month

Block 0: Beginning Block

Omnibus Tests of Model Coefficients

-2 Log Likelihood
515.861

Block 1: Method = Enter

Omnibus Tests of Model Coefficients[a]

-2 Log Likelihood	Overall (score)			Change From Previous Step			Change From Previous Block		
	Chi-square	df	Sig.	Chi-square	df	Sig.	Chi-square	df	Sig.
515.756	.110	1	.740	.106	1	.745	.106	1	.745

a. Beginning Block Number 1. Method = Enter

Variables in the Equation

	B	SE	Wald	df	Sig.	Exp(B)	95.0% CI for Exp(B)	
							Lower	Upper
HBsAg	.142	.428	.110	1	.740	1.153	.498	2.669

Covariate Means

	Mean
HBsAg	.086

```
COXREG Survival_month
/STATUS=Death(1)
/METHOD=ENTER Bismuth
/PRINT=CI(95)SUMMARY
/CRITERIA=PIN(.05)POUT(.10)ITERATE(20).
```

Cox Regression

Notes

Output Created		25-JAN-2024 08:56:48
Comments		
Input	Data	D:\D\project\IL6 and BTC\manuscript\frontiers in immunology\Expression and prognosis.sav
	Active Dataset	1
	Filter	<none>
	Weight	<none>
	Split File	<none>
	N of Rows in Working Data File	91
Missing Value Handling	Definition of Missing	User-defined missing values are treated as missing.
Syntax		COXREG Survival_month /STATUS=Death(1) /METHOD=ENTER Bismuth /PRINT=CI(95) SUMMARY /CRITERIA=PIN(.05) POUT(.10) ITERATE(20).
Resources	Processor Time	00:00:00.00
	Elapsed Time	00:00:00.00

Case Processing Summary

		N	Percent
Cases available in analysis	Event[a]	69	75.8%
	Censored	12	13.2%
	Total	81	89.0%
Cases dropped	Cases with missing values	10	11.0%
	Cases with negative time	0	0.0%
	Censored cases before the earliest event in a stratum	0	0.0%
	Total	10	11.0%
Total		91	100.0%

a. Dependent Variable: Survival_month

Block 0: Beginning Block

Omnibus Tests of Model Coefficients

-2 Log Likelihood
515.861

Block 1: Method = Enter

Omnibus Tests of Model Coefficients[a]

-2 Log Likelihood	Overall (score)			Change From Previous Step			Change From Previous Block		
	Chi-square	df	Sig.	Chi-square	df	Sig.	Chi-square	df	Sig.
515.852	.010	1	.922	.010	1	.922	.010	1	.922

a. Beginning Block Number 1. Method = Enter

Variables in the Equation

	B	SE	Wald	df	Sig.	Exp(B)	95.0% CI for Exp(B)	
							Lower	Upper
Bismuth	-.017	.168	.010	1	.922	.984	.708	1.367

Covariate Means

	Mean
Bismuth	2.704

```
COXREG Survival_month
/STATUS=Death(1)
/METHOD=ENTER tumor_differentiation
/PRINT=CI(95)SUMMARY
/CRITERIA=PIN(.05)POUT(.10)ITERATE(20).
```

Cox Regression

Notes

Output Created		25-JAN-2024 08:56:58
Comments		
Input	Data	D:\D\project\IL6 and BTC\manuscript\frontiers in immunology\Expression and prognosis.sav
	Active Dataset	1
	Filter	<none>
	Weight	<none>
	Split File	<none>
	N of Rows in Working Data File	91
Missing Value Handling	Definition of Missing	User-defined missing values are treated as missing.
Syntax		COXREG Survival_month /STATUS=Death(1) /METHOD=ENTER tumor_differentiation /PRINT=CI(95) SUMMARY /CRITERIA=PIN(.05) POUT(.10) ITERATE(20).
Resources	Processor Time	00:00:00.03
	Elapsed Time	00:00:00.01

Case Processing Summary

		N	Percent
Cases available in analysis	Event[a]	69	75.8%
	Censored	11	12.1%
	Total	80	87.9%
Cases dropped	Cases with missing values	11	12.1%
	Cases with negative time	0	0.0%
	Censored cases before the earliest event in a stratum	0	0.0%
	Total	11	12.1%
Total		91	100.0%

a. Dependent Variable: Survival_month

Block 0: Beginning Block

Omnibus Tests of Model Coefficients

-2 Log Likelihood
513.120

Block 1: Method = Enter

Omnibus Tests of Model Coefficients[a]

-2 Log Likelihood	Overall (score)			Change From Previous Step			Change From Previous Block		
	Chi-square	df	Sig.	Chi-square	df	Sig.	Chi-square	df	Sig.
508.521	4.635	1	.031	4.598	1	.032	4.598	1	.032

a. Beginning Block Number 1. Method = Enter

Variables in the Equation

	B	SE	Wald	df	Sig.	Exp(B)	95.0% CI for Exp(B)	
							Lower	Upper
tumor_differentiation	-.502	.232	4.656	1	.031	.606	.384	.955

Covariate Means

	Mean
tumor_differentiation	1.888

```
COXREG Survival_month
/STATUS=Death(1)
/METHOD=ENTER Portal_lymphnode
/PRINT=CI(95)SUMMARY
/CRITERIA=PIN(.05)POUT(.10)ITERATE(20).
```

Cox Regression

Notes

Output Created		25-JAN-2024 08:57:08
Comments		
Input	Data	D:\D\project\IL6 and BTC\manuscript\frontiers in immunology\Expression and prognosis.sav
	Active Dataset	1
	Filter	<none>
	Weight	<none>
	Split File	<none>
	N of Rows in Working Data File	91
Missing Value Handling	Definition of Missing	User-defined missing values are treated as missing.
Syntax		COXREG Survival_month /STATUS=Death(1) /METHOD=ENTER Portal_lymphnode /PRINT=CI(95) SUMMARY /CRITERIA=PIN(.05) POUT(.10) ITERATE(20).
Resources	Processor Time	00:00:00.00
	Elapsed Time	00:00:00.01

Case Processing Summary

		N	Percent
Cases available in analysis	Event[a]	69	75.8%
	Censored	12	13.2%
	Total	81	89.0%
Cases dropped	Cases with missing values	10	11.0%
	Cases with negative time	0	0.0%
	Censored cases before the earliest event in a stratum	0	0.0%
	Total	10	11.0%
Total		91	100.0%

a. Dependent Variable: Survival_month

Block 0: Beginning Block

Omnibus Tests of Model Coefficients

-2 Log Likelihood
515.861

Block 1: Method = Enter

Omnibus Tests of Model Coefficients[a]

-2 Log Likelihood	Overall (score)			Change From Previous Step			Change From Previous Block		
	Chi-square	df	Sig.	Chi-square	df	Sig.	Chi-square	df	Sig.
511.683	4.545	1	.033	4.179	1	.041	4.179	1	.041

a. Beginning Block Number 1. Method = Enter

Variables in the Equation

	B	SE	Wald	df	Sig.	Exp(B)	95.0% CI for Exp(B)	
							Lower	Upper
Portal_lymphnode	.538	.255	4.441	1	.035	1.713	1.038	2.826

Covariate Means

	Mean
Portal_lymphnode	.321

```
COXREG Survival_month
/STATUS=Death(1)
/METHOD=ENTER Distant_lymphnode_metastasis
/PRINT=CI(95)SUMMARY
/CRITERIA=PIN(.05)POUT(.10)ITERATE(20).
```

Cox Regression

Notes

Output Created		25-JAN-2024 08:57:17
Comments		
Input	Data	D:\D\project\IL6 and BTC\manuscript\frontiers in immunology\Expression and prognosis.sav
	Active Dataset	1
	Filter	<none>
	Weight	<none>
	Split File	<none>
	N of Rows in Working Data File	91
Missing Value Handling	Definition of Missing	User-defined missing values are treated as missing.
Syntax		COXREG Survival_month /STATUS=Death(1) /METHOD=ENTER Distant_lymphnode_metastasis /PRINT=CI(95) SUMMARY /CRITERIA=PIN(.05) POUT(.10) ITERATE(20).
Resources	Processor Time	00:00:00.00
	Elapsed Time	00:00:00.01

Case Processing Summary

		N	Percent
Cases available in analysis	Event[a]	69	75.8%
	Censored	12	13.2%
	Total	81	89.0%
Cases dropped	Cases with missing values	10	11.0%
	Cases with negative time	0	0.0%
	Censored cases before the earliest event in a stratum	0	0.0%
	Total	10	11.0%
Total		91	100.0%

a. Dependent Variable: Survival_month

Block 0: Beginning Block

Omnibus Tests of Model Coefficients

-2 Log Likelihood
515.861

Block 1: Method = Enter

Omnibus Tests of Model Coefficients[a]

-2 Log Likelihood	Overall (score)			Change From Previous Step			Change From Previous Block		
	Chi-square	df	Sig.	Chi-square	df	Sig.	Chi-square	df	Sig.
513.850	2.345	1	.126	2.011	1	.156	2.011	1	.156

a. Beginning Block Number 1. Method = Enter

Variables in the Equation

	B	SE	Wald	df	Sig.	Exp(B)	95.0% CI for Exp(B)	
							Lower	Upper
Distant_lymphnode_metastasis	.547	.362	2.289	1	.130	1.728	.851	3.512

Covariate Means

	Mean
Distant_lymphnode_metastasis	.123

```
COXREG Survival_month
/STATUS=Death(1)
/METHOD=ENTER Vascular_invasion
/PRINT=CI(95)SUMMARY
/CRITERIA=PIN(.05)POUT(.10)ITERATE(20).
```

Cox Regression

Notes

Output Created		25-JAN-2024 08:57:24
Comments		
Input	Data	D:\D\project\IL6 and BTC\manuscript\frontiers in immunology\Expression and prognosis.sav
	Active Dataset	1
	Filter	<none>
	Weight	<none>
	Split File	<none>
	N of Rows in Working Data File	91
Missing Value Handling	Definition of Missing	User-defined missing values are treated as missing.
Syntax		COXREG Survival_month /STATUS=Death(1) /METHOD=ENTER Vascular_invasion /PRINT=CI(95) SUMMARY /CRITERIA=PIN(.05) POUT(.10) ITERATE(20).
Resources	Processor Time	00:00:00.02
	Elapsed Time	00:00:00.01

Case Processing Summary

		N	Percent
Cases available in analysis	Event[a]	69	75.8%
	Censored	12	13.2%
	Total	81	89.0%
Cases dropped	Cases with missing values	10	11.0%
	Cases with negative time	0	0.0%
	Censored cases before the earliest event in a stratum	0	0.0%
	Total	10	11.0%
Total		91	100.0%

a. Dependent Variable: Survival_month

Block 0: Beginning Block

Omnibus Tests of Model Coefficients

-2 Log Likelihood
515.861

Block 1: Method = Enter

Omnibus Tests of Model Coefficients[a]

-2 Log Likelihood	Overall (score)			Change From Previous Step			Change From Previous Block		
	Chi-square	df	Sig.	Chi-square	df	Sig.	Chi-square	df	Sig.
509.594	7.057	1	.008	6.267	1	.012	6.267	1	.012

a. Beginning Block Number 1. Method = Enter

Variables in the Equation

	B	SE	Wald	df	Sig.	Exp(B)	95.0% CI for Exp(B)	
							Lower	Upper
Vascular_invasion	.678	.260	6.809	1	.009	1.969	1.184	3.276

Covariate Means

	Mean
Vascular_invasion	.309

```
COXREG Survival_month
/STATUS=Death(1)
/METHOD=ENTER Perineural_invasion
/PRINT=CI(95)SUMMARY
/CRITERIA=PIN(.05)POUT(.10)ITERATE(20).
```

Cox Regression

Notes

Output Created		25-JAN-2024 08:57:34
Comments		
Input	Data	D:\D\project\IL6 and BTC\manuscript\frontiers in immunology\Expression and prognosis.sav
	Active Dataset	1
	Filter	<none>
	Weight	<none>
	Split File	<none>
	N of Rows in Working Data File	91
Missing Value Handling	Definition of Missing	User-defined missing values are treated as missing.
Syntax		COXREG Survival_month /STATUS=Death(1) /METHOD=ENTER Perineural_invasion /PRINT=CI(95) SUMMARY /CRITERIA=PIN(.05) POUT(.10) ITERATE(20).
Resources	Processor Time	00:00:00.00
	Elapsed Time	00:00:00.01

Case Processing Summary

		N	Percent
Cases available in analysis	Event[a]	69	75.8%
	Censored	12	13.2%
	Total	81	89.0%
Cases dropped	Cases with missing values	10	11.0%
	Cases with negative time	0	0.0%
	Censored cases before the earliest event in a stratum	0	0.0%
	Total	10	11.0%
Total		91	100.0%

a. Dependent Variable: Survival_month

Block 0: Beginning Block

Omnibus Tests of Model Coefficients

-2 Log Likelihood
515.861

Block 1: Method = Enter

Omnibus Tests of Model Coefficients[a]

-2 Log Likelihood	Overall (score)			Change From Previous Step			Change From Previous Block		
	Chi-square	df	Sig.	Chi-square	df	Sig.	Chi-square	df	Sig.
515.833	.028	1	.868	.028	1	.867	.028	1	.867

a. Beginning Block Number 1. Method = Enter

Variables in the Equation

	B	SE	Wald	df	Sig.	Exp(B)	95.0% CI for Exp(B)	
							Lower	Upper
Perineural_invasion	-.028	.168	.028	1	.868	.972	.699	1.352

Covariate Means

	Mean
Perineural_invasion	.321

```
COXREG Survival_month
/STATUS=Death(1)
/METHOD=ENTER Vascular_invasion Portal_lymphnode tumor_differentiation CA24_2
CEA CA12_5 plt
/PRINT=CI(95)SUMMARY
/CRITERIA=PIN(.05)POUT(.10)ITERATE(20).
```

Cox Regression

Notes

Output Created		25-JAN-2024 08:58:54
Comments		
Input	Data	D:\D\project\IL6 and BTC\manuscript\frontiers in immunology\Expression and prognosis.sav
	Active Dataset	1
	Filter	<none>
	Weight	<none>
	Split File	<none>
	N of Rows in Working Data File	91
Missing Value Handling	Definition of Missing	User-defined missing values are treated as missing.
Syntax		COXREG Survival_month /STATUS=Death(1) /METHOD=ENTER Vascular_invasion Portal_lymphnode tumor_differentiation CA24_2 CEA CA12_5 plt /PRINT=CI(95) SUMMARY /CRITERIA=PIN(.05) POUT(.10) ITERATE(20).
Resources	Processor Time	00:00:00.00
	Elapsed Time	00:00:00.01

Case Processing Summary

		N	Percent
Cases available in analysis	Event[a]	67	73.6%
	Censored	11	12.1%
	Total	78	85.7%
Cases dropped	Cases with missing values	13	14.3%
	Cases with negative time	0	0.0%
	Censored cases before the earliest event in a stratum	0	0.0%
	Total	13	14.3%
Total		91	100.0%

a. Dependent Variable: Survival_month

Block 0: Beginning Block

Omnibus Tests of Model Coefficients

-2 Log Likelihood
495.555

Block 1: Method = Enter

Omnibus Tests of Model Coefficients[a]

-2 Log Likelihood	Overall (score)			Change From Previous Step			Change From Previous Block		
	Chi-square	df	Sig.	Chi-square	df	Sig.	Chi-square	df	Sig.
472.987	24.667	7	.001	22.568	7	.002	22.568	7	.002

a. Beginning Block Number 1. Method = Enter

Variables in the Equation

	B	SE	Wald	df	Sig.	Exp(B)	95.0% CI for Exp(B)	
							Lower	Upper
Vascular_invasion	.680	.279	5.955	1	.015	1.974	1.143	3.408
Portal_lymphnode	.205	.271	.574	1	.449	1.228	.722	2.088
tumor_differentiation	-.526	.263	3.983	1	.046	.591	.353	.991
CA24_2	.002	.001	4.503	1	.034	1.002	1.000	1.004
CEA	.001	.003	.100	1	.752	1.001	.995	1.007
CA12_5	.001	.002	.447	1	.504	1.001	.998	1.004
plt	-.003	.002	3.550	1	.060	.997	.993	1.000

Covariate Means

	Mean
Vascular_invasion	.295
Portal_lymphnode	.321
tumor_differentiation	1.910
CA24_2	85.160
CEA	9.981
CA12_5	38.585
plt	237.333

```
COXREG Survival_month
/STATUS=Death(1)
/METHOD=FSTEP(LR) Vascular_invasion Portal_lymphnode tumor_differentiation CA24
_2 CEA CA12_5 plt
/PRINT=CI(95) SUMMARY
/CRITERIA=PIN(.05) POUT(.10) ITERATE(20).
```

Cox Regression

Notes

Output Created		25-JAN-2024 08:59:13
Comments		
Input	Data	D:\D\project\IL6 and BTC\manuscript\frontiers in immunology\Expression and prognosis.sav
	Active Dataset	1
	Filter	<none>
	Weight	<none>
	Split File	<none>
	N of Rows in Working Data File	91
Missing Value Handling	Definition of Missing	User-defined missing values are treated as missing.
Syntax		COXREG Survival_month /STATUS=Death(1) /METHOD=FSTEP(LR) Vascular_invasion Portal_lymphnode tumor_differentiation CA24_2 CEA CA12_5 plt /PRINT=CI(95) SUMMARY /CRITERIA=PIN(.05) POUT(.10) ITERATE(20).
Resources	Processor Time	00:00:00.02
	Elapsed Time	00:00:00.01

Case Processing Summary

		N	Percent
Cases available in analysis	Event[a]	67	73.6%
	Censored	11	12.1%
	Total	78	85.7%
Cases dropped	Cases with missing values	13	14.3%
	Cases with negative time	0	0.0%
	Censored cases before the earliest event in a stratum	0	0.0%
	Total	13	14.3%
Total		91	100.0%

a. Dependent Variable: Survival_month

Block 0: Beginning Block

Omnibus Tests of Model Coefficients

-2 Log Likelihood
495.555

Block 1: Method = Forward Stepwise (Likelihood Ratio)

Omnibus Tests of Model Coefficients[a]

Step	-2 Log Likelihood	Overall (score)			Change From Previous Block		
		Chi-square	df	Sig.	Chi-square	df	Sig.
4	474.106	22.471	4	.000	21.449	4	.000

a. Beginning Block Number 1. Method = Forward Stepwise (Likelihood Ratio)

Variables in the Equation

		B	SE	Wald	df	Sig.	Exp(B)	95.0% CI for Exp(B)	
								Lower	Upper
Step 4	Vascular_invasion	.725	.270	7.223	1	.007	2.064	1.217	3.502
	tumor_differentiation	-.586	.255	5.274	1	.022	.556	.337	.918
	CA24_2	.002	.001	6.334	1	.012	1.002	1.001	1.004
	plt	-.004	.002	4.520	1	.034	.996	.993	1.000

Variables not in the Equation[a]

		Score	df	Sig.
Step 4	Portal_lymphnode	.586	1	.444
	CEA	.078	1	.779
	CA12_5	.578	1	.447

a. Residual Chi Square = 1.205 with 3 df Sig. = .752

Covariate Means

	Mean
Vascular_invasion	.295
Portal_lymphnode	.321
tumor_differentiation	1.910
CA24_2	85.160
CEA	9.981
CA12_5	38.585
plt	237.333

```
COXREG DFS_month
```

```
/STATUS=Recurrence(1)
/METHOD=FSTEP(LR) sex
/PRINT=CI(95) SUMMARY
/CRITERIA=PIN(.05) POUT(.10) ITERATE(20).
```

Cox Regression

Notes

Output Created		25-JAN-2024 08:59:41
Comments		
Input	Data	D:\D\project\IL6 and BTC\manuscript\frontiers in immunology\Expression and prognosis.sav
	Active Dataset	1
	Filter	<none>
	Weight	<none>
	Split File	<none>
	N of Rows in Working Data File	91
Missing Value Handling	Definition of Missing	User-defined missing values are treated as missing.
Syntax		COXREG DFS_month /STATUS=Recurrence(1) /METHOD=FSTEP(LR) sex /PRINT=CI(95) SUMMARY /CRITERIA=PIN(.05) POUT(.10) ITERATE(20).
Resources	Processor Time	00:00:00.00
	Elapsed Time	00:00:00.00

Case Processing Summary

		N	Percent
Cases available in analysis	Event[a]	65	71.4%
	Censored	15	16.5%
	Total	80	87.9%
Cases dropped	Cases with missing values	11	12.1%
	Cases with negative time	0	0.0%
	Censored cases before the earliest event in a stratum	0	0.0%
	Total	11	12.1%
Total		91	100.0%

a. Dependent Variable: DFS_month

Block 0: Beginning Block

Omnibus Tests of Model Coefficients

-2 Log Likelihood
487.446

Block 1: Method = Forward Stepwise (Likelihood Ratio)

Omnibus Tests of Model Coefficients[a]

Step	-2 Log Likelihood
0	487.446

a. Beginning Block Number 1. Method = Forward Stepwise (Likelihood Ratio)

Variables not in the Equation[a]

		Score	df	Sig.
Step 0	sex	.001	1	.977

a. Residual Chi Square = .001 with 1 df Sig. = .977

Covariate Means

	Mean
sex	.463

```
COXREG DFS_month
/STATUS=Recurrence(1)
/METHOD=ENTER sex
/PRINT=CI(95)SUMMARY
/CRITERIA=PIN(.05)POUT(.10)ITERATE(20).
```

Cox Regression

Notes

Output Created		25-JAN-2024 08:59:49
Comments		
Input	Data	D:\D\project\IL6 and BTC\manuscript\frontiers in immunology\Expression and prognosis.sav
	Active Dataset	1
	Filter	<none>
	Weight	<none>
	Split File	<none>
	N of Rows in Working Data File	91
Missing Value Handling	Definition of Missing	User-defined missing values are treated as missing.
Syntax		COXREG DFS_month /STATUS=Recurrence(1) /METHOD=ENTER sex /PRINT=CI(95) SUMMARY /CRITERIA=PIN(.05) POUT(.10) ITERATE(20).
Resources	Processor Time	00:00:00.00
	Elapsed Time	00:00:00.01

Case Processing Summary

		N	Percent
Cases available in analysis	Event[a]	65	71.4%
	Censored	15	16.5%
	Total	80	87.9%
Cases dropped	Cases with missing values	11	12.1%
	Cases with negative time	0	0.0%
	Censored cases before the earliest event in a stratum	0	0.0%
	Total	11	12.1%
Total		91	100.0%

a. Dependent Variable: DFS_month

Block 0: Beginning Block

Omnibus Tests of Model Coefficients

-2 Log Likelihood
487.446

Block 1: Method = Enter

Omnibus Tests of Model Coefficients[a]

-2 Log Likelihood	Overall (score)			Change From Previous Step			Change From Previous Block		
	Chi-square	df	Sig.	Chi-square	df	Sig.	Chi-square	df	Sig.
487.445	.001	1	.977	.001	1	.977	.001	1	.977

a. Beginning Block Number 1. Method = Enter

Variables in the Equation

	B	SE	Wald	df	Sig.	Exp(B)	95.0% CI for Exp(B)	
							Lower	Upper
sex	.007	.250	.001	1	.977	1.007	.617	1.645

Covariate Means

	Mean
sex	.463

```
COXREG DFS_month
/STATUS=Recurrence(1)
/METHOD=ENTER age
/PRINT=CI(95)SUMMARY
/CRITERIA=PIN(.05)POUT(.10)ITERATE(20).
```

Cox Regression

Notes

Output Created		25-JAN-2024 08:59:56
Comments		
Input	Data	D:\D\project\IL6 and BTC\manuscript\frontiers in immunology\Expression and prognosis.sav
	Active Dataset	1
	Filter	<none>
	Weight	<none>
	Split File	<none>
	N of Rows in Working Data File	91
Missing Value Handling	Definition of Missing	User-defined missing values are treated as missing.
Syntax		COXREG DFS_month /STATUS=Recurrence(1) /METHOD=ENTER age /PRINT=CI(95) SUMMARY /CRITERIA=PIN(.05) POUT(.10) ITERATE(20).
Resources	Processor Time	00:00:00.00
	Elapsed Time	00:00:00.01

Case Processing Summary

		N	Percent
Cases available in analysis	Event[a]	65	71.4%
	Censored	15	16.5%
	Total	80	87.9%
Cases dropped	Cases with missing values	11	12.1%
	Cases with negative time	0	0.0%
	Censored cases before the earliest event in a stratum	0	0.0%
	Total	11	12.1%
Total		91	100.0%

a. Dependent Variable: DFS_month

Block 0: Beginning Block

Omnibus Tests of Model Coefficients

-2 Log Likelihood
487.446

Block 1: Method = Enter

Omnibus Tests of Model Coefficients[a]

-2 Log Likelihood	Overall (score)			Change From Previous Step			Change From Previous Block		
	Chi-square	df	Sig.	Chi-square	df	Sig.	Chi-square	df	Sig.
487.341	.106	1	.745	.105	1	.745	.105	1	.745

a. Beginning Block Number 1. Method = Enter

Variables in the Equation

	B	SE	Wald	df	Sig.	Exp(B)	95.0% CI for Exp(B)	
							Lower	Upper
age	-.004	.012	.106	1	.745	.996	.973	1.020

Covariate Means

	Mean
age	56.900

```
COXREG DFS_month
/STATUS=Recurrence(1)
/METHOD=ENTER wbc
/PRINT=CI(95)SUMMARY
/CRITERIA=PIN(.05)POUT(.10)ITERATE(20).
```

Cox Regression

Notes

Output Created		25-JAN-2024 09:00:03
Comments		
Input	Data	D:\D\project\IL6 and BTC\manuscript\frontiers in immunology\Expression and prognosis.sav
	Active Dataset	1
	Filter	<none>
	Weight	<none>
	Split File	<none>
	N of Rows in Working Data File	91
Missing Value Handling	Definition of Missing	User-defined missing values are treated as missing.
Syntax		COXREG DFS_month /STATUS=Recurrence(1) /METHOD=ENTER wbc /PRINT=CI(95) SUMMARY /CRITERIA=PIN(.05) POUT(.10) ITERATE(20).
Resources	Processor Time	00:00:00.02
	Elapsed Time	00:00:00.01

Case Processing Summary

		N	Percent
Cases available in analysis	Event[a]	65	71.4%
	Censored	15	16.5%
	Total	80	87.9%
Cases dropped	Cases with missing values	11	12.1%
	Cases with negative time	0	0.0%
	Censored cases before the earliest event in a stratum	0	0.0%
	Total	11	12.1%
Total		91	100.0%

a. Dependent Variable: DFS_month

Block 0: Beginning Block

Omnibus Tests of Model Coefficients

-2 Log Likelihood
487.446

Block 1: Method = Enter

Omnibus Tests of Model Coefficients[a]

-2 Log Likelihood	Overall (score)			Change From Previous Step			Change From Previous Block		
	Chi-square	df	Sig.	Chi-square	df	Sig.	Chi-square	df	Sig.
484.004	4.016	1	.045	3.442	1	.064	3.442	1	.064

a. Beginning Block Number 1. Method = Enter

Variables in the Equation

	B	SE	Wald	df	Sig.	Exp(B)	95.0% CI for Exp(B)	
							Lower	Upper
wbc	.078	.039	3.986	1	.046	1.081	1.001	1.167

Covariate Means

	Mean
wbc	7.283

```
COXREG DFS_month
/STATUS=Recurrence(1)
/METHOD=ENTER Neutrophils
/PRINT=CI(95)SUMMARY
/CRITERIA=PIN(.05)POUT(.10)ITERATE(20).
```

Cox Regression

Notes

Output Created		25-JAN-2024 09:00:12
Comments		
Input	Data	D:\D\project\IL6 and BTC\manuscript\frontiers in immunology\Expression and prognosis.sav
	Active Dataset	1
	Filter	<none>
	Weight	<none>
	Split File	<none>
	N of Rows in Working Data File	91
Missing Value Handling	Definition of Missing	User-defined missing values are treated as missing.
Syntax		COXREG DFS_month /STATUS=Recurrence(1) /METHOD=ENTER Neutrophils /PRINT=CI(95) SUMMARY /CRITERIA=PIN(.05) POUT(.10) ITERATE(20).
Resources	Processor Time	00:00:00.02
	Elapsed Time	00:00:00.01

Case Processing Summary

		N	Percent
Cases available in analysis	Event[a]	65	71.4%
	Censored	15	16.5%
	Total	80	87.9%
Cases dropped	Cases with missing values	11	12.1%
	Cases with negative time	0	0.0%
	Censored cases before the earliest event in a stratum	0	0.0%
	Total	11	12.1%
Total		91	100.0%

a. Dependent Variable: DFS_month

Block 0: Beginning Block

Omnibus Tests of Model Coefficients

-2 Log Likelihood
487.446

Block 1: Method = Enter

Omnibus Tests of Model Coefficients[a]

-2 Log Likelihood	Overall (score)			Change From Previous Step			Change From Previous Block		
	Chi-square	df	Sig.	Chi-square	df	Sig.	Chi-square	df	Sig.
485.874	1.540	1	.215	1.572	1	.210	1.572	1	.210

a. Beginning Block Number 1. Method = Enter

Variables in the Equation

	B	SE	Wald	df	Sig.	Exp(B)	95.0% CI for Exp(B)	
							Lower	Upper
Neutrophils	.014	.011	1.552	1	.213	1.014	.992	1.036

Covariate Means

	Mean
Neutrophils	66.934

```
COXREG DFS_month
/STATUS=Recurrence(1)
/METHOD=ENTER lymphocyte
/PRINT=CI(95)SUMMARY
/CRITERIA=PIN(.05)POUT(.10)ITERATE(20).
```

Cox Regression

Notes

Output Created		25-JAN-2024 09:00:19
Comments		
Input	Data	D:\D\project\IL6 and BTC\manuscript\frontiers in immunology\Expression and prognosis.sav
	Active Dataset	1
	Filter	<none>
	Weight	<none>
	Split File	<none>
	N of Rows in Working Data File	91
Missing Value Handling	Definition of Missing	User-defined missing values are treated as missing.
Syntax		COXREG DFS_month /STATUS=Recurrence(1) /METHOD=ENTER lymphocyte /PRINT=CI(95) SUMMARY /CRITERIA=PIN(.05) POUT(.10) ITERATE(20).
Resources	Processor Time	00:00:00.00
	Elapsed Time	00:00:00.00

Case Processing Summary

		N	Percent
Cases available in analysis	Event[a]	65	71.4%
	Censored	15	16.5%
	Total	80	87.9%
Cases dropped	Cases with missing values	11	12.1%
	Cases with negative time	0	0.0%
	Censored cases before the earliest event in a stratum	0	0.0%
	Total	11	12.1%
Total		91	100.0%

a. Dependent Variable: DFS_month

Block 0: Beginning Block

Omnibus Tests of Model Coefficients

-2 Log Likelihood
487.446

Block 1: Method = Enter

Omnibus Tests of Model Coefficients[a]

-2 Log Likelihood	Overall (score)			Change From Previous Step			Change From Previous Block		
	Chi-square	df	Sig.	Chi-square	df	Sig.	Chi-square	df	Sig.
484.900	2.504	1	.114	2.546	1	.111	2.546	1	.111

a. Beginning Block Number 1. Method = Enter

Variables in the Equation

	B	SE	Wald	df	Sig.	Exp(B)	95.0% CI for Exp(B)	
							Lower	Upper
lymphocyte	-.023	.014	2.501	1	.114	.977	.950	1.005

Covariate Means

	Mean
lymphocyte	21.638

```
COXREG DFS_month
/STATUS=Recurrence(1)
/METHOD=ENTER hemoglobin
/PRINT=CI(95)SUMMARY
/CRITERIA=PIN(.05)POUT(.10)ITERATE(20).
```

Cox Regression

Notes

Output Created		25-JAN-2024 09:00:26
Comments		
Input	Data	D:\D\project\IL6 and BTC\manuscript\frontiers in immunology\Expression and prognosis.sav
	Active Dataset	1
	Filter	<none>
	Weight	<none>
	Split File	<none>
	N of Rows in Working Data File	91
Missing Value Handling	Definition of Missing	User-defined missing values are treated as missing.
Syntax		COXREG DFS_month /STATUS=Recurrence(1) /METHOD=ENTER hemoglobin /PRINT=CI(95) SUMMARY /CRITERIA=PIN(.05) POUT(.10) ITERATE(20).
Resources	Processor Time	00:00:00.02
	Elapsed Time	00:00:00.01

Case Processing Summary

		N	Percent
Cases available in analysis	Event[a]	65	71.4%
	Censored	15	16.5%
	Total	80	87.9%
Cases dropped	Cases with missing values	11	12.1%
	Cases with negative time	0	0.0%
	Censored cases before the earliest event in a stratum	0	0.0%
	Total	11	12.1%
Total		91	100.0%

a. Dependent Variable: DFS_month

Block 0: Beginning Block

Omnibus Tests of Model Coefficients

-2 Log Likelihood
487.446

Block 1: Method = Enter

Omnibus Tests of Model Coefficients[a]

-2 Log Likelihood	Overall (score)			Change From Previous Step			Change From Previous Block		
	Chi-square	df	Sig.	Chi-square	df	Sig.	Chi-square	df	Sig.
487.442	.004	1	.951	.004	1	.951	.004	1	.951

a. Beginning Block Number 1. Method = Enter

Variables in the Equation

	B	SE	Wald	df	Sig.	Exp(B)	95.0% CI for Exp(B)	
							Lower	Upper
hemoglobin	.000	.007	.004	1	.951	1.000	.987	1.013

Covariate Means

	Mean
hemoglobin	119.850

```
COXREG DFS_month
/STATUS=Recurrence(1)
/METHOD=ENTER plt
/PRINT=CI(95)SUMMARY
/CRITERIA=PIN(.05)POUT(.10)ITERATE(20).
```

Cox Regression

Notes

Output Created		25-JAN-2024 09:00:33
Comments		
Input	Data	D:\D\project\IL6 and BTC\manuscript\frontiers in immunology\Expression and prognosis.sav
	Active Dataset	1
	Filter	<none>
	Weight	<none>
	Split File	<none>
	N of Rows in Working Data File	91
Missing Value Handling	Definition of Missing	User-defined missing values are treated as missing.
Syntax		COXREG DFS_month /STATUS=Recurrence(1) /METHOD=ENTER plt /PRINT=CI(95) SUMMARY /CRITERIA=PIN(.05) POUT(.10) ITERATE(20).
Resources	Processor Time	00:00:00.02
	Elapsed Time	00:00:00.00

Case Processing Summary

		N	Percent
Cases available in analysis	Event[a]	65	71.4%
	Censored	15	16.5%
	Total	80	87.9%
Cases dropped	Cases with missing values	11	12.1%
	Cases with negative time	0	0.0%
	Censored cases before the earliest event in a stratum	0	0.0%
	Total	11	12.1%
Total		91	100.0%

a. Dependent Variable: DFS_month

Block 0: Beginning Block

Omnibus Tests of Model Coefficients

-2 Log Likelihood
487.446

Block 1: Method = Enter

Omnibus Tests of Model Coefficients[a]

-2 Log Likelihood	Overall (score)			Change From Previous Step			Change From Previous Block		
	Chi-square	df	Sig.	Chi-square	df	Sig.	Chi-square	df	Sig.
483.404	3.868	1	.049	4.042	1	.044	4.042	1	.044

a. Beginning Block Number 1. Method = Enter

Variables in the Equation

	B	SE	Wald	df	Sig.	Exp(B)	95.0% CI for Exp(B)	
							Lower	Upper
plt	-.003	.002	3.860	1	.049	.997	.993	1.000

Covariate Means

	Mean
plt	239.125

```
COXREG DFS_month
/STATUS=Recurrence(1)
/METHOD=ENTER CA12_5
/PRINT=CI(95)SUMMARY
/CRITERIA=PIN(.05)POUT(.10)ITERATE(20).
```

Cox Regression

Notes

Output Created		25-JAN-2024 09:00:42
Comments		
Input	Data	D:\D\project\IL6 and BTC\manuscript\frontiers in immunology\Expression and prognosis.sav
	Active Dataset	1
	Filter	<none>
	Weight	<none>
	Split File	<none>
	N of Rows in Working Data File	91
Missing Value Handling	Definition of Missing	User-defined missing values are treated as missing.
Syntax		COXREG DFS_month /STATUS=Recurrence(1) /METHOD=ENTER CA12_5 /PRINT=CI(95) SUMMARY /CRITERIA=PIN(.05) POUT(.10) ITERATE(20).
Resources	Processor Time	00:00:00.02
	Elapsed Time	00:00:00.01

Case Processing Summary

		N	Percent
Cases available in analysis	Event[a]	63	69.2%
	Censored	15	16.5%
	Total	78	85.7%
Cases dropped	Cases with missing values	13	14.3%
	Cases with negative time	0	0.0%
	Censored cases before the earliest event in a stratum	0	0.0%
	Total	13	14.3%
Total		91	100.0%

a. Dependent Variable: DFS_month

Block 0: Beginning Block

Omnibus Tests of Model Coefficients

-2 Log Likelihood
470.161

Block 1: Method = Enter

Omnibus Tests of Model Coefficients[a]

-2 Log Likelihood	Overall (score)			Change From Previous Step			Change From Previous Block		
	Chi-square	df	Sig.	Chi-square	df	Sig.	Chi-square	df	Sig.
465.607	7.145	1	.008	4.554	1	.033	4.554	1	.033

a. Beginning Block Number 1. Method = Enter

Variables in the Equation

	B	SE	Wald	df	Sig.	Exp(B)	95.0% CI for Exp(B)	
							Lower	Upper
CA12_5	.004	.001	6.381	1	.012	1.004	1.001	1.007

Covariate Means

	Mean
CA12_5	38.506

```
COXREG DFS_month
/STATUS=Recurrence(1)
/METHOD=ENTER CA19_9
/PRINT=CI(95)SUMMARY
/CRITERIA=PIN(.05)POUT(.10)ITERATE(20).
```

Cox Regression

Notes

Output Created		25-JAN-2024 09:00:49
Comments		
Input	Data	D:\D\project\IL6 and BTC\manuscript\frontiers in immunology\Expression and prognosis.sav
	Active Dataset	1
	Filter	<none>
	Weight	<none>
	Split File	<none>
	N of Rows in Working Data File	91
Missing Value Handling	Definition of Missing	User-defined missing values are treated as missing.
Syntax		COXREG DFS_month /STATUS=Recurrence(1) /METHOD=ENTER CA19_9 /PRINT=CI(95) SUMMARY /CRITERIA=PIN(.05) POUT(.10) ITERATE(20).
Resources	Processor Time	00:00:00.00
	Elapsed Time	00:00:00.01

Case Processing Summary

		N	Percent
Cases available in analysis	Event[a]	58	63.7%
	Censored	15	16.5%
	Total	73	80.2%
Cases dropped	Cases with missing values	18	19.8%
	Cases with negative time	0	0.0%
	Censored cases before the earliest event in a stratum	0	0.0%
	Total	18	19.8%
Total		91	100.0%

a. Dependent Variable: DFS_month

Block 0: Beginning Block

Omnibus Tests of Model Coefficients

-2 Log Likelihood
426.732

Block 1: Method = Enter

Omnibus Tests of Model Coefficients[a]

-2 Log Likelihood	Overall (score)			Change From Previous Step			Change From Previous Block		
	Chi-square	df	Sig.	Chi-square	df	Sig.	Chi-square	df	Sig.
425.917	.851	1	.356	.816	1	.366	.816	1	.366

a. Beginning Block Number 1. Method = Enter

Variables in the Equation

	B	SE	Wald	df	Sig.	Exp(B)	95.0% CI for Exp(B)	
							Lower	Upper
CA19_9	.000	.001	.848	1	.357	1.000	.999	1.001

Covariate Means

	Mean
CA19_9	255.002

```
COXREG DFS_month
/STATUS = Recurrence (1)
/METHOD= ENTER CEA
/PRINT= CI (95) SUMMARY
/CRITERIA = PIN(.05) POUT (.10) ITERATE (20).
```

Cox Regression

Notes

Output Created		25-JAN-2024 09:00:54
Comments		
Input	Data	D:\D\project\IL6 and BTC\manuscript\frontiers in immunology\Expression and prognosis.sav
	Active Dataset	1
	Filter	<none>
	Weight	<none>
	Split File	<none>
	N of Rows in Working Data File	91
Missing Value Handling	Definition of Missing	User-defined missing values are treated as missing.
Syntax		COXREG DFS_month /STATUS=Recurrence(1) /METHOD=ENTER CEA /PRINT=CI(95) SUMMARY /CRITERIA=PIN(.05) POUT(.10) ITERATE(20).
Resources	Processor Time	00:00:00.00
	Elapsed Time	00:00:00.01

Case Processing Summary

		N	Percent
Cases available in analysis	Event[a]	65	71.4%
	Censored	15	16.5%
	Total	80	87.9%
Cases dropped	Cases with missing values	11	12.1%
	Cases with negative time	0	0.0%
	Censored cases before the earliest event in a stratum	0	0.0%
	Total	11	12.1%
Total		91	100.0%

a. Dependent Variable: DFS_month

Block 0: Beginning Block

Omnibus Tests of Model Coefficients

-2 Log Likelihood
487.446

Block 1: Method = Enter

Omnibus Tests of Model Coefficients[a]

-2 Log Likelihood	Overall (score)			Change From Previous Step			Change From Previous Block		
	Chi-square	df	Sig.	Chi-square	df	Sig.	Chi-square	df	Sig.
485.424	3.492	1	.062	2.022	1	.155	2.022	1	.155

a. Beginning Block Number 1. Method = Enter

Variables in the Equation

	B	SE	Wald	df	Sig.	Exp(B)	95.0% CI for Exp(B)	
							Lower	Upper
CEA	.004	.002	2.983	1	.084	1.004	.999	1.009

Covariate Means

	Mean
CEA	9.920

```
COXREG DFS_month
/STATUS=Recurrence(1)
/METHOD=ENTER CA24_2
/PRINT=CI(95)SUMMARY
/CRITERIA=PIN(.05)POUT(.10)ITERATE(20).
```

Cox Regression

Notes

Output Created		25-JAN-2024 09:01:01
Comments		
Input	Data	D:\D\project\IL6 and BTC\manuscript\frontiers in immunology\Expression and prognosis.sav
	Active Dataset	1
	Filter	<none>
	Weight	<none>
	Split File	<none>
	N of Rows in Working Data File	91
Missing Value Handling	Definition of Missing	User-defined missing values are treated as missing.
Syntax		COXREG DFS_month /STATUS=Recurrence(1) /METHOD=ENTER CA24_2 /PRINT=CI(95) SUMMARY /CRITERIA=PIN(.05) POUT(.10) ITERATE(20).
Resources	Processor Time	00:00:00.00
	Elapsed Time	00:00:00.00

Case Processing Summary

		N	Percent
Cases available in analysis	Event[a]	65	71.4%
	Censored	15	16.5%
	Total	80	87.9%
Cases dropped	Cases with missing values	11	12.1%
	Cases with negative time	0	0.0%
	Censored cases before the earliest event in a stratum	0	0.0%
	Total	11	12.1%
Total		91	100.0%

a. Dependent Variable: DFS_month

Block 0: Beginning Block

Omnibus Tests of Model Coefficients

-2 Log Likelihood
487.446

Block 1: Method = Enter

Omnibus Tests of Model Coefficients[a]

-2 Log Likelihood	Overall (score)			Change From Previous Step			Change From Previous Block		
	Chi-square	df	Sig.	Chi-square	df	Sig.	Chi-square	df	Sig.
482.342	6.242	1	.012	5.104	1	.024	5.104	1	.024

a. Beginning Block Number 1. Method = Enter

Variables in the Equation

	B	SE	Wald	df	Sig.	Exp(B)	95.0% CI for Exp(B)	
							Lower	Upper
CA24_2	.002	.001	5.975	1	.015	1.002	1.000	1.004

Covariate Means

	Mean
CA24_2	88.775

```
COXREG DFS_month
/STATUS=Recurrence(1)
/METHOD=ENTER AFP
/PRINT=CI(95)SUMMARY
/CRITERIA=PIN(.05)POUT(.10)ITERATE(20).
```

Cox Regression

Notes

Output Created		25-JAN-2024 09:01:07
Comments		
Input	Data	D:\D\project\IL6 and BTC\manuscript\frontiers in immunology\Expression and prognosis.sav
	Active Dataset	1
	Filter	<none>
	Weight	<none>
	Split File	<none>
	N of Rows in Working Data File	91
Missing Value Handling	Definition of Missing	User-defined missing values are treated as missing.
Syntax		COXREG DFS_month /STATUS=Recurrence(1) /METHOD=ENTER AFP /PRINT=CI(95) SUMMARY /CRITERIA=PIN(.05) POUT(.10) ITERATE(20).
Resources	Processor Time	00:00:00.00
	Elapsed Time	00:00:00.01

Case Processing Summary

		N	Percent
Cases available in analysis	Event[a]	65	71.4%
	Censored	15	16.5%
	Total	80	87.9%
Cases dropped	Cases with missing values	11	12.1%
	Cases with negative time	0	0.0%
	Censored cases before the earliest event in a stratum	0	0.0%
	Total	11	12.1%
Total		91	100.0%

a. Dependent Variable: DFS_month

Block 0: Beginning Block

Omnibus Tests of Model Coefficients

-2 Log Likelihood
487.446

Block 1: Method = Enter

Omnibus Tests of Model Coefficients[a]

-2 Log Likelihood	Overall (score)			Change From Previous Step			Change From Previous Block		
	Chi-square	df	Sig.	Chi-square	df	Sig.	Chi-square	df	Sig.
487.431	.015	1	.902	.015	1	.902	.015	1	.902

a. Beginning Block Number 1. Method = Enter

Variables in the Equation

	B	SE	Wald	df	Sig.	Exp(B)	95.0% CI for Exp(B)	
							Lower	Upper
AFP	.003	.022	.015	1	.902	1.003	.961	1.046

Covariate Means

	Mean
AFP	6.227

```
COXREG DFS_month
/STATUS=Recurrence(1)
/METHOD=ENTER HBsAg
/PRINT=CI(95)SUMMARY
/CRITERIA=PIN(.05)POUT(.10)ITERATE(20).
```

Cox Regression

Notes

Output Created		25-JAN-2024 09:01:14
Comments		
Input	Data	D:\D\project\IL6 and BTC\manuscript\frontiers in immunology\Expression and prognosis.sav
	Active Dataset	1
	Filter	<none>
	Weight	<none>
	Split File	<none>
	N of Rows in Working Data File	91
Missing Value Handling	Definition of Missing	User-defined missing values are treated as missing.
Syntax		COXREG DFS_month /STATUS=Recurrence(1) /METHOD=ENTER HBsAg /PRINT=CI(95) SUMMARY /CRITERIA=PIN(.05) POUT(.10) ITERATE(20).
Resources	Processor Time	00:00:00.00
	Elapsed Time	00:00:00.01

Case Processing Summary

		N	Percent
Cases available in analysis	Event[a]	65	71.4%
	Censored	15	16.5%
	Total	80	87.9%
Cases dropped	Cases with missing values	11	12.1%
	Cases with negative time	0	0.0%
	Censored cases before the earliest event in a stratum	0	0.0%
	Total	11	12.1%
Total		91	100.0%

a. Dependent Variable: DFS_month

Block 0: Beginning Block

Omnibus Tests of Model Coefficients

-2 Log Likelihood
487.446

Block 1: Method = Enter

Omnibus Tests of Model Coefficients[a]

-2 Log Likelihood	Overall (score)			Change From Previous Step			Change From Previous Block		
	Chi-square	df	Sig.	Chi-square	df	Sig.	Chi-square	df	Sig.
487.044	.437	1	.509	.402	1	.526	.402	1	.526

a. Beginning Block Number 1. Method = Enter

Variables in the Equation

	B	SE	Wald	df	Sig.	Exp(B)	95.0% CI for Exp(B)	
							Lower	Upper
HBsAg	.283	.429	.434	1	.510	1.327	.572	3.076

Covariate Means

	Mean
HBsAg	.088

```
COXREG DFS_month
/STATUS=Recurrence(1)
/METHOD=ENTER Bismuth
/PRINT=CI(95)SUMMARY
/CRITERIA=PIN(.05)POUT(.10)ITERATE(20).
```

Cox Regression

Notes

Output Created		25-JAN-2024 09:01:22
Comments		
Input	Data	D:\D\project\IL6 and BTC\manuscript\frontiers in immunology\Expression and prognosis.sav
	Active Dataset	1
	Filter	<none>
	Weight	<none>
	Split File	<none>
	N of Rows in Working Data File	91
Missing Value Handling	Definition of Missing	User-defined missing values are treated as missing.
Syntax		COXREG DFS_month /STATUS=Recurrence(1) /METHOD=ENTER Bismuth /PRINT=CI(95) SUMMARY /CRITERIA=PIN(.05) POUT(.10) ITERATE(20).
Resources	Processor Time	00:00:00.00
	Elapsed Time	00:00:00.01

Case Processing Summary

		N	Percent
Cases available in analysis	Event[a]	65	71.4%
	Censored	15	16.5%
	Total	80	87.9%
Cases dropped	Cases with missing values	11	12.1%
	Cases with negative time	0	0.0%
	Censored cases before the earliest event in a stratum	0	0.0%
	Total	11	12.1%
Total		91	100.0%

a. Dependent Variable: DFS_month

Block 0: Beginning Block

Omnibus Tests of Model Coefficients

-2 Log Likelihood
487.446

Block 1: Method = Enter

Omnibus Tests of Model Coefficients[a]

-2 Log Likelihood	Overall (score)			Change From Previous Step			Change From Previous Block		
	Chi-square	df	Sig.	Chi-square	df	Sig.	Chi-square	df	Sig.
487.228	.215	1	.643	.218	1	.641	.218	1	.641

a. Beginning Block Number 1. Method = Enter

Variables in the Equation

	B	SE	Wald	df	Sig.	Exp(B)	95.0% CI for Exp(B)	
							Lower	Upper
Bismuth	.083	.179	.215	1	.643	1.087	.765	1.543

Covariate Means

	Mean
Bismuth	2.713

```
COXREG DFS_month
/STATUS=Recurrence(1)
/METHOD=ENTER tumor_differentiation
/PRINT=CI(95)SUMMARY
/CRITERIA=PIN(.05)POUT(.10)ITERATE(20).
```

Cox Regression

Notes

Output Created		25-JAN-2024 09:01:31
Comments		
Input	Data	D:\D\project\IL6 and BTC\manuscript\frontiers in immunology\Expression and prognosis.sav
	Active Dataset	1
	Filter	<none>
	Weight	<none>
	Split File	<none>
	N of Rows in Working Data File	91
Missing Value Handling	Definition of Missing	User-defined missing values are treated as missing.
Syntax		COXREG DFS_month /STATUS=Recurrence(1) /METHOD=ENTER tumor_differentiation /PRINT=CI(95) SUMMARY /CRITERIA=PIN(.05) POUT(.10) ITERATE(20).
Resources	Processor Time	00:00:00.02
	Elapsed Time	00:00:00.01

Case Processing Summary

		N	Percent
Cases available in analysis	Event[a]	64	70.3%
	Censored	15	16.5%
	Total	79	86.8%
Cases dropped	Cases with missing values	12	13.2%
	Cases with negative time	0	0.0%
	Censored cases before the earliest event in a stratum	0	0.0%
	Total	12	13.2%
Total		91	100.0%

a. Dependent Variable: DFS_month

Block 0: Beginning Block

Omnibus Tests of Model Coefficients

-2 Log Likelihood
478.728

Block 1: Method = Enter

Omnibus Tests of Model Coefficients[a]

-2 Log Likelihood	Overall (score)			Change From Previous Step			Change From Previous Block		
	Chi-square	df	Sig.	Chi-square	df	Sig.	Chi-square	df	Sig.
473.462	5.321	1	.021	5.267	1	.022	5.267	1	.022

a. Beginning Block Number 1. Method = Enter

Variables in the Equation

	B	SE	Wald	df	Sig.	Exp(B)	95.0% CI for Exp(B)	
							Lower	Upper
tumor_differentiation	-.579	.250	5.373	1	.020	.560	.343	.914

Covariate Means

	Mean
tumor_differentiation	1.886

```
COXREG DFS_month
/STATUS=Recurrence(1)
/METHOD=ENTER Portal_lymphnode
/PRINT=CI(95)SUMMARY
/CRITERIA=PIN(.05)POUT(.10)ITERATE(20).
```

Cox Regression

Notes

Output Created		25-JAN-2024 09:01:39
Comments		
Input	Data	D:\D\project\IL6 and BTC\manuscript\frontiers in immunology\Expression and prognosis.sav
	Active Dataset	1
	Filter	<none>
	Weight	<none>
	Split File	<none>
	N of Rows in Working Data File	91
Missing Value Handling	Definition of Missing	User-defined missing values are treated as missing.
Syntax		COXREG DFS_month /STATUS=Recurrence(1) /METHOD=ENTER Portal_lymphnode /PRINT=CI(95) SUMMARY /CRITERIA=PIN(.05) POUT(.10) ITERATE(20).
Resources	Processor Time	00:00:00.02
	Elapsed Time	00:00:00.01

Case Processing Summary

		N	Percent
Cases available in analysis	Event[a]	65	71.4%
	Censored	15	16.5%
	Total	80	87.9%
Cases dropped	Cases with missing values	11	12.1%
	Cases with negative time	0	0.0%
	Censored cases before the earliest event in a stratum	0	0.0%
	Total	11	12.1%
Total		91	100.0%

a. Dependent Variable: DFS_month

Block 0: Beginning Block

Omnibus Tests of Model Coefficients

-2 Log Likelihood
487.446

Block 1: Method = Enter

Omnibus Tests of Model Coefficients[a]

-2 Log Likelihood	Overall (score)			Change From Previous Step			Change From Previous Block		
	Chi-square	df	Sig.	Chi-square	df	Sig.	Chi-square	df	Sig.
484.564	3.116	1	.078	2.882	1	.090	2.882	1	.090

a. Beginning Block Number 1. Method = Enter

Variables in the Equation

	B	SE	Wald	df	Sig.	Exp(B)	95.0% CI for Exp(B)	
							Lower	Upper
Portal_lymphnode	.467	.267	3.061	1	.080	1.595	.945	2.689

Covariate Means

	Mean
Portal_lymphnode	.325

```
COXREG DFS_month
/STATUS=Recurrence(1)
/METHOD=ENTER Distant_lymphnode_metastasis
/PRINT=CI(95)SUMMARY
/CRITERIA=PIN(.05)POUT(.10)ITERATE(20).
```

Cox Regression

Notes

Output Created		25-JAN-2024 09:01:46
Comments		
Input	Data	D:\D\project\IL6 and BTC\manuscript\frontiers in immunology\Expression and prognosis.sav
	Active Dataset	1
	Filter	<none>
	Weight	<none>
	Split File	<none>
	N of Rows in Working Data File	91
Missing Value Handling	Definition of Missing	User-defined missing values are treated as missing.
Syntax		COXREG DFS_month<br>/STATUS=Recurrence(1)<br>/METHOD=ENTER Distant_lymphnode_metastasis<br>/PRINT=CI(95) SUMMARY<br>/CRITERIA=PIN(.05) POUT(.10) ITERATE(20).
Resources	Processor Time	00:00:00.02
	Elapsed Time	00:00:00.01

Case Processing Summary

		N	Percent
Cases available in analysis	Event[a]	65	71.4%
	Censored	15	16.5%
	Total	80	87.9%
Cases dropped	Cases with missing values	11	12.1%
	Cases with negative time	0	0.0%
	Censored cases before the earliest event in a stratum	0	0.0%
	Total	11	12.1%
Total		91	100.0%

a. Dependent Variable: DFS_month

Block 0: Beginning Block

Omnibus Tests of Model Coefficients

-2 Log Likelihood
487.446

Block 1: Method = Enter

Omnibus Tests of Model Coefficients[a]

-2 Log Likelihood	Overall (score)			Change From Previous Step			Change From Previous Block		
	Chi-square	df	Sig.	Chi-square	df	Sig.	Chi-square	df	Sig.
485.275	2.549	1	.110	2.172	1	.141	2.172	1	.141

a. Beginning Block Number 1. Method = Enter

Variables in the Equation

	B	SE	Wald	df	Sig.	Exp(B)	95.0% CI for Exp(B)	
							Lower	Upper
Distant_lymphnode_metastasis	.572	.363	2.482	1	.115	1.771	.870	3.607

Covariate Means

	Mean
Distant_lymphnode_metastasis	.125

```
COXREG DFS_month
/STATUS=Recurrence(1)
/METHOD=ENTER Vascular_invasion
/PRINT=CI(95)SUMMARY
/CRITERIA=PIN(.05)POUT(.10)ITERATE(20).
```

Cox Regression

Notes

Output Created		25-JAN-2024 09:01:54
Comments		
Input	Data	D:\D\project\IL6 and BTC\manuscript\frontiers in immunology\Expression and prognosis.sav
	Active Dataset	1
	Filter	<none>
	Weight	<none>
	Split File	<none>
	N of Rows in Working Data File	91
Missing Value Handling	Definition of Missing	User-defined missing values are treated as missing.
Syntax		COXREG DFS_month /STATUS=Recurrence(1) /METHOD=ENTER Vascular_invasion /PRINT=CI(95) SUMMARY /CRITERIA=PIN(.05) POUT(.10) ITERATE(20).
Resources	Processor Time	00:00:00.00
	Elapsed Time	00:00:00.00

Case Processing Summary

		N	Percent
Cases available in analysis	Event[a]	65	71.4%
	Censored	15	16.5%
	Total	80	87.9%
Cases dropped	Cases with missing values	11	12.1%
	Cases with negative time	0	0.0%
	Censored cases before the earliest event in a stratum	0	0.0%
	Total	11	12.1%
Total		91	100.0%

a. Dependent Variable: DFS_month

Block 0: Beginning Block

Omnibus Tests of Model Coefficients

-2 Log Likelihood
487.446

Block 1: Method = Enter

Omnibus Tests of Model Coefficients[a]

-2 Log Likelihood	Overall (score)			Change From Previous Step			Change From Previous Block		
	Chi-square	df	Sig.	Chi-square	df	Sig.	Chi-square	df	Sig.
479.572	8.975	1	.003	7.875	1	.005	7.875	1	.005

a. Beginning Block Number 1. Method = Enter

Variables in the Equation

	B	SE	Wald	df	Sig.	Exp(B)	95.0% CI for Exp(B)	
							Lower	Upper
Vascular_invasion	.779	.266	8.576	1	.003	2.180	1.294	3.672

Covariate Means

	Mean
Vascular_invasion	.313

```
COXREG DFS_month
/STATUS=Recurrence(1)
/METHOD=ENTER Perineural_invasion
/PRINT=CI(95)SUMMARY
/CRITERIA=PIN(.05)POUT(.10)ITERATE(20).
```

Cox Regression

Notes

Output Created		25-JAN-2024 09:02:01
Comments		
Input	Data	D:\D\project\IL6 and BTC\manuscript\frontiers in immunology\Expression and prognosis.sav
	Active Dataset	1
	Filter	<none>
	Weight	<none>
	Split File	<none>
	N of Rows in Working Data File	91
Missing Value Handling	Definition of Missing	User-defined missing values are treated as missing.
Syntax		COXREG DFS_month /STATUS=Recurrence(1) /METHOD=ENTER Perineural_invasion /PRINT=CI(95) SUMMARY /CRITERIA=PIN(.05) POUT(.10) ITERATE(20).
Resources	Processor Time	00:00:00.00
	Elapsed Time	00:00:00.00

Case Processing Summary

		N	Percent
Cases available in analysis	Event[a]	65	71.4%
	Censored	15	16.5%
	Total	80	87.9%
Cases dropped	Cases with missing values	11	12.1%
	Cases with negative time	0	0.0%
	Censored cases before the earliest event in a stratum	0	0.0%
	Total	11	12.1%
Total		91	100.0%

a. Dependent Variable: DFS_month

Block 0: Beginning Block

Omnibus Tests of Model Coefficients

-2 Log Likelihood
487.446

Block 1: Method = Enter

Omnibus Tests of Model Coefficients[a]

-2 Log Likelihood	Overall (score)			Change From Previous Step			Change From Previous Block		
	Chi-square	df	Sig.	Chi-square	df	Sig.	Chi-square	df	Sig.
486.936	.472	1	.492	.510	1	.475	.510	1	.475

a. Beginning Block Number 1. Method = Enter

Variables in the Equation

	B	SE	Wald	df	Sig.	Exp(B)	95.0% CI for Exp(B)	
							Lower	Upper
Perineural_invasion	-.123	.179	.469	1	.493	.884	.622	1.257

Covariate Means

	Mean
Perineural_invasion	.325

```
 COXREG DFS_month
 /STATUS=Recurrence(1)
 /METHOD=ENTER Vascular_invasion Portal_lymphnode tumor_differentiation CA24_2
CEA CA12_5 plt wbc
  age sex
 /PRINT=CI(95)SUMMARY
 /CRITERIA=PIN(.05)POUT(.10)ITERATE(20).
```

Cox Regression

Notes

Output Created		25-JAN-2024 09:03:07
Comments		
Input	Data	D:\D\project\IL6 and BTC\manuscript\frontiers in immunology\Expression and prognosis.sav
	Active Dataset	1
	Filter	<none>
	Weight	<none>
	Split File	<none>
	N of Rows in Working Data File	91
Missing Value Handling	Definition of Missing	User-defined missing values are treated as missing.
Syntax		COXREG DFS_month /STATUS=Recurrence(1) /METHOD=ENTER Vascular_invasion Portal_lymphnode tumor_differentiation CA24_2 CEA CA12_5 plt wbc age sex /PRINT=CI(95) SUMMARY /CRITERIA=PIN(.05) POUT(.10) ITERATE(20).
Resources	Processor Time	00:00:00.00
	Elapsed Time	00:00:00.01

Case Processing Summary

		N	Percent
Cases available in analysis	Event[a]	62	68.1%
	Censored	15	16.5%
	Total	77	84.6%
Cases dropped	Cases with missing values	14	15.4%
	Cases with negative time	0	0.0%
	Censored cases before the earliest event in a stratum	0	0.0%
	Total	14	15.4%
Total		91	100.0%

a. Dependent Variable: DFS_month

Block 0: Beginning Block

Omnibus Tests of Model Coefficients

-2 Log Likelihood
461.496

Block 1: Method = Enter

Omnibus Tests of Model Coefficients[a]

-2 Log Likelihood	Overall (score)			Change From Previous Step			Change From Previous Block		
	Chi-square	df	Sig.	Chi-square	df	Sig.	Chi-square	df	Sig.
428.873	35.458	10	.000	32.622	10	.000	32.622	10	.000

a. Beginning Block Number 1. Method = Enter

Variables in the Equation

	B	SE	Wald	df	Sig.	Exp(B)	95.0% CI for Exp(B)	
							Lower	Upper
Vascular_invasion	.676	.304	4.965	1	.026	1.967	1.085	3.565
Portal_lymphnode	.253	.303	.697	1	.404	1.287	.711	2.330
tumor_differentiation	-.805	.354	5.172	1	.023	.447	.223	.895
CA24_2	.002	.001	4.959	1	.026	1.002	1.000	1.004
CEA	-.001	.003	.048	1	.827	.999	.993	1.005
CA12_5	.002	.002	1.122	1	.289	1.002	.998	1.005
plt	-.004	.002	5.114	1	.024	.996	.992	.999
wbc	.141	.059	5.693	1	.017	1.152	1.026	1.294
age	-.017	.014	1.504	1	.220	.983	.956	1.010
sex	-.175	.314	.312	1	.576	.839	.454	1.552

Covariate Means

	Mean
Vascular_invasion	.299
Portal_lymphnode	.325
tumor_differentiation	1.909
CA24_2	85.552
CEA	10.051
CA12_5	38.935
plt	237.247
wbc	7.112
age	57.013
sex	.468

```
COXREG DFS_month
/STATUS = Recurrence (1 )
```

```
 /METHOD=FSTEP(LR) Vascular_invasion Portal_lymphnode tumor_differentiation CA24
_2 CEA CA12_5 plt
   wbc age sex
 /PRINT=CI(95) SUMMARY
 /CRITERIA=PIN(.05) POUT(.10) ITERATE(20).
```

Cox Regression

Notes

Output Created		25-JAN-2024 09:03:15
Comments		
Input	Data	D:\D\project\IL6 and BTC\manuscript\frontiers in immunology\Expression and prognosis.sav
	Active Dataset	1
	Filter	<none>
	Weight	<none>
	Split File	<none>
	N of Rows in Working Data File	91
Missing Value Handling	Definition of Missing	User-defined missing values are treated as missing.
Syntax		COXREG DFS_month /STATUS=Recurrence(1) /METHOD=FSTEP(LR) Vascular_invasion Portal_lymphnode tumor_differentiation CA24_2 CEA CA12_5 plt wbc age sex /PRINT=CI(95) SUMMARY /CRITERIA=PIN(.05) POUT(.10) ITERATE(20).
Resources	Processor Time	00:00:00.02
	Elapsed Time	00:00:00.01

Case Processing Summary

		N	Percent
Cases available in analysis	Event[a]	62	68.1%
	Censored	15	16.5%
	Total	77	84.6%
Cases dropped	Cases with missing values	14	15.4%
	Cases with negative time	0	0.0%
	Censored cases before the earliest event in a stratum	0	0.0%
	Total	14	15.4%
Total		91	100.0%

a. Dependent Variable: DFS_month

Block 0: Beginning Block

Omnibus Tests of Model Coefficients

-2 Log Likelihood
461.496

Block 1: Method = Forward Stepwise (Likelihood Ratio)

Omnibus Tests of Model Coefficients[a]

Step	-2 Log Likelihood	Overall (score)			Change From Previous Block		
		Chi-square	df	Sig.	Chi-square	df	Sig.
4	439.544	25.851	4	.000	21.951	4	.000

a. Beginning Block Number 1. Method = Forward Stepwise (Likelihood Ratio)

Variables in the Equation

		B	SE	Wald	df	Sig.	Exp(B)	95.0% CI for Exp(B)	
								Lower	Upper
Step 4	Vascular_invasion	.776	.281	7.614	1	.006	2.172	1.252	3.768
	CA24_2	.002	.001	3.739	1	.053	1.002	1.000	1.004
	CA12_5	.003	.002	3.503	1	.061	1.003	1.000	1.006
	wbc	.175	.057	9.465	1	.002	1.191	1.066	1.331

Variables not in the Equation[a]

		Score	df	Sig.
Step 4	Portal_lymphnode	1.036	1	.309
	tumor_differentiation	2.725	1	.099
	CEA	.150	1	.698
	plt	3.796	1	.051
	age	.020	1	.888
	sex	.039	1	.843

a. Residual Chi Square = 10.300 with 6 df Sig. = .113

Covariate Means

	Mean
Vascular_invasion	.299
Portal_lymphnode	.325
tumor_differentiation	1.909
CA24_2	85.552
CEA	10.051
CA12_5	38.935
plt	237.247
wbc	7.112
age	57.013
sex	.468

```
CROSSTABS
/TABLES= tumor_differentiation BY IL6R CRP STAT3 CK19 GP130 JAK2 IL6
/FORMAT= AVALUE TABLES
/STATISTICS = CHISQ
/CELLS= COUNT COLUMN
/COUNT ROUND CELL.
```

Crosstabs

Notes

Output Created		25-JAN-2024 09:03:57
Comments		
Input	Data	D:\D\project\IL6 and BTC\manuscript\frontiers in immunology\Expression and prognosis.sav
	Active Dataset	1
	Filter	<none>
	Weight	<none>
	Split File	<none>
	N of Rows in Working Data File	91
Missing Value Handling	Definition of Missing	User-defined missing values are treated as missing.
	Cases Used	Statistics for each table are based on all the cases with valid data in the specified range(s) for all variables in each table.
Syntax		CROSSTABS<br>/TABLES=tumor_differentiation BY IL6R CRP STAT3 CK19 GP130 JAK2 IL6<br>/FORMAT=AVALUE TABLES<br>/STATISTICS=CHISQ<br>/CELLS=COUNT COLUMN<br>/COUNT ROUND CELL.
Resources	Processor Time	00:00:00.02
	Elapsed Time	00:00:00.02
	Dimensions Requested	2
	Cells Available	524245

Case Processing Summary

	Cases					
	Valid		Missing		Total	
	N	Percent	N	Percent	N	Percent
tumor_differentiation * IL6R	90	98.9%	1	1.1%	91	100.0%
tumor_differentiation * CRP	90	98.9%	1	1.1%	91	100.0%
tumor_differentiation * STAT3	90	98.9%	1	1.1%	91	100.0%
tumor_differentiation * CK19	90	98.9%	1	1.1%	91	100.0%
tumor_differentiation * GP130	90	98.9%	1	1.1%	91	100.0%
tumor_differentiation * JAK2	90	98.9%	1	1.1%	91	100.0%
tumor_differentiation * IL6	90	98.9%	1	1.1%	91	100.0%

tumor_differentiation * IL6R

Crosstab

			IL6R		Total
			0	1	
tumor_differentiation	1	Count	9	7	16
		% within IL6R	20.0%	15.6%	17.8%
	2	Count	34	34	68
		% within IL6R	75.6%	75.6%	75.6%
	3	Count	2	4	6
		% within IL6R	4.4%	8.9%	6.7%
Total		Count	45	45	90
		% within IL6R	100.0%	100.0%	100.0%

Chi-Square Tests

	Value	df	Asymptotic Significance (2-sided)
Pearson Chi-Square	.917[a]	2	.632
Likelihood Ratio	.930	2	.628
Linear-by-Linear Association	.757	1	.384
N of Valid Cases	90		

a. 2 cells (33.3%) have expected count less than 5. The minimum expected count is 3.00.

tumor_differentiation * CRP

Crosstab

			CRP		Total
			0	1	
tumor_differentiation	1	Count	10	6	16
		% within CRP	18.2%	17.1%	17.8%
	2	Count	39	29	68
		% within CRP	70.9%	82.9%	75.6%
	3	Count	6	0	6
		% within CRP	10.9%	0.0%	6.7%
Total		Count	55	35	90
		% within CRP	100.0%	100.0%	100.0%

Chi-Square Tests

	Value	df	Asymptotic Significance (2-sided)
Pearson Chi-Square	4.235[a]	2	.120
Likelihood Ratio	6.323	2	.042
Linear-by-Linear Association	.888	1	.346
N of Valid Cases	90		

a. 2 cells (33.3%) have expected count less than 5. The minimum expected count is 2.33.

tumor_differentiation * STAT3

Crosstab

			STAT3		Total
			0	1	
tumor_differentiation	1	Count	9	7	16
		% within STAT3	16.4%	20.0%	17.8%
	2	Count	44	24	68
		% within STAT3	80.0%	68.6%	75.6%
	3	Count	2	4	6
		% within STAT3	3.6%	11.4%	6.7%
Total		Count	55	35	90
		% within STAT3	100.0%	100.0%	100.0%

Chi-Square Tests

	Value	df	Asymptotic Significance (2-sided)
Pearson Chi-Square	2.477[a]	2	.290
Likelihood Ratio	2.419	2	.298
Linear-by-Linear Association	.157	1	.692
N of Valid Cases	90		

a. 2 cells (33.3%) have expected count less than 5. The minimum expected count is 2.33.

tumor_differentiation * CK19

Crosstab

			CK19		Total
			0	1	
tumor_differentiation	1	Count	9	7	16
		% within CK19	18.4%	17.1%	17.8%
	2	Count	36	32	68
		% within CK19	73.5%	78.0%	75.6%
	3	Count	4	2	6
		% within CK19	8.2%	4.9%	6.7%
Total		Count	49	41	90
		% within CK19	100.0%	100.0%	100.0%

Chi-Square Tests

	Value	df	Asymptotic Significance (2-sided)
Pearson Chi-Square	.444[a]	2	.801
Likelihood Ratio	.454	2	.797
Linear-by-Linear Association	.038	1	.846
N of Valid Cases	90		

a. 2 cells (33.3%) have expected count less than 5. The minimum expected count is 2.73.

tumor_differentiation * GP130

Crosstab

			GP130		Total
			0	1	
tumor_differentiation	1	Count	10	6	16
		% within GP130	18.5%	16.7%	17.8%
	2	Count	41	27	68
		% within GP130	75.9%	75.0%	75.6%
	3	Count	3	3	6
		% within GP130	5.6%	8.3%	6.7%
Total		Count	54	36	90
		% within GP130	100.0%	100.0%	100.0%

Chi-Square Tests

	Value	df	Asymptotic Significance (2-sided)
Pearson Chi-Square	.294[a]	2	.863
Likelihood Ratio	.289	2	.865
Linear-by-Linear Association	.197	1	.657
N of Valid Cases	90		

a. 2 cells (33.3%) have expected count less than 5. The minimum expected count is 2.40.

tumor_differentiation * JAK2

Crosstab

			JAK2		Total
			0	1	
tumor_differentiation	1	Count	8	8	16
		% within JAK2	14.0%	24.2%	17.8%
	2	Count	45	23	68
		% within JAK2	78.9%	69.7%	75.6%
	3	Count	4	2	6
		% within JAK2	7.0%	6.1%	6.7%
Total		Count	57	33	90
		% within JAK2	100.0%	100.0%	100.0%

Chi-Square Tests

	Value	df	Asymptotic Significance (2-sided)
Pearson Chi-Square	1.490[a]	2	.475
Likelihood Ratio	1.449	2	.485
Linear-by-Linear Association	1.110	1	.292
N of Valid Cases	90		

a. 2 cells (33.3%) have expected count less than 5. The minimum expected count is 2.20.

tumor_differentiation * IL6

Crosstab

			IL6		Total
			0	1	
tumor_differentiation	1	Count	8	8	16
		% within IL6	17.4%	18.2%	17.8%
	2	Count	37	31	68
		% within IL6	80.4%	70.5%	75.6%
	3	Count	1	5	6
		% within IL6	2.2%	11.4%	6.7%
Total		Count	46	44	90
		% within IL6	100.0%	100.0%	100.0%

Chi-Square Tests

	Value	df	Asymptotic Significance (2-sided)
Pearson Chi-Square	3.153[a]	2	.207
Likelihood Ratio	3.397	2	.183
Linear-by-Linear Association	.676	1	.411
N of Valid Cases	90		

a. 2 cells (33.3%) have expected count less than 5. The minimum expected count is 2.93.

```
CROSSTABS
/TABLES=TNM BY IL6R CRP STAT3 CK19 GP130 JAK2 IL6
/FORMAT=AVALUE TABLES
/STATISTICS=CHISQ
/CELLS=COUNT COLUMN
/COUNT ROUND CELL.
```

Crosstabs

Notes

Output Created		25-JAN-2024 09:04:21
Comments		
Input	Data	D:\D\project\IL6 and BTC\manuscript\frontiers in immunology\Expression and prognosis.sav
	Active Dataset	1
	Filter	<none>
	Weight	<none>
	Split File	<none>
	N of Rows in Working Data File	91
Missing Value Handling	Definition of Missing	User-defined missing values are treated as missing.
	Cases Used	Statistics for each table are based on all the cases with valid data in the specified range(s) for all variables in each table.
Syntax		CROSSTABS<br>/TABLES=TNM BY IL6R CRP STAT3 CK19 GP130 JAK2 IL6<br>/FORMAT=AVALUE TABLES<br>/STATISTICS=CHISQ<br>/CELLS=COUNT COLUMN<br>/COUNT ROUND CELL.
Resources	Processor Time	00:00:00.02
	Elapsed Time	00:00:00.02
	Dimensions Requested	2
	Cells Available	524245

Case Processing Summary

	Cases					
	Valid		Missing		Total	
	N	Percent	N	Percent	N	Percent
TNM * IL6R	91	100.0%	0	0.0%	91	100.0%
TNM * CRP	91	100.0%	0	0.0%	91	100.0%
TNM * STAT3	91	100.0%	0	0.0%	91	100.0%
TNM * CK19	91	100.0%	0	0.0%	91	100.0%
TNM * GP130	91	100.0%	0	0.0%	91	100.0%
TNM * JAK2	91	100.0%	0	0.0%	91	100.0%
TNM * IL6	91	100.0%	0	0.0%	91	100.0%

TNM * IL6R

Crosstab

			IL6R		Total
			0	1	
TNM	1	Count	5	6	11
		% within IL6R	11.1%	13.0%	12.1%
	2	Count	15	19	34
		% within IL6R	33.3%	41.3%	37.4%
	3	Count	17	16	33
		% within IL6R	37.8%	34.8%	36.3%
	4	Count	8	5	13
		% within IL6R	17.8%	10.9%	14.3%
Total		Count	45	46	91
		% within IL6R	100.0%	100.0%	100.0%

Chi-Square Tests

	Value	df	Asymptotic Significance (2-sided)
Pearson Chi-Square	1.273[a]	3	.735
Likelihood Ratio	1.281	3	.734
Linear-by-Linear Association	1.018	1	.313
N of Valid Cases	91		

a. 0 cells (0.0%) have expected count less than 5. The minimum expected count is 5.44.

TNM * CRP

Crosstab

			CRP		Total
			0	1	
TNM	1	Count	7	4	11
		% within CRP	12.5%	11.4%	12.1%
	2	Count	24	10	34
		% within CRP	42.9%	28.6%	37.4%
	3	Count	17	16	33
		% within CRP	30.4%	45.7%	36.3%
	4	Count	8	5	13
		% within CRP	14.3%	14.3%	14.3%
Total		Count	56	35	91
		% within CRP	100.0%	100.0%	100.0%

Chi-Square Tests

	Value	df	Asymptotic Significance (2-sided)
Pearson Chi-Square	2.598[a]	3	.458
Likelihood Ratio	2.607	3	.456
Linear-by-Linear Association	.740	1	.390
N of Valid Cases	91		

a. 1 cells (12.5%) have expected count less than 5. The minimum expected count is 4.23.

TNM * STAT3

Crosstab

			STAT3		Total
			0	1	
TNM	1	Count	6	5	11
		% within STAT3	10.7%	14.3%	12.1%
	2	Count	19	15	34
		% within STAT3	33.9%	42.9%	37.4%
	3	Count	22	11	33
		% within STAT3	39.3%	31.4%	36.3%
	4	Count	9	4	13
		% within STAT3	16.1%	11.4%	14.3%
Total		Count	56	35	91
		% within STAT3	100.0%	100.0%	100.0%

Chi-Square Tests

	Value	df	Asymptotic Significance (2-sided)
Pearson Chi-Square	1.378[a]	3	.711
Likelihood Ratio	1.384	3	.709
Linear-by-Linear Association	1.177	1	.278
N of Valid Cases	91		

a. 1 cells (12.5%) have expected count less than 5. The minimum expected count is 4.23.

TNM * CK19

Crosstab

			CK19		Total
			0	1	
TNM	1	Count	5	6	11
		% within CK19	10.2%	14.3%	12.1%
	2	Count	15	19	34
		% within CK19	30.6%	45.2%	37.4%
	3	Count	21	12	33
		% within CK19	42.9%	28.6%	36.3%
	4	Count	8	5	13
		% within CK19	16.3%	11.9%	14.3%
Total		Count	49	42	91
		% within CK19	100.0%	100.0%	100.0%

Chi-Square Tests

	Value	df	Asymptotic Significance (2-sided)
Pearson Chi-Square	3.189[a]	3	.363
Likelihood Ratio	3.208	3	.361
Linear-by-Linear Association	2.132	1	.144
N of Valid Cases	91		

a. 0 cells (0.0%) have expected count less than 5. The minimum expected count is 5.08.

TNM * GP130

Crosstab

			GP130		Total
			0	1	
TNM	1	Count	4	7	11
		% within GP130	7.3%	19.4%	12.1%
	2	Count	21	13	34
		% within GP130	38.2%	36.1%	37.4%
	3	Count	20	13	33
		% within GP130	36.4%	36.1%	36.3%
	4	Count	10	3	13
		% within GP130	18.2%	8.3%	14.3%
Total		Count	55	36	91
		% within GP130	100.0%	100.0%	100.0%

Chi-Square Tests

	Value	df	Asymptotic Significance (2-sided)
Pearson Chi-Square	4.169[a]	3	.244
Likelihood Ratio	4.205	3	.240
Linear-by-Linear Association	2.859	1	.091
N of Valid Cases	91		

a. 1 cells (12.5%) have expected count less than 5. The minimum expected count is 4.35.

TNM * JAK2

Crosstab

			JAK2		Total
			0	1	
TNM	1	Count	5	6	11
		% within JAK2	8.6%	18.2%	12.1%
	2	Count	21	13	34
		% within JAK2	36.2%	39.4%	37.4%
	3	Count	23	10	33
		% within JAK2	39.7%	30.3%	36.3%
	4	Count	9	4	13
		% within JAK2	15.5%	12.1%	14.3%
Total		Count	58	33	91
		% within JAK2	100.0%	100.0%	100.0%

Chi-Square Tests

	Value	df	Asymptotic Significance (2-sided)
Pearson Chi-Square	2.325[a]	3	.508
Likelihood Ratio	2.270	3	.518
Linear-by-Linear Association	1.770	1	.183
N of Valid Cases	91		

a. 2 cells (25.0%) have expected count less than 5. The minimum expected count is 3.99.

TNM * IL6

Crosstab

			IL6		Total
			0	1	
TNM	1	Count	6	5	11
		% within IL6	12.8%	11.4%	12.1%
	2	Count	15	19	34
		% within IL6	31.9%	43.2%	37.4%
	3	Count	20	13	33
		% within IL6	42.6%	29.5%	36.3%
	4	Count	6	7	13
		% within IL6	12.8%	15.9%	14.3%
Total		Count	47	44	91
		% within IL6	100.0%	100.0%	100.0%

Chi-Square Tests

	Value	df	Asymptotic Significance (2-sided)
Pearson Chi-Square	2.027[a]	3	.567
Likelihood Ratio	2.037	3	.565
Linear-by-Linear Association	.082	1	.775
N of Valid Cases	91		

a. 0 cells (0.0%) have expected count less than 5. The minimum expected count is 5.32.

```
CROSSTABS
/TABLES= Portal_lymphnode BY IL6R CRP STAT3 CK19 GP130 JAK2 IL6
/FORMAT= AVALUE TABLES
/STATISTICS = CHISQ
/CELLS= COUNT COLUMN
/COUNT ROUND CELL.
```

Crosstabs

Notes

Output Created		25-JAN-2024 09:04:40
Comments		
Input	Data	D:\D\project\IL6 and BTC\manuscript\frontiers in immunology\Expression and prognosis.sav
	Active Dataset	1
	Filter	<none>
	Weight	<none>
	Split File	<none>
	N of Rows in Working Data File	91
Missing Value Handling	Definition of Missing	User-defined missing values are treated as missing.
	Cases Used	Statistics for each table are based on all the cases with valid data in the specified range(s) for all variables in each table.
Syntax		CROSSTABS<br>/TABLES=Portal_lymphnode BY IL6R CRP STAT3 CK19 GP130 JAK2 IL6<br>/FORMAT=AVALUE TABLES<br>/STATISTICS=CHISQ<br>/CELLS=COUNT COLUMN<br>/COUNT ROUND CELL.
Resources	Processor Time	00:00:00.02
	Elapsed Time	00:00:00.02
	Dimensions Requested	2
	Cells Available	524245

Case Processing Summary

	Cases					
	Valid		Missing		Total	
	N	Percent	N	Percent	N	Percent
Portal_lymphnode * IL6R	91	100.0%	0	0.0%	91	100.0%
Portal_lymphnode * CRP	91	100.0%	0	0.0%	91	100.0%
Portal_lymphnode * STAT3	91	100.0%	0	0.0%	91	100.0%
Portal_lymphnode * CK19	91	100.0%	0	0.0%	91	100.0%
Portal_lymphnode * GP130	91	100.0%	0	0.0%	91	100.0%
Portal_lymphnode * JAK2	91	100.0%	0	0.0%	91	100.0%
Portal_lymphnode * IL6	91	100.0%	0	0.0%	91	100.0%

Portal_lymphnode * IL6R

Crosstab

			IL6R		Total
			0	1	
Portal_lymphnode	0	Count	28	33	61
		% within IL6R	62.2%	71.7%	67.0%
	1	Count	17	13	30
		% within IL6R	37.8%	28.3%	33.0%
Total		Count	45	46	91
		% within IL6R	100.0%	100.0%	100.0%

Chi-Square Tests

	Value	df	Asymptotic Significance (2-sided)	Exact Sig. (2-sided)	Exact Sig. (1-sided)
Pearson Chi-Square	.932[a]	1	.334		
Continuity Correction[b]	.551	1	.458		
Likelihood Ratio	.934	1	.334		
Fisher's Exact Test				.378	.229
Linear-by-Linear Association	.922	1	.337		
N of Valid Cases	91				

a. 0 cells (0.0%) have expected count less than 5. The minimum expected count is 14.84.

b. Computed only for a 2x2 table

Portal_lymphnode * CRP

Crosstab

			CRP		Total
			0	1	
Portal_lymphnode	0	Count	40	21	61
		% within CRP	71.4%	60.0%	67.0%
	1	Count	16	14	30
		% within CRP	28.6%	40.0%	33.0%
Total		Count	56	35	91
		% within CRP	100.0%	100.0%	100.0%

Chi-Square Tests

	Value	df	Asymptotic Significance (2-sided)	Exact Sig. (2-sided)	Exact Sig. (1-sided)
Pearson Chi-Square	1.273[a]	1	.259		
Continuity Correction[b]	.808	1	.369		
Likelihood Ratio	1.261	1	.261		
Fisher's Exact Test				.359	.184
Linear-by-Linear Association	1.259	1	.262		
N of Valid Cases	91				

a. 0 cells (0.0%) have expected count less than 5. The minimum expected count is 11.54.

b. Computed only for a 2x2 table

Portal_lymphnode * STAT3

Crosstab

			STAT3		Total
			0	1	
Portal_lymphnode	0	Count	37	24	61
		% within STAT3	66.1%	68.6%	67.0%
	1	Count	19	11	30
		% within STAT3	33.9%	31.4%	33.0%
Total		Count	56	35	91
		% within STAT3	100.0%	100.0%	100.0%

Chi-Square Tests

	Value	df	Asymptotic Significance (2-sided)	Exact Sig. (2-sided)	Exact Sig. (1-sided)
Pearson Chi-Square	.061[a]	1	.805		
Continuity Correction[b]	.000	1	.986		
Likelihood Ratio	.061	1	.805		
Fisher's Exact Test				1.000	.495
Linear-by-Linear Association	.060	1	.806		
N of Valid Cases	91				

a. 0 cells (0.0%) have expected count less than 5. The minimum expected count is 11.54.

b. Computed only for a 2x2 table

Portal_lymphnode * CK19

Crosstab

			CK19		Total
			0	1	
Portal_lymphnode	0	Count	34	27	61
		% within CK19	69.4%	64.3%	67.0%
	1	Count	15	15	30
		% within CK19	30.6%	35.7%	33.0%
Total		Count	49	42	91
		% within CK19	100.0%	100.0%	100.0%

Chi-Square Tests

	Value	df	Asymptotic Significance (2-sided)	Exact Sig. (2-sided)	Exact Sig. (1-sided)
Pearson Chi-Square	.266[a]	1	.606		
Continuity Correction[b]	.086	1	.770		
Likelihood Ratio	.266	1	.606		
Fisher's Exact Test				.659	.384
Linear-by-Linear Association	.263	1	.608		
N of Valid Cases	91				

a. 0 cells (0.0%) have expected count less than 5. The minimum expected count is 13.85.

b. Computed only for a 2x2 table

Portal_lymphnode * GP130

Crosstab

			GP130		Total
			0	1	
Portal_lymphnode	0	Count	36	25	61
		% within GP130	65.5%	69.4%	67.0%
	1	Count	19	11	30
		% within GP130	34.5%	30.6%	33.0%
Total		Count	55	36	91
		% within GP130	100.0%	100.0%	100.0%

Chi-Square Tests

	Value	df	Asymptotic Significance (2-sided)	Exact Sig. (2-sided)	Exact Sig. (1-sided)
Pearson Chi-Square	.157[a]	1	.692		
Continuity Correction[b]	.028	1	.867		
Likelihood Ratio	.157	1	.691		
Fisher's Exact Test				.820	.436
Linear-by-Linear Association	.155	1	.694		
N of Valid Cases	91				

a. 0 cells (0.0%) have expected count less than 5. The minimum expected count is 11.87.

b. Computed only for a 2x2 table

Portal_lymphnode * JAK2

Crosstab

			JAK2		Total
			0	1	
Portal_lymphnode	0	Count	41	20	61
		% within JAK2	70.7%	60.6%	67.0%
	1	Count	17	13	30
		% within JAK2	29.3%	39.4%	33.0%
Total		Count	58	33	91
		% within JAK2	100.0%	100.0%	100.0%

Chi-Square Tests

	Value	df	Asymptotic Significance (2-sided)	Exact Sig. (2-sided)	Exact Sig. (1-sided)
Pearson Chi-Square	.968[a]	1	.325		
Continuity Correction[b]	.565	1	.452		
Likelihood Ratio	.957	1	.328		
Fisher's Exact Test				.360	.225
Linear-by-Linear Association	.957	1	.328		
N of Valid Cases	91				

a. 0 cells (0.0%) have expected count less than 5. The minimum expected count is 10.88.

b. Computed only for a 2x2 table

Portal_lymphnode * IL6

Crosstab

			IL6		Total
			0	1	
Portal_lymphnode	0	Count	30	31	61
		% within IL6	63.8%	70.5%	67.0%
	1	Count	17	13	30
		% within IL6	36.2%	29.5%	33.0%
Total		Count	47	44	91
		% within IL6	100.0%	100.0%	100.0%

Chi-Square Tests

	Value	df	Asymptotic Significance (2-sided)	Exact Sig. (2-sided)	Exact Sig. (1-sided)
Pearson Chi-Square	.451[a]	1	.502		
Continuity Correction[b]	.201	1	.654		
Likelihood Ratio	.452	1	.501		
Fisher's Exact Test				.514	.327
Linear-by-Linear Association	.446	1	.504		
N of Valid Cases	91				

a. 0 cells (0.0%) have expected count less than 5. The minimum expected count is 14.51.

b. Computed only for a 2x2 table

```
CROSSTABS
/TABLES= Distant_lymphnode_metastasis BY IL6R CRP STAT3 CK19 GP130 JAK2 IL6
/FORMAT= AVALUE TABLES
/STATISTICS = CHISQ
/CELLS= COUNT COLUMN
/COUNT ROUND CELL.
```

Crosstabs

Notes

Output Created		25-JAN-2024 09:04:50
Comments		
Input	Data	D:\D\project\IL6 and BTC\manuscript\frontiers in immunology\Expression and prognosis.sav
	Active Dataset	1
	Filter	<none>
	Weight	<none>
	Split File	<none>
	N of Rows in Working Data File	91
Missing Value Handling	Definition of Missing	User-defined missing values are treated as missing.
	Cases Used	Statistics for each table are based on all the cases with valid data in the specified range(s) for all variables in each table.
Syntax		CROSSTABS<br><br>/TABLES=Distant_lymphnode_metastasis BY IL6R CRP STAT3 CK19 GP130 JAK2 IL6<br>/FORMAT=AVALUE TABLES<br>/STATISTICS=CHISQ<br>/CELLS=COUNT COLUMN<br>/COUNT ROUND CELL.
Resources	Processor Time	00:00:00.02
	Elapsed Time	00:00:00.01
	Dimensions Requested	2
	Cells Available	524245

Case Processing Summary

	Cases					
	Valid		Missing		Total	
	N	Percent	N	Percent	N	Percent
Distant_lymphnode_metastasis * IL6R	91	100.0%	0	0.0%	91	100.0%
Distant_lymphnode_metastasis * CRP	91	100.0%	0	0.0%	91	100.0%
Distant_lymphnode_metastasis * STAT3	91	100.0%	0	0.0%	91	100.0%
Distant_lymphnode_metastasis * CK19	91	100.0%	0	0.0%	91	100.0%
Distant_lymphnode_metastasis * GP130	91	100.0%	0	0.0%	91	100.0%
Distant_lymphnode_metastasis * JAK2	91	100.0%	0	0.0%	91	100.0%
Distant_lymphnode_metastasis * IL6	91	100.0%	0	0.0%	91	100.0%

Distant_lymphnode_metastasis * IL6R

Crosstab

			IL6R		Total
			0	1	
Distant_lymphnode_metastasis	0	Count	37	44	81
		% within IL6R	82.2%	95.7%	89.0%
	1	Count	8	2	10
		% within IL6R	17.8%	4.3%	11.0%
Total		Count	45	46	91
		% within IL6R	100.0%	100.0%	100.0%

Chi-Square Tests

	Value	df	Asymptotic Significance (2-sided)	Exact Sig. (2-sided)	Exact Sig. (1-sided)
Pearson Chi-Square	4.194[a]	1	.041		
Continuity Correction[b]	2.934	1	.087		
Likelihood Ratio	4.450	1	.035		
Fisher's Exact Test				.050	.042
Linear-by-Linear Association	4.148	1	.042		
N of Valid Cases	91				

a. 1 cells (25.0%) have expected count less than 5. The minimum expected count is 4.95.

b. Computed only for a 2x2 table

Distant_lymphnode_metastasis * CRP

Crosstab

			CRP 0	CRP 1	Total
Distant_lymphnode_metastasis	0	Count	48	33	81
		% within CRP	85.7%	94.3%	89.0%
	1	Count	8	2	10
		% within CRP	14.3%	5.7%	11.0%
Total		Count	56	35	91
		% within CRP	100.0%	100.0%	100.0%

Chi-Square Tests

	Value	df	Asymptotic Significance (2-sided)	Exact Sig. (2-sided)	Exact Sig. (1-sided)
Pearson Chi-Square	1.618[a]	1	.203		
Continuity Correction[b]	.860	1	.354		
Likelihood Ratio	1.759	1	.185		
Fisher's Exact Test				.306	.178
Linear-by-Linear Association	1.600	1	.206		
N of Valid Cases	91				

a. 1 cells (25.0%) have expected count less than 5. The minimum expected count is 3.85.

b. Computed only for a 2x2 table

Distant_lymphnode_metastasis * STAT3

Crosstab

			STAT3 0	STAT3 1	Total
Distant_lymphnode_metastasis	0	Count	48	33	81
		% within STAT3	85.7%	94.3%	89.0%
	1	Count	8	2	10
		% within STAT3	14.3%	5.7%	11.0%
Total		Count	56	35	91
		% within STAT3	100.0%	100.0%	100.0%

Chi-Square Tests

	Value	df	Asymptotic Significance (2-sided)	Exact Sig. (2-sided)	Exact Sig. (1-sided)
Pearson Chi-Square	1.618[a]	1	.203		
Continuity Correction[b]	.860	1	.354		
Likelihood Ratio	1.759	1	.185		
Fisher's Exact Test				.306	.178
Linear-by-Linear Association	1.600	1	.206		
N of Valid Cases	91				

a. 1 cells (25.0%) have expected count less than 5. The minimum expected count is 3.85.

b. Computed only for a 2x2 table

Distant_lymphnode_metastasis * CK19

Crosstab

			CK19		Total
			0	1	
Distant_lymphnode_metastasis	0	Count	46	35	81
		% within CK19	93.9%	83.3%	89.0%
	1	Count	3	7	10
		% within CK19	6.1%	16.7%	11.0%
Total		Count	49	42	91
		% within CK19	100.0%	100.0%	100.0%

Chi-Square Tests

	Value	df	Asymptotic Significance (2-sided)	Exact Sig. (2-sided)	Exact Sig. (1-sided)
Pearson Chi-Square	2.571[a]	1	.109		
Continuity Correction[b]	1.606	1	.205		
Likelihood Ratio	2.605	1	.107		
Fisher's Exact Test				.178	.103
Linear-by-Linear Association	2.542	1	.111		
N of Valid Cases	91				

a. 1 cells (25.0%) have expected count less than 5. The minimum expected count is 4.62.

b. Computed only for a 2x2 table

Distant_lymphnode_metastasis * GP130

Crosstab

			GP130		Total
			0	1	
Distant_lymphnode_metastasis	0	Count	50	31	81
		% within GP130	90.9%	86.1%	89.0%
	1	Count	5	5	10
		% within GP130	9.1%	13.9%	11.0%
Total		Count	55	36	91
		% within GP130	100.0%	100.0%	100.0%

Chi-Square Tests

	Value	df	Asymptotic Significance (2-sided)	Exact Sig. (2-sided)	Exact Sig. (1-sided)
Pearson Chi-Square	.512[a]	1	.474		
Continuity Correction[b]	.139	1	.709		
Likelihood Ratio	.502	1	.479		
Fisher's Exact Test				.509	.349
Linear-by-Linear Association	.506	1	.477		
N of Valid Cases	91				

a. 1 cells (25.0%) have expected count less than 5. The minimum expected count is 3.96.

b. Computed only for a 2x2 table

Distant_lymphnode_metastasis * JAK2

Crosstab

			JAK2		Total
			0	1	
Distant_lymphnode_metastasis	0	Count	53	28	81
		% within JAK2	91.4%	84.8%	89.0%
	1	Count	5	5	10
		% within JAK2	8.6%	15.2%	11.0%
Total		Count	58	33	91
		% within JAK2	100.0%	100.0%	100.0%

Chi-Square Tests

	Value	df	Asymptotic Significance (2-sided)	Exact Sig. (2-sided)	Exact Sig. (1-sided)
Pearson Chi-Square	.917[a]	1	.338		
Continuity Correction[b]	.371	1	.542		
Likelihood Ratio	.886	1	.347		
Fisher's Exact Test				.487	.267
Linear-by-Linear Association	.907	1	.341		
N of Valid Cases	91				

a. 1 cells (25.0%) have expected count less than 5. The minimum expected count is 3.63.

b. Computed only for a 2x2 table

Distant_lymphnode_metastasis * IL6

Crosstab

			IL6		Total
			0	1	
Distant_lymphnode_metastasis	0	Count	42	39	81
		% within IL6	89.4%	88.6%	89.0%
	1	Count	5	5	10
		% within IL6	10.6%	11.4%	11.0%
Total		Count	47	44	91
		% within IL6	100.0%	100.0%	100.0%

Chi-Square Tests

	Value	df	Asymptotic Significance (2-sided)	Exact Sig. (2-sided)	Exact Sig. (1-sided)
Pearson Chi-Square	.012[a]	1	.912		
Continuity Correction[b]	.000	1	1.000		
Likelihood Ratio	.012	1	.912		
Fisher's Exact Test				1.000	.587
Linear-by-Linear Association	.012	1	.912		
N of Valid Cases	91				

a. 1 cells (25.0%) have expected count less than 5. The minimum expected count is 4.84.

b. Computed only for a 2x2 table

```
CROSSTABS
/TABLES=Vascular_invasion BY IL6R CRP STAT3 CK19 GP130 JAK2 IL6
```

```
/FORMAT=AVALUE TABLES
/STATISTICS=CHISQ
/CELLS=COUNT COLUMN
/COUNT ROUND CELL.
```

Crosstabs

Notes

Output Created		25-JAN-2024 09:05:24
Comments		
Input	Data	D:\D\project\IL6 and BTC\manuscript\frontiers in immunology\Expression and prognosis.sav
	Active Dataset	1
	Filter	<none>
	Weight	<none>
	Split File	<none>
	N of Rows in Working Data File	91
Missing Value Handling	Definition of Missing	User-defined missing values are treated as missing.
	Cases Used	Statistics for each table are based on all the cases with valid data in the specified range(s) for all variables in each table.
Syntax		CROSSTABS /TABLES=Vascular_invasion BY IL6R CRP STAT3 CK19 GP130 JAK2 IL6 /FORMAT=AVALUE TABLES /STATISTICS=CHISQ /CELLS=COUNT COLUMN /COUNT ROUND CELL.
Resources	Processor Time	00:00:00.02
	Elapsed Time	00:00:00.01
	Dimensions Requested	2
	Cells Available	524245

Case Processing Summary

	Cases					
	Valid		Missing		Total	
	N	Percent	N	Percent	N	Percent
Vascular_invasion * IL6R	91	100.0%	0	0.0%	91	100.0%
Vascular_invasion * CRP	91	100.0%	0	0.0%	91	100.0%
Vascular_invasion * STAT3	91	100.0%	0	0.0%	91	100.0%
Vascular_invasion * CK19	91	100.0%	0	0.0%	91	100.0%
Vascular_invasion * GP130	91	100.0%	0	0.0%	91	100.0%
Vascular_invasion * JAK2	91	100.0%	0	0.0%	91	100.0%
Vascular_invasion * IL6	91	100.0%	0	0.0%	91	100.0%

Vascular_invasion * IL6R

Crosstab

			IL6R		Total
			0	1	
Vascular_invasion	0	Count	30	32	62
		% within IL6R	66.7%	69.6%	68.1%
	1	Count	15	14	29
		% within IL6R	33.3%	30.4%	31.9%
Total		Count	45	46	91
		% within IL6R	100.0%	100.0%	100.0%

Chi-Square Tests

	Value	df	Asymptotic Significance (2-sided)	Exact Sig. (2-sided)	Exact Sig. (1-sided)
Pearson Chi-Square	.088[a]	1	.767		
Continuity Correction[b]	.005	1	.943		
Likelihood Ratio	.088	1	.767		
Fisher's Exact Test				.824	.471
Linear-by-Linear Association	.087	1	.768		
N of Valid Cases	91				

a. 0 cells (0.0%) have expected count less than 5. The minimum expected count is 14.34.

b. Computed only for a 2x2 table

Vascular_invasion * CRP

Crosstab

			CRP		Total
			0	1	
Vascular_invasion	0	Count	33	29	62
		% within CRP	58.9%	82.9%	68.1%
	1	Count	23	6	29
		% within CRP	41.1%	17.1%	31.9%
Total		Count	56	35	91
		% within CRP	100.0%	100.0%	100.0%

Chi-Square Tests

	Value	df	Asymptotic Significance (2-sided)	Exact Sig. (2-sided)	Exact Sig. (1-sided)
Pearson Chi-Square	5.680[a]	1	.017		
Continuity Correction[b]	4.631	1	.031		
Likelihood Ratio	6.001	1	.014		
Fisher's Exact Test				.021	.014
Linear-by-Linear Association	5.617	1	.018		
N of Valid Cases	91				

a. 0 cells (0.0%) have expected count less than 5. The minimum expected count is 11.15.

b. Computed only for a 2x2 table

Vascular_invasion * STAT3

Crosstab

			STAT3		Total
			0	1	
Vascular_invasion	0	Count	34	28	62
		% within STAT3	60.7%	80.0%	68.1%
	1	Count	22	7	29
		% within STAT3	39.3%	20.0%	31.9%
Total		Count	56	35	91
		% within STAT3	100.0%	100.0%	100.0%

Chi-Square Tests

	Value	df	Asymptotic Significance (2-sided)	Exact Sig. (2-sided)	Exact Sig. (1-sided)
Pearson Chi-Square	3.690[a]	1	.055		
Continuity Correction[b]	2.855	1	.091		
Likelihood Ratio	3.839	1	.050		
Fisher's Exact Test				.067	.044
Linear-by-Linear Association	3.649	1	.056		
N of Valid Cases	91				

a. 0 cells (0.0%) have expected count less than 5. The minimum expected count is 11.15.

b. Computed only for a 2x2 table

Vascular_invasion * CK19

Crosstab

			CK19		Total
			0	1	
Vascular_invasion	0	Count	33	29	62
		% within CK19	67.3%	69.0%	68.1%
	1	Count	16	13	29
		% within CK19	32.7%	31.0%	31.9%
Total		Count	49	42	91
		% within CK19	100.0%	100.0%	100.0%

Chi-Square Tests

	Value	df	Asymptotic Significance (2-sided)	Exact Sig. (2-sided)	Exact Sig. (1-sided)
Pearson Chi-Square	.030[a]	1	.862		
Continuity Correction[b]	.000	1	1.000		
Likelihood Ratio	.030	1	.862		
Fisher's Exact Test				1.000	.522
Linear-by-Linear Association	.030	1	.863		
N of Valid Cases	91				

a. 0 cells (0.0%) have expected count less than 5. The minimum expected count is 13.38.

b. Computed only for a 2x2 table

Vascular_invasion * GP130

Crosstab

			GP130		Total
			0	1	
Vascular_invasion	0	Count	33	29	62
		% within GP130	60.0%	80.6%	68.1%
	1	Count	22	7	29
		% within GP130	40.0%	19.4%	31.9%
Total		Count	55	36	91
		% within GP130	100.0%	100.0%	100.0%

Chi-Square Tests

	Value	df	Asymptotic Significance (2-sided)	Exact Sig. (2-sided)	Exact Sig. (1-sided)
Pearson Chi-Square	4.234[a]	1	.040		
Continuity Correction[b]	3.340	1	.068		
Likelihood Ratio	4.410	1	.036		
Fisher's Exact Test				.065	.032
Linear-by-Linear Association	4.188	1	.041		
N of Valid Cases	91				

a. 0 cells (0.0%) have expected count less than 5. The minimum expected count is 11.47.

b. Computed only for a 2x2 table

Vascular_invasion * JAK2

Crosstab

			JAK2		Total
			0	1	
Vascular_invasion	0	Count	35	27	62
		% within JAK2	60.3%	81.8%	68.1%
	1	Count	23	6	29
		% within JAK2	39.7%	18.2%	31.9%
Total		Count	58	33	91
		% within JAK2	100.0%	100.0%	100.0%

Chi-Square Tests

	Value	df	Asymptotic Significance (2-sided)	Exact Sig. (2-sided)	Exact Sig. (1-sided)
Pearson Chi-Square	4.467[a]	1	.035		
Continuity Correction[b]	3.533	1	.060		
Likelihood Ratio	4.711	1	.030		
Fisher's Exact Test				.038	.028
Linear-by-Linear Association	4.418	1	.036		
N of Valid Cases	91				

a. 0 cells (0.0%) have expected count less than 5. The minimum expected count is 10.52.

b. Computed only for a 2x2 table

Vascular_invasion * IL6

Crosstab

			IL6		Total
			0	1	
Vascular_invasion	0	Count	31	31	62
		% within IL6	66.0%	70.5%	68.1%
	1	Count	16	13	29
		% within IL6	34.0%	29.5%	31.9%
Total		Count	47	44	91
		% within IL6	100.0%	100.0%	100.0%

Chi-Square Tests

	Value	df	Asymptotic Significance (2-sided)	Exact Sig. (2-sided)	Exact Sig. (1-sided)
Pearson Chi-Square	.212[a]	1	.645		
Continuity Correction[b]	.055	1	.814		
Likelihood Ratio	.212	1	.645		
Fisher's Exact Test				.661	.408
Linear-by-Linear Association	.209	1	.647		
N of Valid Cases	91				

a. 0 cells (0.0%) have expected count less than 5. The minimum expected count is 14.02.

b. Computed only for a 2x2 table
